# Semaglutide versus placebo in individuals with poor weight loss after bariatric surgery: a double-blinded, randomized, placebo-controlled trial

**DOI:** 10.1038/s41591-026-04416-4

**Published:** 2026-05-22

**Authors:** Chloe Stanley, Ritwika Mallik, Nausheen Hamid, Friedrich C. Jassil, Victoria Reuven, Tapiwa Ruwona, Samuel J. Dicken, Sulmaaz Qamar, Benjamin Norton, Helen Kingett, Andrea Pucci, Cormac Magee, David Boniface, Rachel L. Batterham, Kalpana Devalia, Jessica Mok, Mohamed Elkalaawy, John Loy, Andrew Jenkinson, Haris Markakis, Marco Adamo, Alanna Brown, Janine Makaronidis

**Affiliations:** 1 Centre for Obesity Research, University College London, London, UK; 2 National Institute for Health Research (NIHR) University College London Hospital Biomedical Research Centre, London, UK; 3 Department of Diabetes and Metabolism, Barts Health NHS Trust, London, UK; 4 Blizard Institute, Queen Mary University of London, London, UK; 5 Bariatric Centre for Weight Management and Metabolic Surgery, University College London Hospital (UCLH), London, UK; 6 Department of Endocrinology, Diabetology, and Metabolism, Geneva University Hospitals, Geneva, Switzerland; 7 Institute for Clinical Diabetology, German Diabetes Center, Leibniz Center for Diabetes Research at Heinrich-Heine-University Düsseldorf, Düsseldorf, Germany; 8 Behavioural Science and Health, University College London, London, UK; 9 Digestive Disease & Surgery Institute, Cleveland Clinic London, London, UK; 10 Ashford and St Peter’s Hospitals NHS Foundation Trust, Surrey, UK; 11 Homerton University Hospitals, London, UK; 12 Auralia Clinic, Dublin, Ireland

**Keywords:** Randomized controlled trials, Obesity

## Abstract

A suboptimal clinical response after metabolic and bariatric surgery (MBS) is common and carries considerable health concerns. Here a double-blinded, randomized (1:1), placebo-controlled trial using semaglutide 2.4 mg weekly (BARI-STEP) recruited adult participants at least 1 year after gastric bypass or sleeve gastrectomy with a suboptimal clinical response, defined as less than 20% weight loss from surgery. This was an adjunct to lifestyle intervention with a 500-kcal daily energy deficit. The primary outcome was percentage weight loss after 68-week treatment on an intention-to-treat analysis. Seventy participants (mean (s.d.) age = 47.3 (10.3) years, 58 (82.9%) female and 12 (17.1%) male) were randomized to receive 2.4 mg semaglutide (*n* = 35) or placebo (*n* = 35). The intention-to-treat sample included 63 participants. Estimated change in mean (s.d.) percentage weight loss from baseline to week 68 was −18.0 (9.2) with semaglutide 2.4 mg (*n* = 34) versus +0.4 (7.0) with placebo (*n* = 29). The mean adjusted treatment difference in percentage body weight change for semaglutide 2.4 mg versus placebo was −19.18 (95% confidence interval −23.4 to −14.8; *P* < 0.001). Adverse events (AEs) were consistent with the known safety and tolerability profile of semaglutide, with no new safety concerns for the post-bariatric population. There were eight serious AEs, one suspected unexpected serious adverse reaction and no treatment-related deaths. BARI-STEP demonstrates that in people with a suboptimal clinical response after MBS, semaglutide results in substantial and clinically significant body weight reduction along with improvement in metabolic parameters and quality of life, compared to placebo. These findings suggest that semaglutide 2.4 mg is a safe and effective treatment option for this patient population. ClinicalTrials.gov: NCT05073835.

## Abstract (web summary)

At week 68, in patients who experienced poor weight loss following bariatric surgery, semaglutide was associated with 18.0% weight loss compared to 0.4% weight gain in patients receiving placebo.

## Main

Obesity and its associated comorbidities represent a global health threat, contributing to 3.7 million deaths annually^1^. The emergence of highly effective new glucagon-like peptide-1 (GLP-1)-based pharmacotherapy agents for the management of obesity and its complications is shifting the treatment algorithms for weight management. However, metabolic and bariatric surgery (MBS) remains the most effective treatment for patients with severe obesity in the long term, producing sustained weight loss with reduced morbidity and mortality. Over the past two decades, MBS has been widely adopted with ~500,000 operations undertaken annually worldwide^2^. In the UK, patients with severe obesity, defined as a body mass index (BMI) of ≥40 kg m^−2^, or ≥35 kg m^−2^ (reduced by 2.5 kg m^−^^2^ for certain ethnicities) with an obesity associated comorbidity, are eligible for MBS in accordance with National Institute for Health and Care Excellence guidelines^3^. Roux-en-Y gastric bypass (RYGB) and sleeve gastrectomy (SG) are the most common bariatric procedures performed globally^2^.

While at a population level MBS is highly effective, at the level of the individual, weight loss is highly variable and follows a normal distribution^4,5^. Approximately 20% of patients undergoing MBS have a suboptimal clinical response. Importantly, improvement and resolution in weight-related comorbidities are weight-loss dependent. Patients with a suboptimal clinical response after MBS have fewer metabolic benefits and recurrent weight gain leads to the re-emergence of comorbidities^6–9^. For example, we previously observed that type 2 diabetes (T2D) remission at 2-years after surgery (defined as hemoglobin A1c (HbA1c) < 6%, off all medication for more than 12 months) correlated with percentage weight loss (%WL), independent of the type of procedure^10^. In addition, greater improvements in quality-of-life outcomes after bariatric surgery are reported with greater weight loss^11,12^. These findings highlight a strong clinical need for maximizing weight loss after bariatric surgery. However, the weight management options available for this patient population are limited, which is growing in number given the wide adoption of MBS globally, calling for effective treatment strategies. Historically, revisional surgical interventions for weight regain were the only option in treatment paradigms; however, they have shown only modest impacts on body weight and metabolic health. In contrast, pharmacological options have recently shown promise as a therapeutic option to maximize the health benefits of MBS^13–15^.

Several factors have been proposed to explain the variability in weight loss response after MBS, including genetics^16^, psychological factors^17^ and central regulatory pathways. Alternatively, or in addition, low meal-stimulated circulating levels of the gut hormone GLP-1 have been linked to poor responses to MBS^18^, suggesting that GLP-1 receptor agonism may have a role in the treatment of this patient population. The GRAVITAS trial^14^ demonstrated that, in individuals with persistent or recurrent T2D after MBS, liraglutide 1.8 mg daily as an adjunct to lifestyle intervention, improved glycemic control with a mean weight difference of −4.2 kg (95% confidence interval (CI) −6.8 to −1.4) after 26 weeks. Subsequently, we conducted the BARI-OPTIMISE trial, a 24-week randomized-controlled, double-blind trial of liraglutide 3.0 mg versus placebo as an adjunct to diet and lifestyle counseling in patients with suboptimal weight loss after RYGB or SG and a blunted meal-stimulated GLP-1 response. Liraglutide 3.0 mg resulted in clinically relevant weight loss of 9.4% at 24 weeks compared to 1.1% with placebo (estimated treatment effect of −8.8; 95% CI −11.9 to −5.7; *P* < 0.005). Liraglutide 3.0 mg represented an effective, safe treatment to improve weight and health in patients with suboptimal weight loss after MBS^13^.

Semaglutide is a long-acting GLP-1 receptor agonist (GLP-1RA) with robust evidence for its role in weight management and treatment of metabolic comorbidities in adults with obesity. The results of the STEP program led to the approval of semaglutide 2.4 mg once weekly for weight management in adults with obesity or overweight with at least one weight-related comorbidity, alongside a reduced-calorie diet and increased physical activity, in 2021. Semaglutide 2.4 mg leads to superior weight-reducing efficacy compared to liraglutide 3.0 mg in people with overweight or obesity without diabetes (−15.8% with semaglutide versus −6.4% with liraglutide at 68 weeks)^19^. Importantly, the SELECT trial demonstrated a reduction in cardiovascular mortality and incidence of nonfatal myocardial infarction and stroke after treatment with semaglutide 2.4 mg at 39.8 months, independent of weight loss^20^.

To our knowledge, there is no randomized clinical trial evaluating the safety and efficacy of semaglutide 2.4 mg for a suboptimal clinical response after MBS. Considering the promising results from the BARI-OPTIMISE trial and the STEP program, we hypothesized that once-weekly semaglutide 2.4 mg will lead to greater weight loss and health improvement compared to placebo in patients with a suboptimal clinical response after MBS.

The BARI-STEP trial is a 68-week double-blind, randomized, placebo-controlled trial to assess the efficacy of once-weekly semaglutide 2.4 mg in people with suboptimal weight loss 1 year or longer after GB or SG, in terms of %WL, the number of patients achieving categorical weight loss (<10%, >10%, >15% and >20%), metabolic outcomes and quality of life parameters. BARI-STEP also examined the relationship between endogenous meal-stimulated GLP-1 levels and response to treatment with semaglutide 2.4 mg weekly.

## Results

**Fig. 1:**
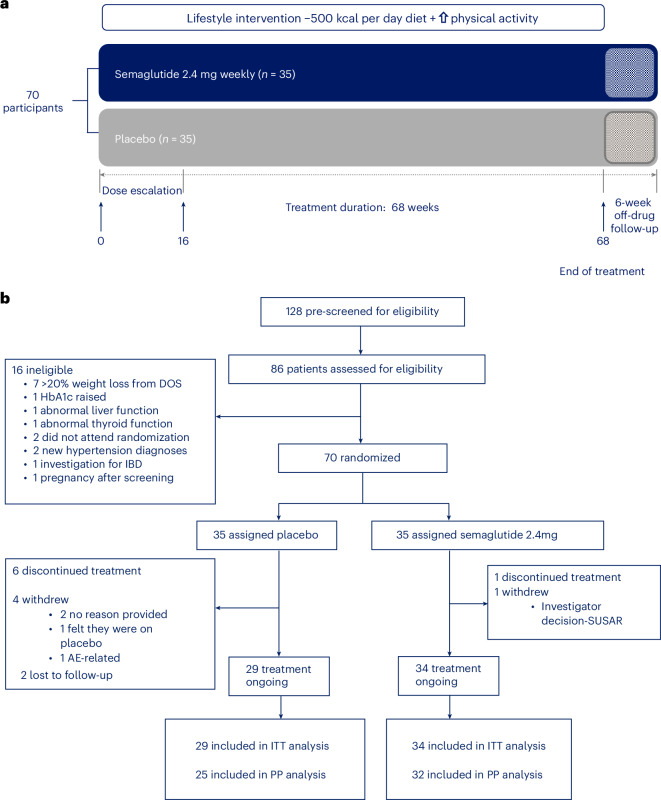
BARI-STEP trial design and CONSORT diagram. **a**, Trial design. **b**, CONSORT diagram. IBD, inflammatory bowel disease.

The study was conducted between 1 November 2022 and 9 April 2025. A total of 128 participants were pre-screened for eligibility, 86 screened for eligibility and 70 (mean age 47.3 years, s.d. ± 10.3; 58 (82.9%) female and 12 (17.1%) male) were randomized: 35 to semaglutide 2.4 mg once weekly plus lifestyle intervention and 35 to placebo plus lifestyle intervention. The trial design and Consolidated Standards of Reporting Trials (CONSORT) diagram is illustrated in Fig. 1. The baseline characteristics of the trial population were comparable between groups (Table 1).

**Table 1:** Baseline characteristics

Mean (s.d.)/*n* (%)	Placebo (*n* = 35)	Semaglutide 2.4 mg (*n* = 35)	Overall trial (*n* = 70)
Age (years)	47.7 (10.3)	46.9 (10.4)	47.3 (10.3)
Sex, *n* (%)			
Female	28 (80.0)	30 (85.7)	58 (82.9)
Male	7 (20.0)	5 (14.3)	12 (17.1)
Weight (kg)	111.4 (19.3)	118.6 (23.3)	115 (12.6)
BMI	40.2 (6.0)	42.8 (6.8)	41.5 (6.5)
BMI, kg m^−^^2^, *n* (%)			
<35	5 (14.3)	2 (5.8)	7 (10)
≥35 to <40	15 (42.9)	14 (40.0)	29 (41.4)
≥40	15 (42.9)	19 (54.3)	34 (48.6)
Weight on DOS (kg)	122.9 (20.2)	126.1 (23.0)	124.5 (21.5)
BMI on DOS (kg m^−^^2^)	44.3 (5.8)	45.6 (7.0)	44.9 (6.4)
Post-surgery maximum %WL	21.4 (9.6)	24.7 (8.3)	23.1 (9.1)
Nadir BMI (kg m^−^^2^)	34.7 (5.5)	34.2 (6.0)	34.5 (5.8)
%WL since DOS	9.2 (6.7)	5.8 (8.2)	7.5 (7.6)
Post-surgery outcome			
Weight regain, *n* (%)	17 (48.6)	22 (62.9)	39 (55.7)
Suboptimal initial weight loss, *n* (%)	18 (51.4)	13 (37.1)	31 (44.3)
Ethnicity, *n* (%)			
Asian	0 (0.0)	2 (5.7)	2 (2.9)
Black	10 (28.6)	6 (17.1)	16 (22.9)
White	21 (60.0)	26 (74.3)	47 (67.1)
Mixed/other	4 (11.4)	1 (2.9)	5 (7.1)
Surgical procedure *n* (%)			
SG	28 (80.0)	27 (77.1)	55 (78.6)
GB	7 (20.0)	8 (22.9)	15 (21.4)
Time since surgery (months)	79.1 (34.2)	88.6 (50.5)	83.9 (43.1)
Diabetes status, *n* (%)			
T2D	4 (11.4)	3 (8.6)	7 (10.0)
Normoglycemia/pre-diabetes	31 (88.6)	32 (91.4)	63 (90.0)
HbA1c (mmol mol^−1^)	38.9 (4.2)	37.1 (5.6)^a^	38.0 (5.0)
Change in meal-stimulated GLP-1 (pg ml^−1^)	10.5 (3.3, 29.6)^a^	6.3 (2.9, 26.8)^a^	7.6 (3.0, 29.5)

Gastric bypass included standardized Roux-and-Y gastric bypass and one-anastomosis gastric bypass. ^a^*n* = 34.

### Adherence

Two participants (both in the placebo arm) were lost to follow-up. Five participants withdrew from the trial, including four in the placebo arm (two participants did not provide a reason for withdrawal, one participant did not have a weight loss response, and the remaining participant withdrew due to adverse event (AE) reasons). One participant in the semaglutide group was withdrawn because of an investigator decision due to a suspected unexpected serious adverse reaction (SUSAR).

Twenty-nine participants in the placebo group and 34 in the semaglutide arm completed the trial, meeting the target sample size for the primary outcome analysis. Baseline characteristics for patients included in the primary outcome analysis (intention-to-treat (ITT), *n* = 63) are reported in Supplementary Table 1.

All participants who completed the trial (ITT population) reached the target dose of semaglutide 2.4 mg (*n* = 34). However, six participants were maintained on a reduced dose of semaglutide because of AEs (1.7 mg, *n* = 1; 1.0 mg, *n* = 2; 0.5 mg, *n* = 2 and one participant reduced to 1.7 mg before withdrawing).

### Definition of the analysis populations

#### ITT and full analysis set

For the ITT sample, all randomized participants were used for the analyses except those who withdrew or were lost to follow-up. Six participants were included in the ITT but excluded from the per-protocol (PP) analysis because they did not satisfy the definition of full adherence and therefore did not meet the conditions to be included in PP analysis. Reasons included a prolonged time off drug treatment (*n* = 5) and an apronectomy (*n* = 1) during the trial.

#### PP analysis

Participants in the PP analysis followed the study protocol with full adherence, defined as attending all study visits with no substantial interruptions in treatment as monitored from the drug dose diary.

### Safety

Safety reporting (that is, AEs and SAEs) and analyses included all randomized participants (*n* = 70). All participants received at least one dose of the assigned treatment.

### Primary outcome: %WL

**Fig. 2:**
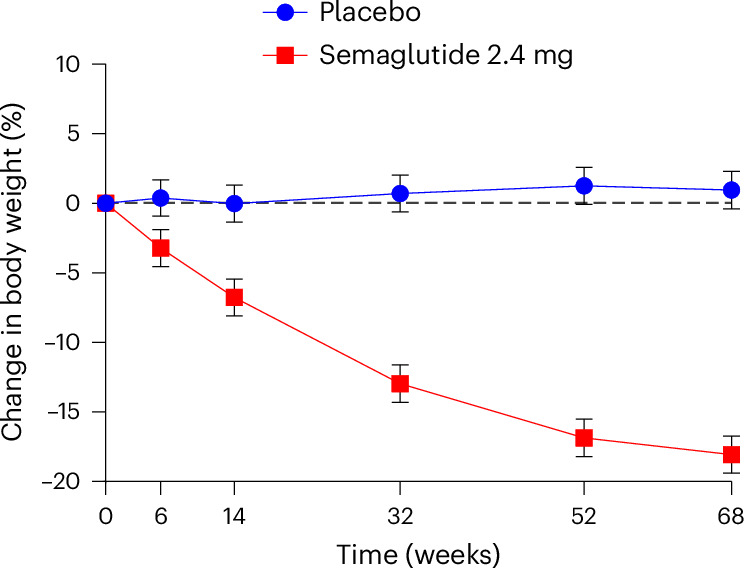
Effect of semaglutide 2.4 mg once-weekly versus placebo over time. Adjusted mean change in body weight (%) over time estimated using a repeated-measures, mixed-effects model adjusting for baseline weight, type of surgery, sex, diabetes status and interaction between treatment and visit (*n* = 70 all participants using all available data). The error bars indicate the s.e.m. Two-sided *t*-statistic with 95% CIs. Week 6: *n* = 70; d.f. = 134; *P* = 0.008. Week 14: *n* = 66; d.f. = 141, *P* < 0.001. Week 32: *n* = 64; d.f. = 144, *P* < 0.001. Week 52: *n* = 61; d.f. = 151, *P* < 0.001. Week 68: *n* = 63; d.f. = 147, *P* < 0.001, not adjusted for multiple comparisons.

At 68 weeks, participants in the semaglutide group (*n* = 34) experienced on average 18.0% weight loss (s.d. = 9.2), compared to the placebo group (*n* = 29) who experienced a mean weight gain of 0.4% (s.d. = 7.0). In the primary outcome analysis, the adjusted mean %WL of participants in the semaglutide group was −18.2% (s.e. = 2.3), and a mean weight gain of 0.9% (s.e. = 2.2) in the placebo group. The mean adjusted treatment difference for %WL in placebo versus semaglutide at 68 weeks was −19.1% (95% CI −23.4 to −14.8) (*P* < 0.001) (Fig. 2). A waterfall plot of %WL between groups is illustrated in Supplementary Fig. 1.

Participants in the semaglutide group lost on average −20.7 kg (s.d. = 10.2), compared to a mean weight gain of 0.5 kg (s.d. = 8.0) in the placebo group. The adjusted mean total body weight change in the semaglutide group was −21.1 kg (s.e. = 2.6) compared to 0.7 kg (s.e. = 2.5) in the placebo group. The adjusted difference in mean total body weight change (kg) between groups was −21.7 kg (95% CI −26.6 to −16.8) (*P* < 0.001).

### Categorical weight loss

A total of 93.1% (*n* = 27) of participants in the placebo group lost less than 10% weight loss, compared to 14.7% (*n* = 5) in the semaglutide group; 10% or more weight loss was achieved by 6.9% (*n* = 2) in the placebo group, compared to 85.3% (*n* = 29) in the semaglutide group. Similarly, 6.9% (*n* = 2) in the placebo group experienced 15% or more weight loss compared with 61.8% (*n* = 21) of the semaglutide group. Lastly, 3.4% (*n* = 1) of the placebo group experienced 20% or more weight loss compared with 47.1% (*n* = 16) of the semaglutide group (Supplementary Table 2 and Supplementary Fig. 2). Participants in the semaglutide group were significantly more likely to be within a higher weight loss category than the placebo group (*P* < 0.001).

Secondary outcomes are reported in Table 2. Repeated-measures analyses of secondary outcomes are reported in Supplementary Table 3. Unadjusted values of changes in secondary outcomes are reported in Supplementary Table 4.

**Table 2:** Changes in secondary outcomes at 68 weeks from baseline

	Placebo		Semaglutide 2.4 mg			
Outcome (ITT, *n* = 63)	No.	Mean (s.e.)	No.	Mean (s.e.)	Adjusted mean difference^a^	*P*^a^
Total body weight (kg)	29	0.7 (2.5)	34	−21.1 (2.6)	−21.7 (−26.6 to −16.8)	<0.001
BMI	28	0.03 (1.0)	33	−7.9 (1.0)	−7.9 (–9.8 to −6.0)	<0.001
Fat mass (kg)	28	0.9 (2.3)	33	−15.8 (2.3)	−16.6 (−20.8 to −12.5)	<0.001
Lean soft tissue mass (%)	28	−0.8 (1.2)	33	5.5 (1.2)	6.3 (4.1 to 8.5)	<0.001
Lean soft tissue mass (kg)	28	−0.2 (1.2)	33	−5.5 (1.2)	−5.3 (−7.4 to −3.1)	<0.001
HbA1c (mmol mol^−1^)	28	1.7 (0.8)	33	−3.6 (0.8)	−5.3 (−6.6 to −4.0)	<0.001
HR (bpm)	28	−6.6 (2.4)	34	−4.9 (2.7)	1.8 (−3.0 to 6.5)	0.460
SBP (mm Hg)	28	2.0 (3.1)	34	−3.9 (3.3)	−5.9 (−12.0 to 0.2)	0.059
DBP (mm Hg)	28	5.85 (2.3)	34	1.64 (2.5)	−4.2 (−8.7 to 0.3)	0.068
Total cholesterol (mmol l^−1^)	28	0.2 (0.2)	33	−0.3 (0.2)	−0.5 (−0.9 to −0.1)	0.014
Triglycerides (mmol l^−1^)	28	0.2 (0.1)	33	−0.3 (0.1)	−0.5 (−0.8 to −0.23)	<0.001
HsCRP mg l^−1^	28	1.4 (1.5)	31	0.4 (1.6)	−1.0 (−3.9 to 2.0)	0.517
IWQOL-Lite						
Total	27	9.3 (3.9)	33	23.7 (4.1)	14.3 (6.5 to 22.1)	<0.001
Physical function	27	7.3 (4.0)	34	23.0 (4.2)	15.7 (7.7 to 23.7)	<0.001
Self-esteem	27	17.3 (5.7)	34	34.2 (5.9)	16.9 (5.5 to 28.3)	0.004
Sex life	27	5.2 (6.4)	31	17.8 (6.5)	12.7 (−0.7 to 26.0)	0.062
Public distress	27	11.1 (5.6)	34	20.4 (5.8)	9.3 (−1.9 to 20.4)	0.103
Work	27	8.4 (5.0)	32	18.5 (5.1)	10.1 (0.1 to 20.1)	0.048

^a^Calculated from estimated marginal means from a linear regression adjusted for type of surgery, diabetes, sex, baseline weight (kg) and baseline value of the secondary outcome.

### BMI

Participants in the semaglutide group experienced an adjusted mean BMI change of −7.9 kg m^−^^2^ (s.e. = 1.0) compared to 0.03 kg m^−^^2^ (s.e. = 1.0) in the placebo group. The adjusted difference in mean BMI change (kg m^−^^2^) between groups was −7.9 kg m^−^^2^ (95% CI −9.8 to −6.0) (*P* < 0.001) (Table 2).

#### Body composition

In the semaglutide group, the adjusted mean fat mass change was −15.8 kg (s.e. = 2.3) compared to 0.9 kg (s.e. = 2.3) in the placebo group. The adjusted difference in mean fat mass change (kg) between groups was −16.6 kg (95% CI −20.8 to −12.5) (*P* < 0.001).

The adjusted mean change in lean soft tissue mass in the semaglutide group was −5.5 kg (s.e. = 1.2) compared to −0.2 kg (s.e. = 1.2) in the placebo group. The adjusted difference in mean lean soft tissue mass change (kg) between groups was −5.3 kg (95% CI −7.4 to −3.1) (*P* < 0.001).

The adjusted mean change in lean soft tissue mass (% of total body weight) in the semaglutide group was 5.5% (s.e. = 1.2) compared to −0.81 % (s.e. = 1.2) in the placebo group. The adjusted difference in mean lean soft tissue mass change (%) relative to total body weight between groups was 6.3 % (95% CI 4.1 to 8.5; *P* < 0.001).

#### Cardiovascular outcomes

The adjusted mean change in systolic blood pressure (SBP) in the semaglutide group was −3.9 mm Hg (s.e. = 3.3) compared to 2.0 mm Hg (s.e. = 3.1) in the placebo group. The adjusted difference in mean SBP (mm Hg) change between groups was −5.9 mm Hg (95% CI −12.0 to 0.2, *P* = 0.059).

The adjusted mean change in diastolic blood pressure (DBP) in the semaglutide group was −1.6 mm Hg (s.e. = 2.3) compared to 5.9 mm Hg (s.e. = 2.3) in the placebo group. The adjusted difference in mean DBP (mm Hg) change between groups was −4.2 mm Hg, (95% CI −8.8 to 0.3, *P* = 0.068) (Table 2). Mean adjusted changes in SBP and DBP over time are illustrated in Supplementary Fig. 3.

The adjusted mean change in heart rate (HR) in the semaglutide group was −4.9 bpm (s.e. = 2.7) compared to −6.6 bpm (s.e. = 2.4) in the placebo group. The adjusted difference in mean HR (bpm) change between groups was 1.8 bpm (95% CI −3.0 to 6.5, *P* = 0.460).

#### SBP and DBP in participants with pre-existing hypertension

The adjusted mean change in SBP in ITT participants with pre-existing hypertension in the semaglutide group (*n* = 10) was −2.2 mm Hg (s.e. = 3.9) compared to 9.4 mm Hg (s.e. = 3.8) in the placebo group (*n* = 9). The adjusted difference in mean SBP (mm Hg) change between groups in participants with pre-existing hypertension was −11.6 mm Hg.

The adjusted mean change in DBP in ITT participants with pre-existing hypertension in the semaglutide group was 6.3 mm Hg (s.e. = 3.2) compared to 7.9 mm Hg (s.e. = 2.9) in placebo. The adjusted difference in mean DBP (mm Hg) change between groups was −1.7 mm Hg.

#### Pharmacological agents required for the management of hypertension

The number of pharmacological agents required for hypertension management was assessed in ITT participants with pre-existing hypertension in the placebo (*n* = 9, 31.0%) and semaglutide 2.4 mg group (*n* = 10, 29.4%).

In the semaglutide group at baseline, nine of ten (90%) participants with pre-existing hypertension were on at least one antihypertensive medication, with six (60%) participants on one antihypertensive medication, three (30%) on two antihypertensive medications and one participant on none. At week 68, all participants with pre-existing hypertension had no change in the number of antihypertensive medications from baseline.

In the placebo group, nine (100%) participants with pre-existing hypertension at baseline were on at least one antihypertensive medication. Of nine participants, five (55.6%) were on one antihypertensive medication and the remaining four (44.4%) were on two antihypertensive medications. At week 68, six participants (66.7%) had no change, two participants (22.2%) increased and one participant (11.1%) decreased the number of antihypertensive medications from baseline.

Changes in the number of antihypertensive medications required for the management of hypertension at week 68 from baseline did not significantly differ between groups (*P* = 0.087).

#### HbA1c

The adjusted mean HbA1c change in the semaglutide group was −3.6 mmol mol^−1^ (s.e. = 0.8) compared to 1.7 mmol mol^−1^ (s.e. = 0.8) in the placebo group. The adjusted difference in mean HbA1c change (mmol mol^−1^) between groups was −5.3 mmol mol^−1^ (95% CI −6.6 to −4, *P* < 0.001) (Table 2). The mean adjusted change in HbA1c over time between groups is presented in Supplementary Fig. 4.

#### HbA1c in participants with pre-existing pre-diabetes

The adjusted mean HbA1c change in ITT participants with pre-diabetes at baseline in the semaglutide group (*n* = 2) was −8.0 mmol mol^−1^ (s.e. = 2.3) compared to 0.2 mmol mol (s.e. = 1.6) in the placebo group (*n* = 3). The adjusted difference in mean HbA1c change (mmol mol^−1^) between groups was −8.3 mmol mol^−1^.

#### HbA1c in participants with pre-existing T2D

The adjusted mean HbA1c change in ITT participants with T2D at baseline in the semaglutide group (*n* = 3) was −3.4 mmol mol^−1^ (s.e. = 1.8) compared to 4.0 mmol mol^−1^ (s.e. = 1.5) in the placebo group (*n* = 4). The adjusted difference in mean HbA1c change (mmol mol^−1^) between groups was −7.4 mmol mol^−1^.

#### Pharmacological agents required for the management of T2D

The number of pharmacological agents required for T2D management in participants with pre-existing T2D was assessed in the placebo (*n* = 4, 13.8%) and semaglutide groups (*n* = 3, 8.8%) in the ITT sample.

In the semaglutide group, one of three (33.3%) participants with pre-existing T2D were on one T2D medication at baseline; two participants (66.7%) were on none. At week 68, two of three (66.7%) had no change in the number of T2D agents from baseline, with the remaining participant (33.3%) having an increase in the number of T2D agents.

In the placebo group, four of four (100%) participants with pre-existing T2D were on at least one T2D medication at baseline. Two (50%) participants were on one T2D medication and two (50%) were on two T2D medications. At week 68, all four participants (100%) with pre-existing T2D had no change in the number of T2D agents from baseline.

The number of pharmacological agents required for the management of T2D at week 68 from baseline did not significantly differ between groups (*P* = 0.429).

#### Lipid profile and high-sensitivity C-reactive protein

Changes in total cholesterol, triglycerides and high-sensitivity C-reactive protein (hsCRP) are reported in Table 2. The adjusted mean total cholesterol and triglyceride change in the semaglutide group was −0.3 mmol l^−1^ (s.e. = 0.2) and −0.3 mmol l^−1^ (s.e. = 0.1), respectively. In the placebo group, the mean change in total cholesterol and triglycerides was 0.2 mmol l^−1^ (s.e. = 0.2) and 0.2 mmol l^−1^ (s.e. = 0.1), respectively. The adjusted difference in mean cholesterol and triglyceride change between groups was −0.5 mmol l^−1^ (95% CI −0.9 to −0.1, *P* = 0.014) and −0.5 mmol l^−1^ (95% CI −0.9 to −0.3, *P* < 0.001), respectively.

The adjusted mean hsCRP change in the semaglutide group was 0.4 mg l^−1^ (s.e. = 1.6) compared to 1.4 mg l^−1^ (s.e. = 1.5) in the placebo group. The adjusted difference in mean hsCRP change between groups was −1.0 mg l^−1^ (95% CI −3.9 to 2.0, *P* = 0.517).

#### Impact of Weight on Quality of Life-Lite scores

Changes in total and individual components of the Impact of Weight on Quality of Life-Lite (IWQOL-Lite) score are reported in Table 2. Baseline values of IWQOL-Lite scores are reported in Supplementary Table 5.

The adjusted difference in mean IWQOL-Lite total score between groups from was 14.3 (95% CI 6.5 to 22.1). Significant improvements in adjusted component scores, including physical function (15.7, 95% CI 7.7 to 23.7), self-esteem (16.9, 95% CI 5.5 to 28.3) and work (10.1, 95% CI 0.12 to 20.1), between groups were observed, with higher scores observed in the semaglutide group, indicating a better quality of life.

There was no statistically significant adjusted difference in sex life (12.7, 95% CI −0.7 to 26.0) and public distress (9.3, 95% CI −1.9 to 20.4) between groups.

#### Association between baseline change in GLP-1 (T0 to T30) and %WL

Median T0 (time = 0 fasted) GLP-1 levels in the placebo group were 3.0 pg ml^−1^ (interquartile range (IQR) 2.2–4.8) compared with 2.9 pg ml^−1^ (IQR 2.4–4.6) in the semaglutide group. Median T30 (time = 30 min after meal test) GLP-1 levels in the placebo group were 15.8 pg ml^−1^ (IQR 5.6–32.0) compared with 10.4 pg ml^−1^ (IQR 6.4–31.3) in the semaglutide group (Supplementary Fig. 5). The difference in change from T0 to T30 was measured. When added to the primary outcome model, meal-stimulated changes in GLP-1 at baseline (from T0 to T30) were not significantly associated with %WL at 68 weeks (*P* = 0.563).

#### AEs

Reported AEs and their frequency for the semaglutide and placebo groups are illustrated in Table 3. The risk ratio (RR) for the proportion of participants experiencing at least one AE between the semaglutide and placebo arms was 1.03 (95% CI 0.93 to 1.14; reference = placebo; *P* = 0.903). There was a statistically significant greater frequency of reporting nausea (RR 3.0 (95% CI 1.7 to 6.3); *P* < 0.001) and decreased appetite (RR 3.8 (95% CI 1.5 to 12.2); *P* = 0.009) in the semaglutide group compared with placebo.

**Table 3:** AEs in the study population (all randomized participants, *n* = 70)

	Placebo (*n* = 35)		Semaglutide 2.4 mg (*n* = 35)		Total (*n* = 70)			
	No. of events	No. of patients, *n* (%)	No. of events	No. of patients, *n* (%)	No. of events	No. of patients, *n* (%)	RR (95% CI)	*P*
Total AEs	231	33 (94.3)	310	34 (97.1)	541	67 (95.7)	1.03 (0.93 to 1.14)^a^	0.903
Gastrointestinal								
Diarrhea	17	14 (40)	29	21 (60)	46	35 (50)	1.5 (0.9 to 2.6)	0.103
Nausea	10	8 (22.9)	27	24 (68.6)	37	32 (45.7)	3.0 (1.7 to 6.3)	<0.001
Decreased appetite	4	4 (11.4)	15	15 (42.9)	19	19 (27.1)	3.8 (1.5 to 12.2)	0.009
Vomiting	7	6 (17.1)	15	10 (28.6)	22	16 (22.9)	1.7 (0.7 to 4.5)	0.264
Constipation	8	8 (22.9)	14	13 (37.1)	22	21 (30)	1.6 (0.8 to 3.7)	0.202
Abdominal pain	5	5 (14.3)	8	8 (22.9)	13	13 (18.6)	1.6 (0.6 to 4.9)	0.364
Upper abdominal pain	0	0 (0)	2	2 (5.7)	2	2 (2.9)	NA	–
Dyspepsia	5	5 (14.3)	9	9 (25.7)	14	14 (20)	1.8 (0.7 to 5.4)	0.243
Abdominal bloating	6	6 (17.1)	4	4 (11.4)	10	10 (14.3)	0.7 (0.2 to 2.1)	0.499
Gastritis	0	0 (0)	1	1 (2.9)	1	1 (1.4)	NA	–
Cardiovascular events								
Dizziness	5	5 (14.3)	13	12 (34.3)	18	17 (24.3)	2.4 (1.0 to 6.9)	0.066
Palpitations	1	1 (2.9)	3	3 (8.6)	4	4 (5.7)	3.0 (0.4 to 59.1)	0.331
Chest pain	1	1 (2.9)	1	1 (2.9)	2	2 (2.9)	1.0 (0.04 to 24.6)	1.000
Hepatobiliary								
Cholelithiasis	0	0 (0)	2	2 (5.7)	2	2 (2.9)	NA	–
Infections								
Upper respiratory tract	11	10 (28.6)	20	14 (40)	31	24 (34.3)	1.4 (0.7 to 2.8)	0.320
Nasopharyngitis	4	4 (11.4)	6	5 (14.3)	10	9 (12.9)	1.3 (0.4 to 4.7)	0.722
Sinusitis	0	0 (0)	2	2 (5.7)	2	2 (2.9)	NA	–
Cellulitis	1	1 (2.9)	0	0 (0)	1	1 (1.4)	NA	
Influenza	2	2 (5.7)	0	0 (0)	2	2 (2.9)	NA	
General								
Headache	10	10 (28.6)	16	14 (40)	26	24 (34.3)	1.4 (0.7 to 2.8)	0.320
Fatigue	10	10 (28.6)	13	12 (34.3)	23	22 (31.4)	1.2 (0.6 to 2.5)	0.513
Injection site reactions	11	9 (25.7)	10	10 (28.6)	21	19 (27.1)	1.1 (0.5 to 2.5)	0.269
Insomnia	1	1 (2.9)	4	4 (11.4)	5	5 (7.1)	4.0 (0.6 to 76.2)	0.204
Back pain	1	1 (2.9)	4	4 (11.4)	5	5 (7.1)	4.0 (0.6 to 76.2)	0.204
Arthralgia	5	4 (11.4)	2	2 (5.7)	7	6 (8.6)	0.5 (0.1 to 2.4)	0.405
Dry mouth	0	0 (0)	2	2 (5.7)	2	2 (2.9)	NA	
Renal impairment	1	1 (2.9)	2	2 (5.7)	3	3 (4.3)	2.0 (0.2 to 41.9)	0.564
Other	105	29 (82.9)	86	30 (85.7)	191	59 (84.3)	1.03 (0.84 to 1.27)	0.896

^a^RR is specifically for the number and proportion of participants experiencing at least one AE between arms, where placebo = 33 (and 145 specific incidences) and semaglutide 2.4 mg = 34 (226 specific incidences).

There were 12 serious AEs (SAEs) (11 SAEs and one SUSAR) in the trial (Table 4). Nine SAEs were in participants in the placebo group and two SAEs and one SUSAR were in the semaglutide group. Four SAEs and one SUSAR were deemed as ‘possibly related to investigational medicinal product (IMP)’; however, all four participants with SAEs were in the placebo group, with the participant with SUSAR in the semaglutide group. All participants with SAEs (including SUSAR) recovered. The SUSAR related to a de novo eating disorder (restrictive eating behavior), which led to discontinuation of the trial product (semaglutide 2.4 mg); however, it did not resolve upon discontinuation. The participant had been screened by a psychologist before MBS with no concerns noted. The participant was subsequently referred to an eating disorder service and was successfully treated. There were no treatment-related deaths in the trial. The RR for the proportion of participants experiencing at least one SAE between semaglutide and placebo arms was 0.40 (95% CI 0.06 to 1.72; reference = placebo; *P* = 0.253).

**Table 4:** SAEs in the BARI-STEP study population (all randomized participants, *n* = 70)

SAEs	No. of events	No. of patients	Causality	Outcome	Treatment allocation
Gastroesophageal reflux disease	1	1	Possibly related to IMP	Recovered	Placebo
Fractured patella	1	1	Not related to IMP	Recovered	Placebo
Rupture of right quadriceps tendon	1	1	Not related to IMP	Recovered	Placebo
Gallstones	1	1	Possibly related to IMP	Recovered	Placebo
Pancreatitis	2	1	Possibly related to IMP	Recovered	Placebo
Spontaneous bruising	1	1	Not related to IMP	Recovered	Semaglutide
Acute coronary syndrome	1	1	Not related to IMP	Recovered	Placebo
Abdominal wall abscess	1	1	Not related to IMP	Recovered	Semaglutide
Ectopic pregnancy^a^	1	1	Not related to IMP	Recovered	Placebo
Bradycardia	1	1	Not related to IMP	Recovered	Placebo
SUSAR					
Eating disorder	1	1	Possibly related to IMP	Recovered	Semaglutide

^a^Ectopic pregnancy recovered due to surgery; however, follow-up information of the outcome of the episode is unknown at the request of the participant. Crude RRs with 95% CIs were computed using log-binomial regression to compare the proportion of participants experiencing at least one SAE between treatment arms. The RR for the proportion of participants experiencing at least one SAE between semaglutide and placebo arms was 0.40 (95% CI 0.06, 1.72; reference = placebo; two-sided *z*-statistic; d.f. 68; *P* = 0.253).

#### Exploratory analysis

In the semaglutide group, %WL did not significantly differ with age, ethnicity, diabetes status, sex, type of surgery, time from surgery (months), baseline meal-stimulated GLP-1 change, postoperative %WL (from date of surgery (DOS) to randomization), maximum %WL achieved after surgery or MBS response (weight regain versus suboptimal post-surgery weight loss).

#### Sensitivity analysis

PP, repeated-measures and inverse probability weighting analyses were consistent with the primary outcome analysis (Supplementary Information). PP analysis (32 participants in the semaglutide group and 25 participants in the placebo group) showed greater percentage change in adjusted body weight in the semaglutide group compared to the placebo group (mean (s.e.), −18.5% (2.1) versus 1.10 (2.2), respectively) with an adjusted mean difference of −19.6% (95% CI −15.6 to −23.5; *P* < 0.001), which is consistent with the results for the primary analysis. Repeated-measures analysis of %WL is presented in Supplementary Table 6.

## Discussion

BARI-STEP, a randomized, placebo-controlled trial, investigated the effect of semaglutide 2.4 mg as an adjunct to lifestyle modification on %WL in patients who had previously undergone MBS with a suboptimal clinical response. Our results demonstrate that the effects of once-weekly semaglutide 2.4 mg after MBS are in line with the reported safety and tolerability profile. We provide new evidence that treatment with semaglutide 2.4 mg weekly results in clinically relevant weight reduction. The mean treatment difference between mean %WL in placebo versus semaglutide 2.4 mg was −19.1% (95% CI −23.4 to −14.8, *P* < 0.001). A recent meta-analysis^21^ also underscored the use of semaglutide 1.0–2.4 mg as a valuable adjunctive therapy in optimizing long-term outcomes for patients with a suboptimal clinical response after MBS (estimated treatment difference −15.7, 95% CI −22.1 to −9.3, *P* < 0.001).

In this patient population, %WL did not significantly differ with age, ethnicity, diabetes status, sex, time from surgery (months), type of surgery, postoperative %WL or MBS response (weight regain versus suboptimal weight loss). Previous studies suggested that patients with T2D typically experience lower %WL with GLP-1RAs, including semaglutide 2.4 mg compared with those with normoglycemia^22^. In BARI-STEP, participants with T2D on semaglutide 2.4 mg had comparable %WL to participants without T2D, despite the number of participants with T2D in this trial being low. Interestingly, although no conclusions can be drawn, the weight loss response to semaglutide 2.4 mg in the BARI-STEP trial appears to be greater than that reported in other semaglutide trials. In the STEP 1 trial^23^, the mean change in body weight from baseline to 68 weeks was −14.9%, compared with −2.4% with placebo. Similarly, the estimated treatment difference in STEP 1 was −12.4% compared with −19.1% in BARI-STEP, which was designed based on the STEP 1 trial. Moreover, among semaglutide-treated participants in the STEP 1 trial, 34.8% lost 20% or more of their body weight by week 68 compared to 47.06% in BARI-STEP, while 74.8% compared with 85.29% had weight loss of 10% or more, respectively. It is worth noting that our participants had a higher baseline BMI (mean 41.5 kg m^−^^2^) than in the STEP 1 trial (37.9 kg m^−^^2^), with some studies^24^ showing that greater weight loss is observed with a higher baseline BMI, which may explain the greater %WL observed in our trial. Moreover, in BARI-STEP, 80% of participants in the semaglutide 2.4 mg group were female, compared to 73.1% of those in the STEP 1 trial. Research suggests that females lose more weight because of semaglutide 2.4 mg compared to males^25^; thus, this may also be a contributing factor to the differences in observed %WL in BARI-STEP and STEP 1. However, in our study, biological sex had no impact on %WL.

Our previous study, BARI-OPTIMISE, selectively recruited participants with suboptimal weight loss after MBS who also had a blunted meal-stimulated GLP-1 response, based on observational study data correlating higher native postprandial GLP-1 levels to higher %WL after MBS. BARI-OPTIMISE demonstrated comparatively higher %WL to the SCALE trial; however, given the trial design, a relationship between the lower native GLP-1 levels in the trial population and the higher %WL could not be inferred. However, in this trial, weight loss response to semaglutide 2.4 mg at 68 weeks was not related to meal-stimulated endogenous GLP-1 response at baseline, suggesting that the supraphysiological effect of semaglutide probably overrides the biological mechanisms linked to a suboptimal clinical response. However, further studies are required to elucidate the underlying mechanisms and whether the effectiveness of semaglutide differs in people with a suboptimal initial weight loss response compared to those who experience weight regain. Nevertheless, our findings also suggest an increased efficacy of GLP-1RAs in a post-bariatric population, compared with people with obesity with no history of bariatric interventions.

To date, the only dose-specified, comparator, inclusive study between patients with obesity with and without a history of MBS, was conducted in 2024^26^; the study compared semaglutide at 2.4 mg weekly in 129 adults with prior bariatric surgery versus surgery-naive controls. Participants with a history of MBS had greater weight loss at 24 weeks (−9.8%) compared with those without a history of MBS (−8.8%); however, it did not reach statistical significance. A recent^27^ real-world study reported weight loss outcomes of semaglutide use in patients with or without a previous history of bariatric surgery. After 1 year of semaglutide treatment, in those with a history of metabolic surgery, %WL was significantly higher in patients who underwent RYGB (but not SG) compared with those with no MBS history. Similarly, the recent SEMALEAN study found that participants with a history of MBS exhibited greater weight loss and more pronounced reductions in body composition parameters (total fat mass) with semaglutide 2.4 mg compared with those without a history of MBS^28^.

Clinical interest in semaglutide-induced loss of lean mass is increasing, which is particularly relevant in a post-MBS population likely to experience surgery-induced loss of lean mass. However, we provide supporting evidence that the weight loss from semaglutide 2.4 mg can be predominantly attributed to loss of fat mass, as opposed to soft lean tissue mass. When calculated as a proportion relative to total body weight, participants in the placebo group experienced a reduction in soft lean tissue mass of −0.8% compared to those in the semaglutide arm who gained 5.5% of lean soft tissue mass relative to total body weight. This is in line with other studies; for example, in STEP 1 (ref. ^29^), lean body mass decreased by 9.7% from baseline. However, the proportion relative to total body mass increased by 3%. This improvement in body composition is an important therapeutic goal because increased lean soft tissue mass is associated with increased insulin sensitivity^30^, improved metabolic health and increased life expectancy^31^. However, further research with physical function measurements is warranted to identify whether muscle function and strength are affected.

Additionally, we provide important clinical evidence that 68 weeks of semaglutide 2.4 mg weekly may rival weight loss results from revisional metabolic surgery when undertaken for weight optimization after a suboptimal outcome to a primary surgical procedure, providing a minimally invasive and cost-effective alternative. There are currently no head-to-head studies comparing revisional MBS versus semaglutide 2.4 mg for post-MBS suboptimal weight loss. However, there is notable variability in %WL induced by revisional surgery, ranging from 7.8% to 37.7%, as evidenced by a recent meta-analysis^32^. Limitations of revisional surgery include invasiveness, cost, low scalability, heterogeneity and higher complication rates compared to primary MBS^33^. In this context, our findings suggest that devising treatment algorithms combining MBS and pharmacotherapy can lead to more effective and patient-centered long-term treatment strategies for severe obesity.

Across the literature, weight loss typically parallels an improvement in quality of life^34^. We demonstrate that 68 weeks of semaglutide 2.4 mg resulted in a significant improvement in self-reported quality of life (IQWOL-Lite) across domains, including physical function, self-esteem and work. This is in line with the other STEP trials, which also reported a significant improvement in IQWOL-Lite^35^.

More than 15% body weight reduction is clinically relevant, resulting in metabolic health improvements, such as improved glycemia and remission of T2D^36^. Our study supports the existing literature that semaglutide 2.4 mg ameliorates glycemic control^37,38^. At the end of 68 weeks, we report an adjusted treatment difference of −5.3 mmol mol^−1^ (−6.6 to −4, *P* < 0.001) in HbA1c. Semaglutide 2.4 mg led to significant reductions in HbA1c both in patients with (*P* = 0.011) and without (*P* < 0.001) T2D. Although participants on semaglutide 2.4 mg had a significant reduction in SBP and DBP compared with placebo, in our ITT analysis, the adjusted mean difference in SBP fell short of statistical significance (*P* = 0.059). This may be because most participants were normotensive at baseline. Nevertheless, in participants with pre-existing hypertension at baseline, there was a statistically significant reduction in SBP at 68 weeks (*P* = 0.029). Importantly, the use of semaglutide 2.4 mg for weight management has a favorable safety profile. The most frequently reported AEs are gastrointestinal in nature, including nausea, vomiting, diarrhea and constipation, reflective of the effects of GLP-1 agonists on the gastrointestinal system. However, only the frequency of reduced appetite and nausea was significantly higher in semaglutide-treated patients. All AEs were mild or moderate in severity. There were 12 SAEs (11 SAEs and one SUSAR) in the trial (Table 4). Nine SAEs were in participants in the placebo group and two SAEs and one SUSAR were in participants in the semaglutide 2.4 mg group. Four SAEs and one SUSAR were deemed as ‘possibly related to IMP’; however, of those, all four participants with SAEs were in the placebo group, with the participant with SUSAR in the semaglutide 2.4 mg group. The only case of pancreatitis reported occurred in the placebo group. Five participants in the semaglutide group reduced their dose because of AEs. Thus, semaglutide 2.4 mg is safe and well tolerated at a minimum of 1 year after MBS. Overall, the incidence and nature of AEs were consistent with the known safety and tolerability profile of semaglutide (predominantly gastrointestinal), with no new safety concerns for the post-bariatric population.

Current management paradigms for severe obesity include medical management or MBS; however, this study adds to the growing evidence of a multi-pronged combination approach addressing obesity as a long-term condition. The reverse approach to this trial may well be clinically applicable also, where GLP-1RAs are used before MBS to optimize patients for surgery, which will probably become more common in the future given the increasing availability of semaglutide 2.4 mg. While an individualized approach is necessary, further studies are required to understand how to effectively apply the combination approach and which cohort of people with obesity will benefit the most. Further research is also warranted to investigate the use of semaglutide 2.4 mg in patients who have had more than 20% weight loss since MBS yet are still living with obesity. However, this trial provides evidence for combination therapy with MBS and GLP-1RAs and reassurance about the safety and efficacy in postsurgical patients. It builds on a foundation that will enable us to devise treatment algorithms combining surgery and medicine for more severe forms of obesity.

Limitations of our study include only recruiting patients after primary MBS surgery (with a small sample size of GB patients). Our study recruited participants with a suboptimal clinical response, encompassing a suboptimal initial weight loss response and weight regain after an initial period of weight loss. It is important to note that the pathophysiology for these two presentations differs; however, our exploratory comparison showed no difference in weight loss response to semaglutide. Further studies are warranted in exploring if long-term treatment outcomes may differ. Additionally, during the 68-week treatment period, although weight loss slowed, it did not plateau, suggesting that a longer treatment period may be necessary to achieve the maximum benefits of semaglutide 2.4 mg in this patient population, which this trial did not capture. Comparatively, longer durations of semaglutide 2.4 mg, including the 104-week STEP 5 trial, show that weight loss plateaus around week 60 in the surgically naive patient population. Although, there is no clear evidence to suggest that the efficacy of semaglutide differs according to race or ethnicity, 67.1% of our participants were White; thus, combination therapy should be evaluated in a more diverse cohort. Data regarding prior use of GLP-1RAs or other weight management pharmacotherapies were not collected. Moreover, we did not conduct physical functioning tests, which is an important aspect of lean mass; however, this was because of post-COVID access restrictions to our research spaces. Because of the relatively small sample size, the ability to draw conclusions regarding the prevalence of the presentations reported in our SAEs is limited. Although the difference did not reach statistical significance, there is a trend toward a difference between %WL since surgery at baseline between groups (*P* = 0.061). At baseline, participants in the placebo group had a 3.4% greater weight loss since MBS, indicating a more favorable metabolic response to previous weight loss intervention compared with participants in the semaglutide 2.4 mg group. This potential imbalance at baseline may underestimate the treatment effect of semaglutide 2.4 mg. Lastly, even though we provided dietary and exercise counseling to all participants, energy intake and physical activity were not measured, which may be a confounding factor of interindividual variability in weight loss between groups.

In conclusion, BARI-STEP, a randomized, placebo-controlled trial investigating the use of once-weekly semaglutide 2.4 mg in a post-bariatric patient population with a suboptimal clinical response, demonstrates that semaglutide 2.4 mg in patients with a suboptimal clinical response results in substantial, clinically relevant body weight reduction, and improvements in metabolic health and quality of life. AEs in the trial were in line with the reported safety and tolerability profile of semaglutide 2.4 mg, with most reported as mild, transient and predominantly gastrointestinal-related. These findings offer the evidence base to support the use of semaglutide 2.4 mg weekly in patients with a history of MBS and the rationale for suboptimal clinical response, posing an indication for treatment. The BARI-STEP results also strengthen the evidence base for a multimodal treatment approach for severe obesity using combination therapies. Treatment algorithms incorporating multidisciplinary combination therapies with MBS and pharmacotherapy for severe obesity are now warranted.

## Methods

### Study design

BARI-STEP is a 68-week double-blind, randomized, placebo-controlled, two-arm, parallel group trial. The aim of the trial was to evaluate the therapeutic effects of semaglutide 2.4 mg in patients with a suboptimal clinical weight loss response or weight regain, which was defined as less than 20% weight loss from their DOS, in line with an internationally endorsed definition of a suboptimal treatment outcome after MBS and previous published work in this area^39^. Written informed consent was obtained and all participants and clinical study personnel were blinded. Participants were reimbursed for travel expenses; however, no additional compensation was provided.

### Randomization procedure

Participants were randomly assigned in a 1:1 ratio to receive either semaglutide 2.4 mg or placebo. Randomization was carried out by a computer-generated randomization sequence (sealed envelope) stratified according to surgical procedure type (GB versus SG), sex and T2D status. The primary outcome was %WL after 68 weeks of treatment. Clinical study personnel were blinded to the randomization procedure until after the trial had ended.

### Ethical approval

The trial was approved by the London Surrey Borders Research Ethics Committee (no. 22/LO/0045) and was conducted in accordance with the Declaration of Helsinki, the principles of good clinical practice and all applicable regulatory requirements, including the Research Governance Framework and the Medicines for Human Use (Clinical Trial) Regulations 2004 and any subsequent amendments. The trial was registered at ClinicalTrials.gov (registration: NCT05073835), the UK Medicines and Healthcare products Regulatory Agency and the European Union Drug Regulating Authority Clinical Trials (EudraCT no. 2021-004568-83). The study followed the CONSORT reporting guideline.

### Study monitoring

Independent oversight committees included the Data Monitoring Committee (DMC), the Trial Steering Committee (TSC) and the Trial Management Committee, and were held on a regular basis throughout the trial. The DMC reviewed overall safety data to determine patterns and trends of events, or to identify safety issues throughout the trial. The TSC reviewed any recommendations of the DMC and, on consideration of this information, recommended any appropriate amendment or actions for the trial as necessary. The Trial Management Committee were responsible for maintaining the oversight of the trial.

### Sample size calculation

A sample size calculation was conducted using the 24-week primary ITT analysis from our BARI-OPTIMISE trial^13^ and the results of the Davies et al.^22^ multicenter trial of semaglutide 3.0 mg ml^−1^ in adults with overweight or obesity and T2D. Assuming an s.d. for %WL of 4.0, a dropout rate of 10% and 1% critical significance level, 62 patients (31 per group) would provide at least 95% power to detect an estimated difference of 10%WL with a 95% CI no wider than 8.0 to 12.0. The recruitment target was set at 35 participants per group.

### Intervention

Seventy eligible participants were randomly assigned (1:1) to either semaglutide 2.4 mg weekly (Novo Nordisk) (*n* = 35) or placebo (saline solution) (*n* = 35), using self-administered once-weekly subcutaneous injections with identical-appearing pens. All participants received dietary and lifestyle counseling aiming for a daily 500-kcal energy deficit and were encouraged to undertake a minimum of 150 min weekly moderate-to-vigorous exercise. Participants in the intervention and placebo groups followed an identical 16-week dose escalation protocol, titrating up to a dose of 2.4 mg weekly or maximal tolerated dose.

An identical placebo containing no active ingredients was used as a comparator to evaluate the real treatment effect. A combination of in-person and remote visits took place at 1, 2, 4, 6, 8, 10, 12, 14, 16, 20, 24, 28, 32, 36, 40, 44, 48, 52, 56, 60, 64 and 68 weeks after randomization. Data including weight, AEs and concomitant medication use were collected at each visit. Participants remained in the trial for an additional 6-week wash out period with remote monitoring at weeks 70 and 74.

### Participants

BARI-STEP was conducted at University College London Hospitals (UCLH). Participants were recruited from the Bariatric Clinics at UCLH and Homerton University Hospital. Participants were enrolled between 18 November 2022 and 2 November 2023.

### Inclusion criteria

1. Adults (aged 18–65 years inclusive), ≥1 year primary GB or primary SG, with poor weight loss (weight loss of <20%) not caused by either a surgical or psychological problem.
2. Females of childbearing potential and female partners of male participants must be willing to use a highly effective method of contraception.
3. A self-reported ≤5% variation in body weight over the preceding 3 months.
4. Fluent in English and able to understand and complete questionnaires.
5. Capable to provide written informed consent and comply with the trial protocol.

### Exclusion criteria

1. Bariatric surgical procedure other than GB and SG, or revisional bariatric surgery of any type.
2. Personal history of type I diabetes or T2D mellitus treated with insulin.
3. Concomitant use of GLP-1R agonist or dipeptidyl peptidase-4 inhibitors.
4. Female who is pregnant, breastfeeding or intends to become pregnant.
5. Current participation in another clinical intervention trial.
6. History of suicidal attempt in the previous 5 years or untreated severe depression or mental health condition.
7. Symptomatic gallstone disease.
8. Uncontrolled hypertension.
9. Renal impairment measured as an estimated glomerular filtration rate less than 15 ml min 1.73 m^2^.
10. Known or suspected hypersensitivity to semaglutide.
11. Personal or family history of medullary thyroid carcinoma or multiple endocrine neoplasia syndrome type 2.
12. History of malignant neoplasms within the past 5 years.
13. Personal history of acute pancreatitis 180 days before screening or chronic pancreatitis.
14. Uncontrolled thyroid disease.
15. History of stroke, unstable angina, acute coronary syndrome or congestive heart failure New York Heart Association classes III and IV within the preceding 12 months.
16. Untreated therapeutically relevant arrhythmias.
17. Diabetic gastroparesis.
18. Concomitant use of medications that cause weight gain or weight loss.
19. Known or suspected abuse of alcohol or recreational drugs.
20. Severe hepatic impairment diagnosed using liver function blood tests and clinical evaluation.
21. Any additional factor, which in the investigator’s opinion might jeopardize the participant’s safety or compliance with the trial protocol.

### Procedures

Before commencing treatment, baseline assessments were performed, including sociodemographic data collection and medical history. Participant sociodemographic data were self-reported in response to questions asked by the investigator. Biological sex was determined from medical records. Meal-stimulated plasma GLP-1 levels in response to a liquid meal test (187.5 ml, 450 kcal, 17.5 g fat, 54.0 g carbohydrate, 19.1 g protein, Abbott Ensure) were measured at baseline, fasted (0 min = T0) and 30 min after starting the liquid meal (T30).

Gut hormone (GLP-1) and inflammatory indices were measured in human plasma samples using sandwich enzyme linked immunosorbent assays (ELISAs). Each ELISA was performed according to specific instructions from the manufacturer, including precautions and safety warnings for particular reagents (Merck Active GLP-1 ELISA Kit; accuracy: 86.9%; standard curve range: 2–100 pM)

Weight and body composition measures were assessed at six time points (baseline and weeks 6, 14, 32, 52 and 68) using bioelectrical impedance analysis (DC-430MA S, Tanita) to measure weight, lean soft tissue mass and fat mass. Participants were given consistent instructions for hydration status at visits to enhance the accuracy of bioelectrical impedance analysis; all participants were advised consistently to avoid intense exercise, alcohol and caffeine before their study visit. At the six in-person visits, metabolic secondary outcomes were measured, including HbA1c, blood pressure (systolic and diastolic), HR, total cholesterol, triglycerides, hsCRP, HbA1c in participants with pre-existing pre-diabetes and T2D, blood pressure in participants with pre-existing hypertension and changes in T2D and hypertension medication used in participants with T2D and hypertension, respectively. Health-related quality of life was also assessed at the in-person visits using the IWQOL-Lite questionnaire^40^.

### Outcomes and objectives

The primary objective was to compare the efficacy of 68 weeks of subcutaneous semaglutide 2.4 mg weekly versus placebo administration, as an adjunct to diet and exercise, on %WL in participants with less than 20% weight loss after primary GB or SG at the end of 68 weeks of treatment. The prespecified primary outcome was the difference in mean percentage body weight change between patients randomized to semaglutide versus placebo at 68 weeks.

Prespecified secondary outcomes included change in body weight (kg) from baseline and the proportion of participants who, after 68 weeks, achieved a body weight reduction 10%, 15% and 20% or more. Non-prespecified outcomes included fat mass, lean soft tissue mass, lean soft tissue mass (%), BMI and HR. Metabolic secondary outcomes included HbA1c, blood pressure (systolic and diastolic), HR, total cholesterol, triglycerides, hsCRP, HbA1c in participants with pre-existing pre-diabetes and T2D, blood pressure in participants with pre-existing hypertension and changes in T2D, and hypertension medication used in participants with T2D and hypertension, respectively. Health-related quality of life was also assessed at the in-person visits using the IWQOL-Lite questionnaire^40^. Health economic analyses, inflammatory markers and other questionnaire data will be reported separately.

### Statistical analysis

Normally distributed variables were reported using means and standard deviations. Non-normally distributed variables were reported using medians and IQRs. Categorical variables were reported using frequencies and percentages and analyzed using chi-squared tests where appropriate. Values are presented according to treatment allocation.

The primary outcome was analyzed using a linear regression model, adjusted for baseline weight, type of surgery, sex and diabetes status, according to the ITT principle. Model plots are reported in Supplementary Fig. 6.

Secondary outcomes were analyzed on an ITT basis using linear regression, adjusted for baseline weight, type of surgery, sex, diabetes status and the baseline value of the corresponding outcome. Risk differences with 95% CIs and chi-squared tests were used to compare proportions of categorical change in %WL between semaglutide and placebo.

A Fisher’s exact test was used to compare changes in the number of pharmacological agents required for the management of T2D in participants with pre-existing T2D from baseline to week 68 between arms, and to analyze changes in the number of pharmacological agents required for the management of hypertension in participants with pre-existing hypertension from baseline to week 68 between arms.

Crude RRs with 95% CIs were computed using log-binomial regression to compare the proportion of participants experiencing at least one AE or SAE between treatment arms. In cases of failed convergence with log-binomial regression (for total AE and other AE), a modified Poisson regression was used to calculate RRs with 95% CIs from robust standard errors estimated using the sandwich estimator^41^. RRs were not reported for AEs with zero events in either arm. Repeated-measure analyses of the primary and secondary outcomes at the six assessment time points were conducted with mixed-effects models using all available data, with fixed effects for treatment, visit, baseline weight, type of surgery, sex and diabetes status (and for secondary outcomes, the baseline value for the secondary outcome), an interaction term between treatment and visit, and a random intercept for participant. The normality assumptions of models were visually checked where appropriate; no transformations or nonparametric methods were used. Secondary outcomes were considered exploratory and estimates of the intervention effect for the secondary outcomes are reported with 95% CIs. As secondary outcomes were exploratory in nature, reported significance values were not adjusted for multiple comparisons. Any apparent significance of these results should be confirmed in future research.

Meal-stimulated change in GLP-1 at baseline was added as a covariate to the primary outcome model in a prespecified secondary analysis, to assess the relationship with %WL at 68 weeks.

Changes in HbA1c in participants with pre-existing pre-diabetes between treatment arms, and in participants with pre-existing T2D between treatment arms, was assessed with the inclusion of an interaction term between diabetes status and treatment allocation in the linear regression model for HbA1c and calculated from estimated marginal means. For the pre-existing pre-diabetes analysis, the diabetes covariate was grouped as non-diabetic, prediabetic and diabetic.

### Exploratory analysis

Non-prespecified exploratory analyses included assessing the impact of baseline diabetes status on %WL induced by semaglutide at 68 weeks. In the semaglutide arm only, other non-prespecified baseline variables (age, ethnicity, diabetes status, sex, type of surgery, time since surgery (months), meal-stimulated change in GLP-1 at baseline, postoperative %WL (from DOS to randomization)) and maximum %WL achieved after surgery or MBS response (weight regain versus suboptimal post-surgery weight loss) were added to the primary outcome linear regression model for the semaglutide arm only (therefore omitting the treatment allocation covariate) to assess their relationship with %WL induced by semaglutide at 68 weeks.

### Sensitivity analysis

Unadjusted changes in primary and secondary outcomes at 68 weeks were computed for the ITT samples and analyzed using two-sample *t*-tests.

In the sensitivity analyses, the primary outcome analysis was repeated for the PP sample, defined as participants who followed the study protocol with full adherence, attending all study visits with no substantial interruptions in treatment as monitored from the drug dose diary. For the ITT sample, all randomized participants were used for the analyses except for those who withdrew or were lost to follow-up. Safety reporting (that is, AEs and SAEs) and safety analyses included all randomized participants (*n* = 70).

To account for missingness in the primary outcome analysis, inverse probability weighting was used. Probabilities of the primary outcome data being observed at week 68 were estimated using treatment allocation, age and baseline weight as model predictors. Stabilized weights were then used to reweight the remaining ITT sample.

A significance level of 0.05 was used for all hypothesis testing. Analyses were conducted in R (v.2024.12.1+563).

### Reporting summary

Further information on research design is available in the Nature Portfolio Reporting Summary linked to this article.

## Online content

Any methods, additional references, Nature Portfolio reporting summaries, source data, extended data, supplementary information, acknowledgements, peer review information; details of author contributions and competing interests; and statements of data and code availability are available at 10.1038/s41591-026-04416-4.

## Supplementary information

Supplementary Information Supplementary Figs. 1–6 and Tables 1–6.

Reporting Summary

## Data Availability

Individual participant data collected during the trial, after deidentification, the study protocol, statistical analysis plan and the patient information sheet (consent form) can be shared on request. Study data can be requested after publication through the corresponding author. An initial response to the request will be provided within 2 weeks; if additional approvals are required, the final response time may take up to 6 months. Depending on the nature of the request, in line with ethics approval, the study protocol and participant consent form, further approvals may be required from the Joint Research Office (UCL) or NHS Research Ethics Committee.

## Code availability

Code for the analysis is publicly available and available without restriction via GitHub at https://github.com/SamuelJDicken/BARI-STEP
