# Supplementary material for: Semaglutide versus placebo in individuals with poor weight loss after bariatric surgery: a double-blinded, randomized, placebo-controlled trial
Source: Nat Med. 2026 May 22;32(7):2662–72. doi: 10.1038/s41591-026-04416-4 (PMC13375533; doi:10.1038/s41591-026-04416-4)
Supplement: Supplementary file 1 — Supplementary Figs. 1–6 and Tables 1–6. [file 41591_2026_4416_MOESM1_ESM.pdf]

nature medicine

Supplementary information https://doi.org/10.1038/s41591-026-04416-4

Semaglutide versus placebo in individuals with poor weight loss after bariatric surgery: a double-blinded, randomized, placebo-controlled trial

In the format provided by the authors and unedited

BARI-STEP Supplementary Information

Supplementary Content

Table 1: Baseline characteristics of participants included in the primary outcome analysis (ITT) 2

Figure 1: Waterfall plot of unadjusted %WL of ITT sample at V24 (68 weeks) 4

Table 2: Categorical weight loss achieved at 68 weeks between Semaglutide 2.4mg versus placebo 5

Figure 2: Categorical weight loss achieved at 68 weeks between Semaglutide 2.4mg versus placebo 6

Table 3: Repeated measures analysis of secondary outcomes 7

Table 4: Changes in secondary outcomes at 68 weeks in the ITT population (unadjusted) 9

Figure 3: Adjusted mean change of Systolic Blood Pressure (a) and Diastolic Blood Pressure (b) over time 10

Figure 4: Mean adjusted change in HbA1C (mmol/mol) between placebo and semaglutide 2.4mg group 11

Table 5: Baseline values for IWQOL-Lite scores 12

Figure 5: Median (and inter-quartile range (IQR)) baseline GLP-1 (pg/mL) values at T0 (0 minutes fasted) and T30 (30 minutes after meal test) in placebo versus semaglutide 2.4mg participants. 13

Table 6: Repeated measures analysis of primary outcome 14

Figure 6: Primary outcome analysis linear regression model plots 15

Sensitivity analyses 16

Table 1: Baseline characteristics of participants included in the primary outcome analysis (ITT)

	Placebo (n=29)	Semaglutide 2.4mg (n=34)	Overall trial (n=63)
**Age, y**	49.4 (9.8)	47.4 (10.1)	48.3 (9.9)
**Sex, n (%)**			
Female	22 (75.9)	29 (85.3)	51 (81.0)
Male	7 (24.1)	5 (14.7)	12 (19.0)
**Weight (kg)**	113.2 (20.4)	118.6 (23.7)	116.1 (22.2)
**Body Mass Index (BMI)**	40.3 (5.9)	42.9 (6.8)	41.7 (6.5)
**BMI, kg/m^2, n (%)**			
**<35**	4 (13.8)	2 (5.9)	6 (9.5)
**≥35 to <40**	11 (37.9)	13 (38.2)	24 (38.1)
**≥40**	14 (48.3)	19 (55.9)	33 (52.4)
**%WL since DOS**	8.9 (6.7)	6.1 (8.2)	7.4 (7.6)
**Ethnicity, n (%)**			
Asian	0 (0.0)	2 (5.9)	2 (3.2)
Black	8 (27.6)	6 (17.6)	14 (22.2)
White	17 (58.6)	25 (73.5)	42 (66.7)
Mixed/Other	4 (13.8)	1 (2.9)	5 (7.9)
**Type of Surgery, n (%)**			
SG	22 (75.9)	26 (76.5)	48 (76.2)
GB	7 (24.1)	8 (23.5)	15 (23.8)
**Time since surgery (months)**	81.7 (36.1)	88.4 (51.3)	85.3 (44.7)
**Diabetes Status, n (%)**			
Type 2 diabetes	4 (13.8)	3 (8.8)	7 (11.1)
Normoglycaemia/pre-diabetes	25 (86.2)	31 (91.2)	56 (88.9)
**HbA1c (mmol/mol)**	39.5 (3.9)	37.3 (5.5)*	38.4 (4.9)
**Change in meal-stimulated GLP-1 (pg/ml)**	12.8 (3.9, 32.1)**	6.4 (2.7, 29.4)*	8.5 (3.1, 30.1)

Abbreviations: SG, sleeve gastrectomy. GB, Gastric Bypass. GLP-1, Glucagon-Like Peptide 1. HbA1C, haemoglobin A1C. Gastric bypass included standardised Roux-and-Y Gastric Bypass and One-Anastomosis Gastric Bypass. *N = 33 participants, **N = 28 participants

Figure 1: Waterfall plot of unadjusted %WL of ITT sample at V24 (68 weeks)

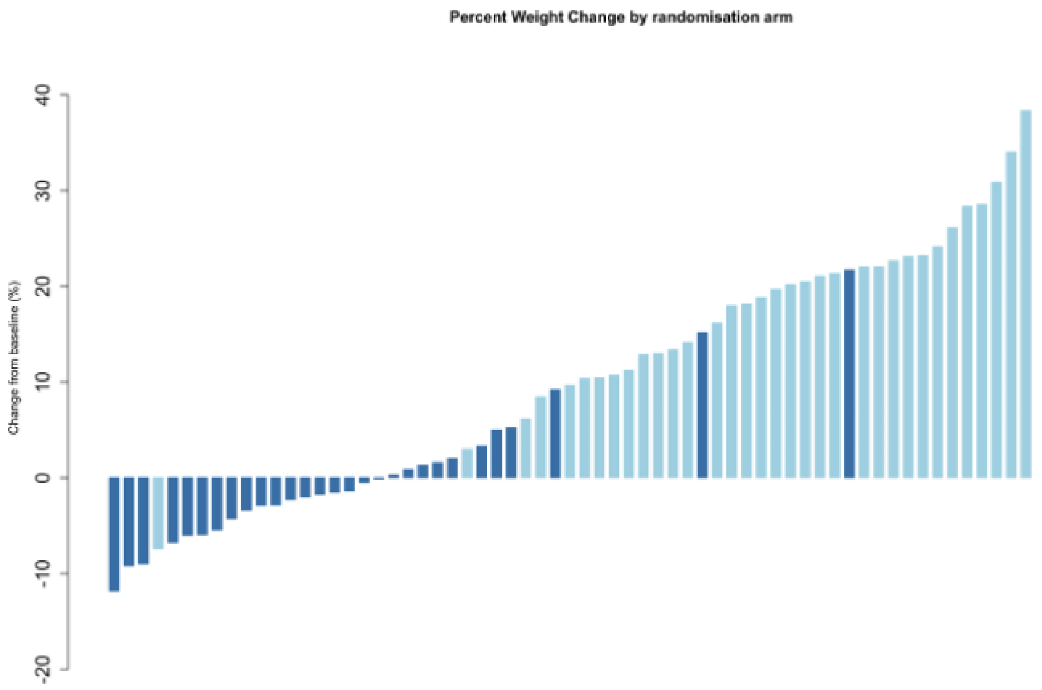

N = 63 participants, intention-to-treat sample. Dark blue: placebo; Light blue: Semaglutide 2.4mg. No statistical comparisons conducted.

Table 2: Categorical weight loss achieved at 68 weeks between Semaglutide 2.4mg versus placebo

ITT %WL Categorical	Placebo N (%)	Semaglutide 2.4mg N (%)	p value*	Risk difference %, (95%CI)
<10%	27 (93.1)	5 (14.7)	<0.001	-78.4 (-93.5, -63.3)
≥10%	2 (6.9)	29 (85.3)	<0.001	78.4 (63.3, 93.5)
≥15%	2 (6.9)	21 (61.8)	<0.001	54.9 (36.1, 73.6)
≥20%	1 (3.4)	16 (47.1)	<0.001	43.6 (25.6, 61.7)

N = 63 participants. *from Chi-square test **Binary regression adjusted for type of surgery, diabetes status, sex and baseline weight (kg). Abbreviations: ITT, Intention-to-treat. %WL, % weight loss.

Figure 2: Categorical weight loss achieved at 68 weeks between Semaglutide 2.4mg versus placebo

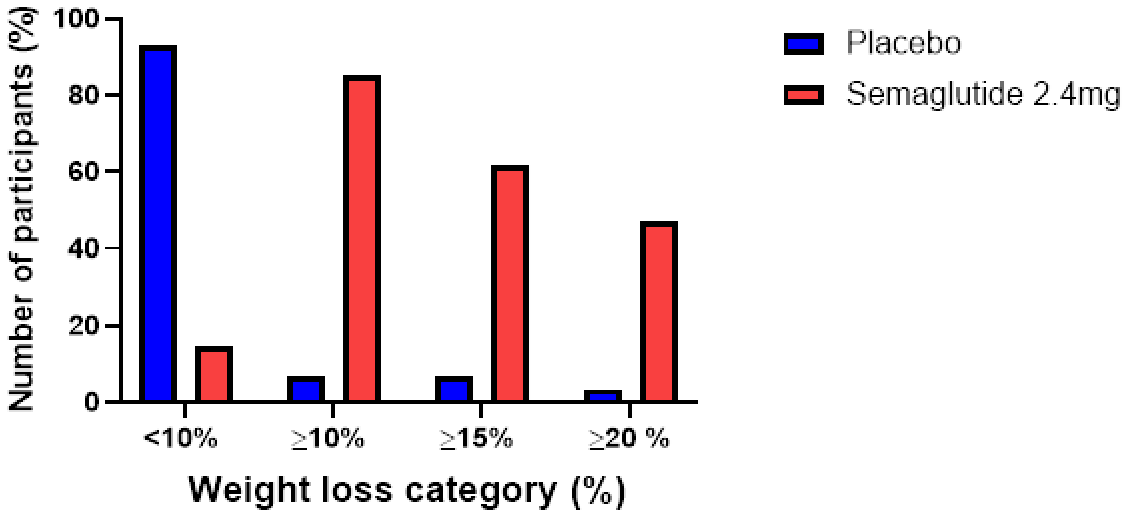

N = 63 participants, intention-to-treat sample. χ^2 test were used to compare proportions of categorical change in percentage weight loss between Semaglutide and placebo. <10%, P < 0.001; ≥10%, P < 0.001; ≥15%, P < 0.001; ≥15%, P < 0.001.

Table 3: Repeated measures analysis of secondary outcomes

All available data at each follow up (n=70)	Placebo		Semaglutide		Adjusted mean difference* (Semaglutide minus Placebo) (95% CI)	p value
	n	Mean (SE)	n	Mean (SE)		
HbA1c						
Week 6	35	0.65 (0.59)	34	-1.15 (0.57)	-1.79 (-2.87, -0.71)	0.001
14	31	1.18 (0.60)	33	-2.44 (0.57)	-3.62 (-4.73, -2.51)	<0.001
32	30	0.63 (0.60)	33	-4.14 (0.57)	-4.78 (-5.89, -3.66)	<0.001
52	28	1.03 (0.61)	31	-4.13 (0.58)	-5.15 (-6.29, -4.01)	<0.001
68	28	1.11 (0.61)	33	-3.93 (0.57)	-5.04 (-6.17, -3.91)	<0.001
SBP						
Week 6	34	4.39 (3.05)	35	1.00 (3.13)	-3.39 (-9.94, 3.16)	0.309
14	31	4.60 (3.08)	35	0.03 (3.13)	-4.58 (-11.26, 2.10)	0.178
32	30	5.01 (3.10)	34	-2.17 (3.14)	-7.18 (-13.94, -0.43)	0.037
52	28	2.75 (3.14)	33	-0.13 (3.16)	-2.88 (-9.77, 4.02)	0.412
68	28	2.99 (3.14)	34	-1.73 (3.14)	-4.72 (-11.57, 2.13)	0.176
DBP						
Week 6	34	3.44 (2.07)	35	3.32 (2.14)	-0.12 (-4.52, 4.29)	0.959
14	31	3.94 (2.09)	35	3.15 (2.14)	-0.79 (-5.27, 3.70)	0.729
32	30	4.89 (2.10)	34	4.02 (2.15)	-0.86 (-5.40, 3.67)	0.707
52	28	7.52 (2.16)	33	5.17 (2.16)	-2.35 (-6.97, 2.27)	0.316
68	28	6.13 (2.12)	34	2.03 (2.15)	-4.11 (-8.70, 0.48)	0.079
IWQoL-Lite Total						
Week 6	33	5.06 (3.24)	31	5.32 (3.33)	0.26 (-6.42, 6.94)	0.939
14	31	9.40 (3.25)	32	11.51 (3.32)	2.11 (-4.61, 8.83)	0.536
32	29	7.44 (3.27)	33	16.33 (3.30)	8.89 (2.12, 15.65)	0.010
52	28	6.64 (3.28)	31	15.48 (3.33)	8.84 (2.00, 15.69)	0.012
68	27	7.03 (3.29)	33	21.27 (3.30)	14.24 (7.42, 21.07)	<0.001

N = 70 participants, using all available data. * Estimated from repeated measures mixed effects model adjusting for baseline weight, type of surgery, sex, diabetes status, baseline value of the dependent variable and interaction between treatment and visit. Abbreviations: HbA1C, haemoglobin A1C. SBP, systolic blood pressure. DBP, diastolic blood pressure. IWQOL-Lite, Impact of Weight on Quality of Life-Lite questionnaire.

Table 4: Changes in secondary outcomes at 68 weeks in the ITT population (unadjusted)

**Outcome** (ITT, N = 63 participants)	**Placebo**		**Semaglutide 2.4mg**		**Unadjusted mean difference***	**p value***
	**No.**	**Mean (SD)**	**No.**	**Mean (SD)**		
**Total Body Weight (kg)**	29	0.5 (8.0)	34	-20.7 (10.2)	-21.2 (-25.9, -16.5)	<0.001
**Body Mass Index (BMI)**	28	0.4 (2.9)	33	-7.4 (3.8)	-7.9 (–9.6, -6.1)	<0.001
**Fat Mass (kg)**	28	4.1 (9.4)	33	-13.4 (9.2)	-17.4 (-22.2, -12.7)	<0.001
**Lean Soft Tissue Mass (%)**	28	-2.7 (5.7)	33	3.9 (5.9)	6.6 (3.6, 9.6)	<0.001
**Lean Soft Tissue Mass (kg)**	28	-2.9 (7.0)	33	-6.9 (5.0)	-4.1 (-7.1, -1.0)	0.010
**HbA1c (mmol/mol)**	28	0.04 (2.5)	33	-4.4 (3.4)	-4.4 (-6.0, -2.9)	<0.001
**Heart rate (bpm)**	28	-3.9 (10.1)	34	-5.5 (13.7)	-1.5 (-7.8, 4.7)	0.622
**SBP (mmHg)**	28	1.0 (12.8)	34	-5.1 (10.4)	-6.2 (-12.0, -0.2)	0.041
**DBP (mmHg)**	28	5.4 (12.4)	34	-0.2 (8.8)	-5.6 (-11.0, -0.2)	0.042
**Total Cholesterol (mmol/L)**	28	-0.7 (4.5)	31	-1.2 (7.0)	-0.5 (-3.5, 2.6)	0.764
**Triglycerides (mmol/L)**	28	-0.004 (0.9)	33	-0.4 (0.6)	-0.42 (-0.81, -0.03)	0.030
**HsCRP mg/L**	28	0.1 (0.6)	33	-0.4 (0.6)	-0.5 (-0.8, -0.2)	<0.001
**IWQOL-Lite**						
**Total**	27	6.7 (13.4)	33	20.0 (16.2)	13.4 (5.6, 21.2)	0.001
**Physical function**	27	6.1 (16.9)	34	19.9 (14.7)	13.9 (5.8, 22.0)	0.001
**Self-esteem**	27	10.8 (15.9)	34	26.1 (25.8)	15.3 (4.5, 26.1)	0.006
**Sex life**	22	1.7 (28.3)	31	13.4 (25.6)	11.7 (3.2, 26.7)	0.121
**Public distress**	27	9.6 (22.1)	34	22.9 (24.3)	13.3 (1.3, 25.4)	0.031
**Work**	27	4.9 (20.8)	32	14.3 (27.3)	9.4 (-3.5, 22.2)	0.148

Abbreviations: ITT, intention-to-treat. SE, standard error. HbA1C, haemoglobin A1C. SBP, systolic blood pressure. DBP, diastolic blood pressure. IWQOL-Lite, Impact of Weight on Quality of Life-Lite questionnaire. BPM, beats per minute. HsCRP, High-sensitivity C-reactive protein

Figure 3: Adjusted mean change of Systolic Blood Pressure (a) and Diastolic Blood Pressure (b) over time

A

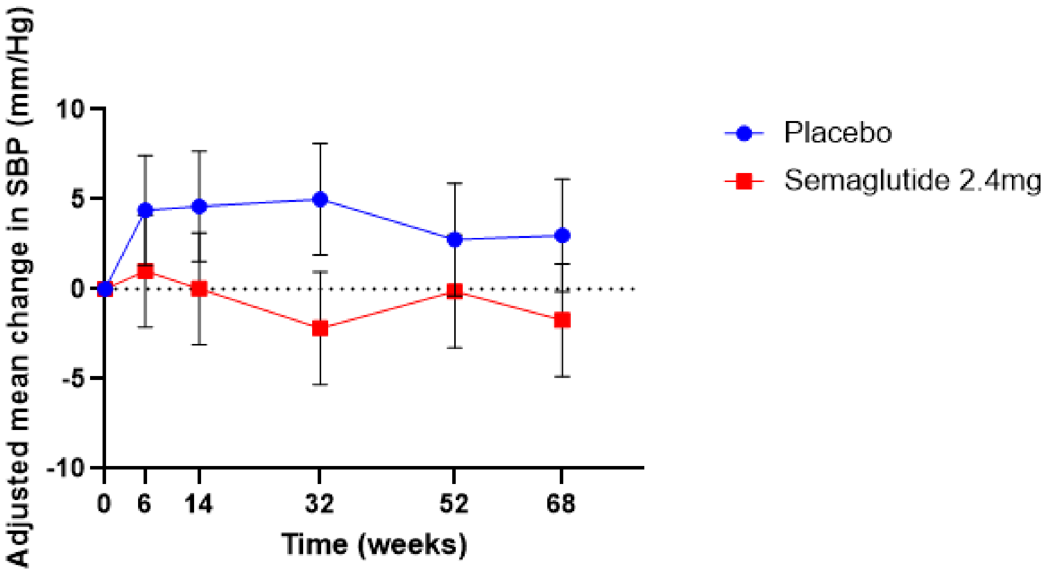

Adjusted mean change in SBP (mm/Hg) with standard error (SE) bars over time estimated from repeated measures mixed effects model adjusting for baseline SBP, type of surgery, sex, diabetes status, and interaction between treatment and visit using all available data. Two-sided t statistic with 95% confidence intervals; Week 6: N = 69; degrees of freedom: 188; P = 0.309, Week 14: N = 66; degrees of freedom: 193, P = 0.178, Week 32: N = 64; degrees of freedom: 197, P = 0.037, Week 52: N = 61; degrees of freedom: 205, P = 0.412, Week 68: N = 62; degrees of freedom: 203, P = 0.176, not adjusted for multiple comparisons.

b

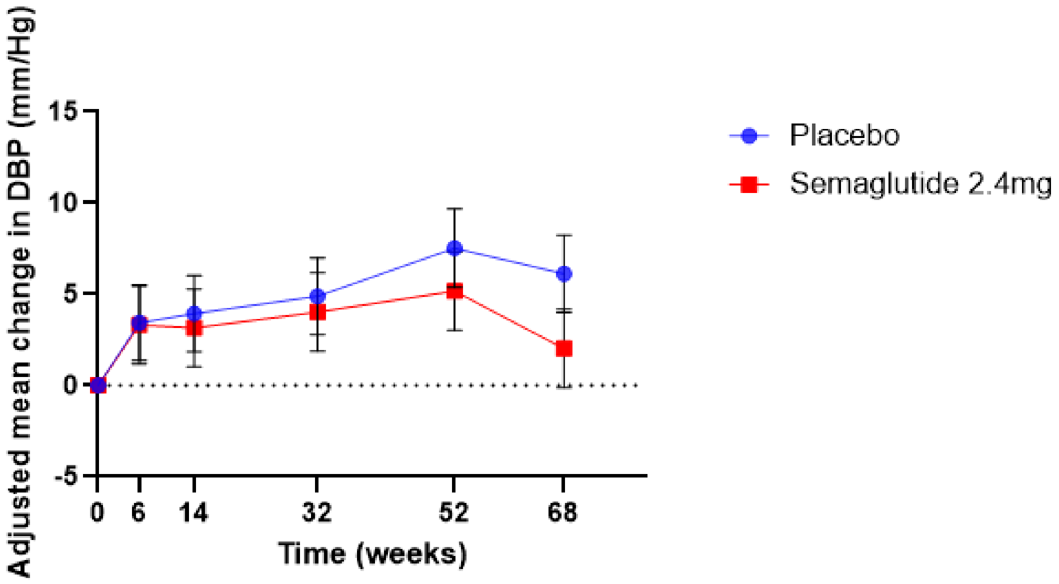

Adjusted mean change in DBP (mm/Hg) including standard error (SE) bars over time estimated from repeated measures mixed effects model adjusting for baseline DBP, type of surgery, sex, diabetes status, and interaction between treatment and visit using all available data. Two-sided t statistic with 95% confidence intervals; Week 6: N = 69; degrees of freedom: 171; P = 0.959, Week 14: N = 66; degrees of freedom: 176, P = 0.729, Week 32: N = 64; degrees of freedom: 180, P = 0.707, Week 52: N = 61; degrees of freedom: 188, P = 0.316, Week 68: N = 62; degrees of freedom: 186, P = 0.079, not adjusted for multiple comparisons.

Figure 4: Mean adjusted change in HbA1C (mmol/mol) between placebo and semaglutide 2.4mg group

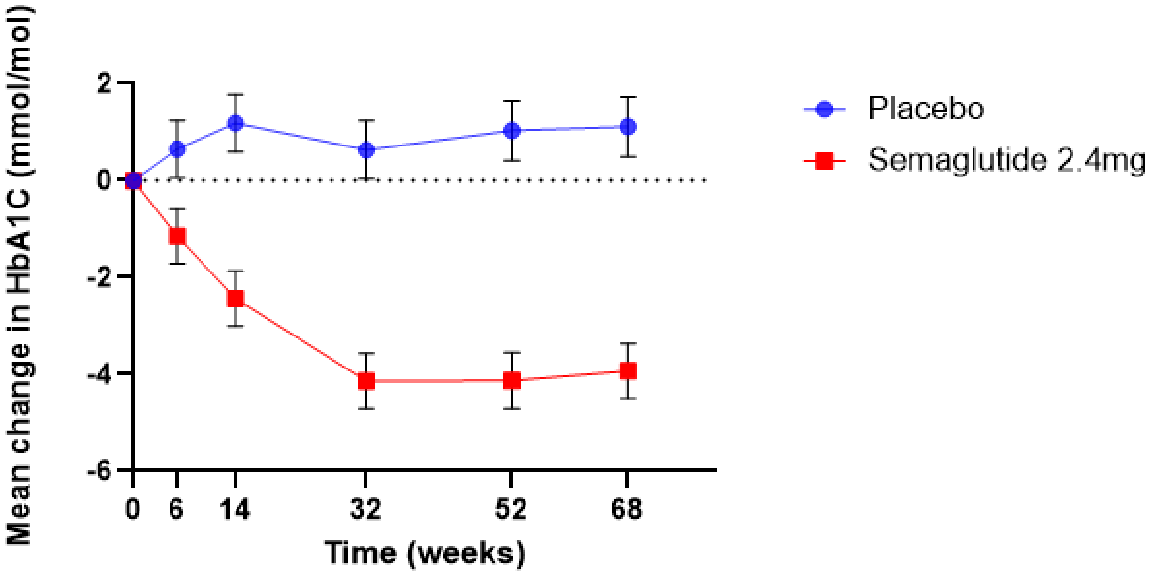

Adjusted mean change in HbA1C (mmol/mol) including standard error (SE) bars over time estimated from repeated measures mixed effects model adjusting for baseline HbA1C, type of surgery, sex, diabetes status, and interaction between treatment and visit using all available data. Two-sided t statistic with 95% confidence intervals; Week 6: N = 69; degrees of freedom: 154; P = 0.001, Week 14: N = 66; degrees of freedom: 163, P < 0.001, Week 32: N = 64; degrees of freedom: 165, P < 0.001, Week 52: N = 61; degrees of freedom: 175, P < 0.001, Week 68: N = 62; degrees of freedom: 170, P < 0.001, not adjusted for multiple comparisons.

Table 5: Baseline values for IWQOL-Lite scores

Group allocation	Placebo (n=35)	Semaglutide (n=35)
IWQOL-Lite total score (Semaglutide: 34)	50.6 (23.0)	46.7 (20.6)
Physical function	48.9 (24.2)	47.5 (25.9)
Self-esteem	35.7 (26.7)	33.2 (25.5)
Sex life (Placebo: 33, Semaglutide: 32)	59.3 (32.7)	57.2 (32.3)
Public distress	57.1 (30.0)	45.0 (27.3)
Work (Semaglutide: 33)	65.7 (30.1)	64.4 (26.4)

Higher scores indicate better quality of life. Abbreviations: IWQOL-Lite, Impact of Weight on Quality of Life-Lite questionnaire.

Figure 5: Median (and inter-quartile range (IQR)) baseline GLP-1 (pg/mL) values at T0 (0 minutes fasted) and T30 (30 minutes after meal test) in placebo versus semaglutide 2.4mg participants.

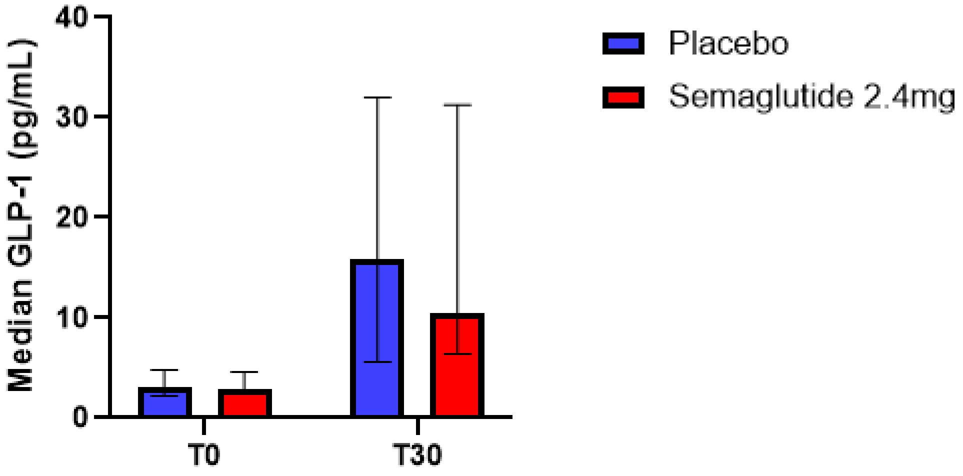

No statistical comparisons conducted.

Table 6: Repeated measures analysis of primary outcome

All available data at each follow-up (n=70)	Placebo		Semaglutide		Adjusted mean difference* (Semaglutide minus Placebo) (95% CI)	p value
	n	Mean (SE)	n	Mean (SE)		
Weight loss (%)						
Week 6	35	0.40 (1.31)	35	-3.22 (1.34)	-3.62 (-6.29, -0.95)	0.008
14	31	-0.003 (1.33)	35	-6.77 (1.34)	-6.77 (-9.49, -4.05)	<0.001
32	30	0.71 (1.33)	34	-12.96 (1.34)	-13.67 (-16.42, -10.92)	<0.001
52	28	1.27 (1.34)	33	-16.87 (1.35)	-18.14 (-20.93, -15.35)	<0.001
68	29	0.96 (1.34)	34	-18.05 (1.34)	-19.00 (-21.77, -16.24)	<0.001

* Estimated from repeated measures mixed effects model adjusting for baseline weight, sex, type of surgery and diabetes status, with interaction between treatment arm and visit.

Figure 6: Primary outcome analysis linear regression model plots

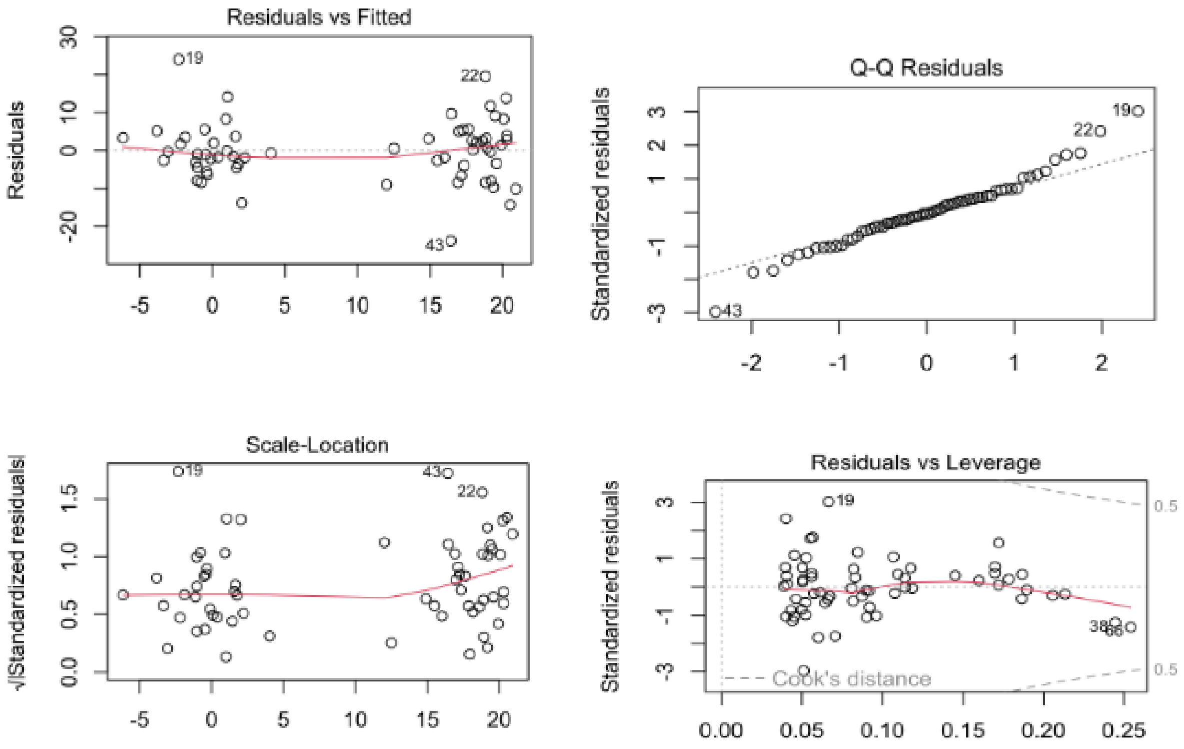

N = 63 participants, intention-to-treat sample.

Sensitivity analyses

Inverse probability weighting (IPW) was used to re-weight the remaining sample. IPW analysis was consistent with the main analysis. The adjusted mean difference (Semaglutide – Placebo) of %WL was –19.3 (SE: 2.2, $p < 0.001$, IPW [N=63]).

Docusign Envelope ID: F60CEFFC-8A22-436F-AE83-0299BDD1E22D

TITLE PAGE

FULL/LONG TITLE OF THE TRIAL

A double-blinded, randomised, placebo-controlled trial of semaglutide 2.4 mg in patients with poor weight-loss following bariatric surgery.

SHORT TRIAL TITLE / ACRONYM

BARI-STEP

PROTOCOL VERSION NUMBER AND DATE

Version 5.0, 06-09-2024

Docusign Envelope ID: F60CEFFC-8A22-436F-AE83-0299BDD1E22D

RESEARCH REFERENCE NUMBERS

**IRAS Number:**	299128
**Clinical trials.gov Number:**	NCT05073835
**EudraCT Number:**	2021-004568-83
**ISRCTN Number / Other registries: Universal Clinical Trial Number**	U1111-1263-5209
**SPONSOR:**	University College London (UCL)
**SPONSOR Number:**	142522
**FUNDER:**	NIHR, UCLH NIHR Biomedical Research Centre and Sir Jules Thorne Charitable Trust
**FUNDERS Number:**	N/A

Docusign Envelope ID: F60CEFFC-8A22-436F-AE83-0299BDD1E22D

SIGNATURE PAGE

The undersigned confirm that the following protocol has been agreed and accepted and that the Chief Investigator agrees to conduct the trial in compliance with the approved protocol and will adhere to the principles outlined in the Medicines for Human Use (Clinical Trials) Regulations 2004 (SI 2004/1031), amended regulations (SI 2006/1928) and any subsequent amendments of the clinical trial regulations, the UK Policy Framework for Health and Social Care Research 3rd edition 2017 (as amended), GCP guidelines, the UK Data Protection Act (2018), the Sponsor's (and any other relevant) SOPs, and other regulatory requirements as amended.

I agree to ensure that the confidential information contained in this document will not be used for any other purpose other than the evaluation or conduct of the clinical investigation without the prior written consent of the Sponsor.

I also confirm that I will make the findings of the trial publicly available through publication or other dissemination tools without any unnecessary delay and that an honest accurate and transparent account of the trial will be given; and that any discrepancies and serious breaches of GCP from the trial as planned in this protocol will be explained.

Chief Investigator: Dr Janine Makaronidis

Signature and Date: DocuSigned by: Janine Makaronidis EBAAFFBE1FF5437...

30 October 2024 | 10:44 GMT

For and on behalf of the Trial Sponsor:

Signature and Date: DocuSigned by: Rajinder Sidhu D225A0063221469... 30 October 2024 | 10:47 GMT

Name (please print):

Dr. Rajinder Sidhu

Position: Associate Director Research Governance and Ops

PROTOCOL VERSION HISTORY

**Version Number**	**Date**	**Protocol Update Finalised By** (insert name of person)	**Reasons for Update**
1.0	10.12.2021	Rachel Batterham	N/A
2.0	24.01.2022	Rachel Batterham	MHRA requests for approval
2.1	09.02.2022	Rachel Batterham	Drug Supplier requests – submitted as part of initial REC review
2.2	30.03.2022	Rachel Batterham	Drug Supplier requests – amendment to 'not-in-use' storage conditions and safety reporting
2.3	21.09.2022	Rachel Batterham	Update to trial team contact details.<br>Typographical updates to Section 9: Pharmacovigilance.<br>Removal of 15min blood test in section 7.6.
3.0	11.04.2023	Janine Makaronidis	Change in CI from Prof Rachel Batterham to Dr Janine Makaronidis<br>Update to trial contacts.
3.1	24.10.2023	Chloe Firman	Removal of 'Roux-en-Y' and changed to 'gastric bypass' throughout the protocol as the surgical technique for gastric bypasses have evolved
4.0	20.02.2024	Andrew Tunstell	Change in Sponsor contact from Samim Patel to Andrew Tunstell.
4.1	05.06.2024	Alanna Brown	Update to trial team contact details.
5.0	06.09.2024	Chloe Firman	Update to include the use of semaglutide 3.2 mg/ml from 05 Nov 2024 when the current stock of semaglutide 3.0 mg/ml is depleted. The trial drug manufacturer has informed us that the semaglutide 3.0 mg/ml product is no longer being manufactured for release in the United Kingdom and therefore an alternative concentration will be provided. References throughout the protocol have been amended to 'semaglutide 3.0 mg/ml or 3.2 mg/ml'.<br>Section 8.1 and 8.5 have been updated to reflect the change in dose dial.

			Section 8.11 updated to refer to new EudraCT reference number.<br>Update to PL number in section 8.2.<br>Section 7.12 has been updated to clarify the end of trial definition.

KEY TRIAL CONTACTS

**Chief Investigator**	Dr Janine Makaronidis<br>Centre for Obesity Research<br>Division of Medicine<br>Rayne Building<br>5 University Street<br>London WC1E 6JF<br>e-mail: j.makaronidis@ucl.ac.uk
**Sponsor**	Andrew Tunstell<br>Sponsor Regulatory Advisor<br>Joint Research Office, UCL<br>4th Floor West,<br>250 Euston Road<br>London NW1 2PG<br>Email: CTIMPS@ucl.ac.uk
**Clinical Trials Unit / Contract Research Organisation**	N/A
**Statistician**	David Boniface<br>RSS Chartered Statistician<br>Honorary Senior Research Fellow<br>Behavioural Science and Health<br>University College London

	Gower Street<br>London WC1E 6BT<br>d.boniface@ucl.ac.uk
**Trial Contacts**	Dr Ritwika Mallik<br>Clinical Research Fellow<br>Centre for Obesity Research<br>Division of Medicine<br>University College London<br>Rayne Building, 5 University Street<br>London WC1E 6JF<br>r.mallik@ucl.ac.uk<br><br>Tapiwa Ruwona<br>Research Nurse and Dietitian<br>Centre for Obesity Research<br>Division of Medicine<br>Rayne Building, 5 University Street<br>London WC1E 6JF<br>t.ruwona@ucl.ac.uk<br><br>Chloe Firman<br>Research Assistant<br>Centre for Obesity Research<br>Division of Medicine<br>Rayne Building, 5 University Street<br>London WC1E 6JF<br>chloe.firman.19@ucl.ac.uk<br><br>Nausheen Hamid<br>Dietician<br>Centre for Obesity Research<br>Division of Medicine<br>Rayne Building, 5 University Street<br>London WC1E 6JF<br>nausheen.hamid@ucl.ac.uk<br><br>Alanna Brown<br>Trial Coordinator<br>Centre for Obesity Research<br>Division of Medicine<br>Rayne Building, 5 University Street<br>London WC1E 6JF<br>alanna.brown@ucl.ac.uk
**Laboratories and central pharmacy**	Centre for Obesity Research<br>Division of Medicine<br>University College London

Docusign Envelope ID: F60CEFFC-8A22-436F-AE83-0299BDD1E22D

	Rayne Building, 5 University Street<br>London, WC1E 6JF<br>Email: j.makaronidis@ucl.ac.uk<br>Tel: 02076790991<br><br>Clinical Biochemistry<br>University College London Hospital<br>3rd Floor, 60 Whitfield St<br>London, W1T 4 EU<br>Email: debbie.mann1@nhs.net<br>Tel: 0203 447 940<br><br>Lead Pharmacist:<br>Chi Chung<br>Principal R&D Pharmacist<br>Pharmacy Department Ground Floor<br>University College London Hospitals<br>235 Euston Road<br>London, NW1 2BU<br>Chiyee.chung@nhs.net

LIST of CONTENTS

**TITLE PAGE**		**1**
**RESEARCH REFERENCE NUMBERS**		**2**
**SIGNATURE PAGE**		**3**
**PROTOCOL VERSION HISTORY**		**4**
**KEY TRIAL CONTACTS**		**5**
**i.**	**LIST OF ABBREVIATIONS**	**9**
**ii.**	**TRIAL SUMMARY**	**111**
**iii.**	**ROLE OF TRIAL SPONSOR AND FUNDER**	**144**
**iv.**	**KEY WORDS**	**144**
**1**	**BACKGROUND**	**155**
**2**	**RATIONALE**	**166**
**3**	**OBJECTIVES AND OUTCOME MEASURES/ENDPOINTS**	**2020**
**4**	**TRIAL DESIGN**	**24**
**5**	**TRIAL SETTING**	**25**
**6**	**PARTICIPANT ELIGIBILITY CRITERIA**	**255**
**7**	**TRIAL PROCEDURES**	**277**
**8**	**TRIAL TREATMENTS**	**355**
**9**	**PHARMACOVIGILANCE**	**4040**
**10**	**STATISTICS AND DATA ANALYSIS**	**499**
**11**	**DATA MANAGEMENT**	**522**
**12**	**OVERSIGHT COMMITTEES**	**544**
**13**	**MONITORING, AUDIT & INSPECTION**	**555**
**14**	**ETHICAL AND REGULATORY CONSIDERATIONS**	**555**
**15**	**DISSEMINATION POLICY**	**588**
**16**	**REFERENCES**	**599**

i. LIST OF ABBREVIATIONS

AE	Adverse Event
AR	Adverse Reaction
BDI	Beck Depression Inventory
BIA	Bioelectrical Impedance Analysis
BMI	Body Mass Index
BP	Blood Pressure
CI	Chief Investigator
CSRI	Client Service Receipt Inventory
eCRF	Electronic Case Report Form
DFU	Directions for use
DPA	Data Protection Act
DSMC	Data and Safety Monitoring Committee
DSMP	Data and Safety Monitoring Plan
DSUR	Development Safety Update Report
EMEA	European Medicines Agency
EQ-5D	EuroQol-5D
GB	Gastric Bypass
GCP	Good Clinical Practice
GLP-1	Glucagon-like peptide-1
GMP	Good Manufacturing Practice
HbA1c	Glycosylated haemoglobin
HR	Heart Rate
HRQoL	Health Related Quality of Life
IMPD	Investigational Medicinal Product Dossier
IPAQ	International Physical Activity Questionnaire
IWQOL-LITE	Impact of weight in quality of life-lite
JRO	Joint Research Office
LOCF	Last Observation Carry Forward
6MWT	6-Minute Walk Test
MHRA	Medicines and Healthcare products Regulatory Agency
NHS R&D	National Health Service Research & Development
NICE	National Institute for Health and Clinical Excellence

PI	Principal Investigator
PIS	Participant Information Sheet
PSS	Personal Social Services
QALY	Quality-Adjusted Life Year
RCT	Randomised Control Trial
REC	Research Ethics Committee
RYGB	Roux-en-Y Gastric Bypass
PYY	Peptide YY
SAR	Serious Adverse Reaction
SAE	Serious Adverse Event
STS	Sit-to-stand test
SG	Sleeve gastrectomy
SODA	Summary Of Drug Arrangements
SOP	Standard Operating Procedure
SUSAR	Suspected Unexpected Serious Adverse Reaction
T2D	Type 2 Diabetes Mellitus
TC	Trial Coordinator
TMF	Trial Master File
TMG	Trial Management Group
TSC	Trial Steering Committee
UCL	University College London
UCLH	University College London Hospital
UK	United Kingdom
UK GDPR	UK General Data Protection Regulation
WL	Weight Loss

ii. TRIAL SUMMARY

**Trial Title**	A double-blinded, randomised, placebo-controlled trial of semaglutide 2.4 mg in patients with poor weight-loss following bariatric surgery.	
**Short Title**	BARI-STEP	
**Sponsor Protocol Number**	142522	
**Clinical Phase**	Phase 3b	
**Trial Design**	Double-blinded, randomised, placebo-controlled, two -arm, parallel group trial.	
**Trial Participants**	Patients aged 18-65 years with poor weight loss following bariatric surgery (<20% of their total weight ≥ 1 year after primary gastric bypass or primary sleeve gastrectomy).	
**Planned Sample Size**	70 subjects (35 subjects in each arm)	
**Treatment duration**	Minimum of 18 months, maximum of 20 months	
**Follow up duration**	74 weeks	
**Planned Trial Period**	Approximately 26 months from when first participant enrolled to last participant follow-up.	
	**Objectives**	**Outcome Measures/Endpoints**
**Primary**	The primary objective of this trial is to compare the efficacy of 68 weeks of subcutaneous semaglutide 3.0 mg/ml or 3.2 mg/ml at a dose of 2.4mg per week versus placebo administration, as an adjunct to diet and exercise, on %WL in participants with poor weight-loss following primary GB or SG at the end of the 68 weeks of treatment.	The primary outcome of this trial is %WL from the baseline visit to 68 weeks of treatment. Percentage weight loss will be calculated using the following formula: %WL = [(weight at the baseline visit–weight at the end of the 68-week treatment period)/ weight at the baseline visit] x 100, measured at the end of treatment.
**Secondary**	To investigate the effects of semaglutide 3.0 mg/ml or 3.2 mg/ml at a dose of 2.4mg per week compared to placebo upon metabolic and inflammatory indices and HRQoL.	The secondary outcomes of this trial are:<br>1. The percentage of participants receiving subcutaneous semaglutide 3.0 mg/ml or 3.2 mg/ml at a dose of

		2.4mg per week versus placebo who after 68 weeks achieve:<br>•A body weight reduction ≥10%<br>•A body weight reduction ≥15%<br>•A body weight reduction ≥20%<br><br>2. The effect of 68 weeks of subcutaneous semaglutide 3.0 mg/ml or 3.2 mg/ml at a dose of 2.4mg per week versus placebo administration upon glycaemia, pre-diabetes and T2D by comparing:<br>•Change in circulating HbA1c levels.<br>•Change in circulating HbA1c levels in participants with pre-diabetes at baseline.<br>•Change in circulating HbA1c levels in participants with T2D at baseline.<br>•The number of pharmacological agents required for the management of T2D in participants with pre-existing T2D at baseline.<br>3. The effect of 68 weeks of subcutaneous semaglutide 3.0 mg/ml or 3.2 mg/ml at a dose of 2.4mg per week versus placebo administration upon BP and hypertension by comparing:<br>•Systolic and diastolic BP.<br>•Systolic and diastolic BP in participants with pre-existing hypertension.<br>• The number of pharmacological agents required for the management of hypertension in participants with pre-existing hypertension.

		4. The effect of 68 weeks of subcutaneous semaglutide 3.0 mg/ml or 3.2 mg/ml at a dose of 2.4mg per week versus placebo administration upon:<br>•Change in lipids and inflammatory markers (HsCRP and inflammatory cytokines).<br>•Change in food cravings.<br>•Change in HRQoL measures.<br>5. The relationship between GLP-1 levels at baseline and %WL at 68 weeks.
**Investigational Medicinal Product(s)**	Active IMP from 18 November 2022 to 04 November 2024*: Semaglutide 3.0 mg/ml solution for injection in pre-filled pen.<br><br>Active IMP from 05 November 2024 to end of trial: Semaglutide 3.2 mg/ml solution for injection in pre-filled pen.<br><br>Placebo IMP: Same composition as the IMP, but no active ingredient.<br><br>*This date marks the expiry of the current stock of semaglutide 3.0 mg/ml.<br><br>Semaglutide 3.0mg/ml is no longer being manufactured for release in the United Kingdom therefore as of 05 November 2024, the licensed semaglutide 3.2 mg/ml will be used for the trial. The maintenance dose remains at 2.4 mg of semaglutide.	
**Formulation, Dose, Route of Administration**	Semaglutide, 3.0 mg/ml or 3.2 mg/ml at a dose of 2.4 mg per week, subcutaneous injection in pre-filled pen.	

iii. ROLE OF TRIAL SPONSOR AND FUNDER

University College London (UCL) will act as sponsor for this trial. As Sponsor, UCL will provide insurance for the clinical trial and undertake to ensure that the above trial is conducted in accordance with the Medicines for Human Use (Clinical Trials) Regulations 2004 (as amended), the UK Policy Framework for Health and Social Care Research 3rd edition 2017 (as amended) and all applicable regulatory requirements.

Sponsor will provide the main REC and the MHRA with Development Safety Update Reports (DSUR) which will be written in conjunction with the trial team and the Sponsor's office. The report will be submitted within 60 days of the Developmental International Birth Date (DIBD) of the trial each year until the trial is declared ended.

iv. KEY WORDS

Bariatric surgery, obesity, poor weight loss, semaglutide, GLP-1,

Docusign Envelope ID: F60CEFFC-8A22-436F-AE83-0299BDD1E22D

1 BACKGROUND

Obesity and its associated co-morbidities represent a global health threat causing 3.4 million preventable deaths annually. Bariatric surgery is the most effective treatment for patients with severe obesity producing sustained weight-loss with reduced morbidity and mortality. As a consequence of its unparalleled health benefits, bariatric surgery has been widely adopted with ~500,000 operations undertaken annually world-wide [1]. In the UK, patients with severe obesity, defined as a body mass index (BMI) of ≥40 kg/m^2, or ≥35 kg/m^2 with an obesity-associated co-morbidity, are eligible for bariatric surgery in accordance with National Institute for Health and Clinical Excellence (NICE) Guidelines [2].

1.1 Variability in weight-loss response following bariatric surgery

Gastric bypass (GB), including Roux-en-Y Gastric Bypass (RYGB) and One Anastomosis Gastric Bypass (OAGB), and sleeve gastrectomy (SG) are the commonest bariatric procedures performed globally [1]. Whilst at a population level these operations are highly effective at reducing weight and obesity-associated morbidity and mortality, it is now clear that, at the level of the individual, weight-loss following GB and SG is highly variable [3-7]. Figure 1 shows the variability in 2-year percentage weight-loss (%WL) following RYGB and SG from our unit[3]. Given the associated surgical risks, procedure cost and the need for lifelong nutritional monitoring there is an urgent unmet clinical need to identify those with a poor weight loss response, improve weight-loss following bariatric surgery and maximise the health benefits for bariatric surgery patients. Currently, there are no available pharmacological treatments for patients with poor weight loss after surgery and the only therapeutic option is revision surgery, which carries a mortality risk and is difficult to access.

Figure 1: Weight-loss in patients who undergo sleeve gastrectomy or Roux-en-Y gastric bypass is highly variable

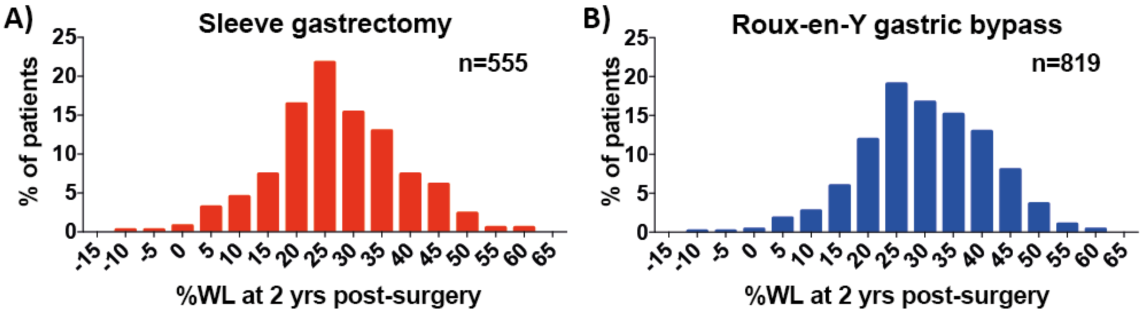

A) Percentage weight loss (%WL) in patients 2 years after sleeve gastrectomy.
B) %WL in patients 2 years after Roux-en-Y gastric bypass.

1.2 The importance of maximising post-surgery weight-loss

Following GB and SG, glycaemic control improves rapidly by weight-loss independent mechanisms [8]. However, there is increasing evidence that long-term T2D remission depends on weight-loss [9-11]. In our patients, complete T2D remission rates at 2-years post-surgery (defined as HbA1c <6%, off all medication for more than 12 months) are determined by %WL, independent of procedure type [12](Figure 2). Importantly, multivariate

logistic regression analysis correcting for confounding baseline factors shows that the odds of complete T2D remission increase by 10% for every additional 1% WL. Therefore, our data highlight the important clinical impact that small changes in %WL have on T2D remission, which has been shown to lead to a reduction in long-term microvascular disease outcomes [13].

Furthermore, the resolution and/or improvement of multiple obesity-related co-morbidities including hypertension, dyslipidaemia, obstructive sleep apnoea and non-alcoholic steatohepatitis following bariatric surgery positively correlate with increasing %WL [14-17]. In contrast, patients with poor weight loss have fewer metabolic benefits and weight regain leads to relapse of co-morbidities [14, 15]. In addition, greater improvements in HRQoL outcomes following bariatric surgery are reported with greater weight-loss [18, 19]. Taken together these findings represent a strong rationale for maximising weight-loss following bariatric surgery.

Figure 2: Rate of remission of type 2 diabetes (T2D) following sleeve gastrectomy or Roux-en-Y gastric bypass is determined by weight-loss independent of procedure type

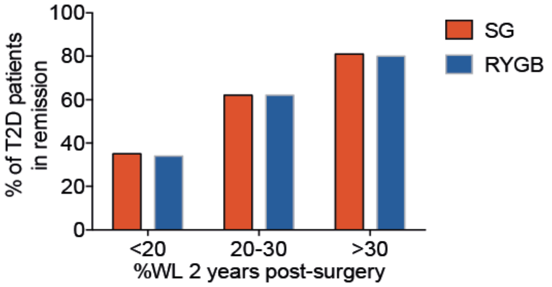

Percentage of patients with T2D at the time of surgery in complete remission at 2 years after sleeve gastrectomy (n=118) or Roux-en-Y gastric bypass (n=116) plotted according to percent weight-loss category (<20%, 20-30% and >30%).

2 RATIONALE

Liraglutide 3.0 mg (6mg/ml), a once daily subcutaneously injected glucagon-like peptide-1 (GLP-1) receptor analogue, has been shown to lead to significant weight loss in people with obesity and is licensed for weight management as an adjunct to lifestyle modification in people with obesity. The SCALE study (ClinicalTrials.gov number, NCT01272219), a 56-week double-blind trial, investigating liraglutide 3.0 mg versus placebo in people with obesity or overweight without T2D (BMI>30 kg/m^2 or >27 kg/m^2 with dyslipidaemia or hypertension) in addition to lifestyle modification, showed a mean %WL of 8.0 ± 6.7% in patients treated with liraglutide 3.0 mg compared to 2.6 ± 5.7% in the placebo group[20]. 63% of patients treated with liraglutide compared to 27.1% in the placebo group lost ≥5% body weight. 33% of liraglutide-treated patients lost ≥10% compared to 10.6% in the placebo group. The trial also showed greater reductions in HbA1c, fasting glucose and fasting insulin levels in the liraglutide 3.0 mg group compared to the placebo group. Patients in the liraglutide 3.0 mg group also had higher SF-36 scores, indicating improved physical and mental health and higher IWQoL-Lite scores (indicating better quality of life) compared to the placebo group. Overall, the SCALE trial demonstrated that liraglutide 3.0 mg, used as an adjunct to diet and exercise, leads to reduced body weight and improved metabolic health.

Liraglutide 3.0 mg was first used in the post-bariatric surgery population by Wharton *et al*. in a population of 117 post-bariatric surgery patients with poor weight loss, in order to determine if this would differ in three types of

bariatric procedure: RYGB, SG and gastric banding [21]. The majority of patients in this study had undergone RYGB (45.3%) or gastric banding (42.7%). A significant weight loss was noted in all participants from 2 months following initiation of liraglutide 3.0 mg compared to baseline, which was maintained following 1 year of treatment ($p<0.05$). %WL was 6.6 ± 7.1% following RYGB with 24.5% of patients achieving ≥ 10% WL; 4.9 ± 5.6% following gastric banding with 12.0% achieving ≥ 10% WL and 3.6 ± 3.0% post-SG. No significant differences were seen in weight loss between the three procedures. Nausea was the most commonly reported side effect, reported by 29.1% patients. Three participants discontinued due to an adverse event, which in two cases was an allergic reaction and one participant developed pancreatitis.

Suliman *et al.* performed a prospective study using liraglutide 3.0 mg in people with obesity and people with poor weight-loss outcomes following bariatric surgery[22]. 787 patients were treated for ≥16 weeks and their median % WL was 6.4% with 23% achieving ≥ 10% WL. The study showed no difference in %WL in patients who had not had bariatric surgery and those who had poor weight loss outcomes following bariatric surgery. The majority of adverse events reported were mild gastrointestinal side effects and treatment was discontinued in one participant due to pancreatitis.

We performed a 24-week randomised-controlled double-bind trial of liraglutide 3.0 mg versus placebo in patients with poor weight loss following RYGB or SG and a sub-optimal meal-stimulated GLP-1 response (EudraCT 2017-002407-10). 70 patients ≥1 year following either RYGB or SG with <20% weight loss and a suboptimal meal-stimulated GLP-1 response were randomised to either receive liraglutide 3.0 mg or placebo for 24 weeks in addition to diet and lifestyle counselling.
This study showed that patients treated with liraglutide 3.0mg had mean weight loss of 9.41 ±5.84% at the end of the 24-week treatment period compared to 1.10 ± 5.26% in the placebo group with an estimated treatment effect of -8.80 (95% CI -11.86 to -5.73) ($p<0.005$). 77.4% of participants in the liraglutide group lost >5% compared to 9.3% in the placebo group ($p<0.001$). 32.2% of patients in the liraglutide arm had ≥10% WL and 16.1% ≥15% WL. No participants in the placebo group lost ≥10% of their body weight (10% WL: 32.2% vs. 0% $p<0.05$; ≥15% WL: 16.1% vs. 0% $p<0.05$). Our findings highlight larger %WL compared to both the SCALE trial, despite a shorter duration, and the open label studies by Wharton *et al.* and Suliman *et al.*

Liraglutide 3.0 mg was well tolerated in our study. There were no serious adverse events in either group. The most commonly reported adverse events were nausea and constipation. Vomiting was only reported by 3% of patients in the liraglutide 3.0 mg arm and by 3% of participants in the placebo arm. In contrast in the SCALE study, vomiting was reported in 16.3% in the liraglutide 3.0 mg arm versus 4.1% in the placebo arm. Our findings illustrate that liraglutide 3.0 mg represents an effective, safe treatment to improve %WL for patients with poor weight loss following RYGB and SG.

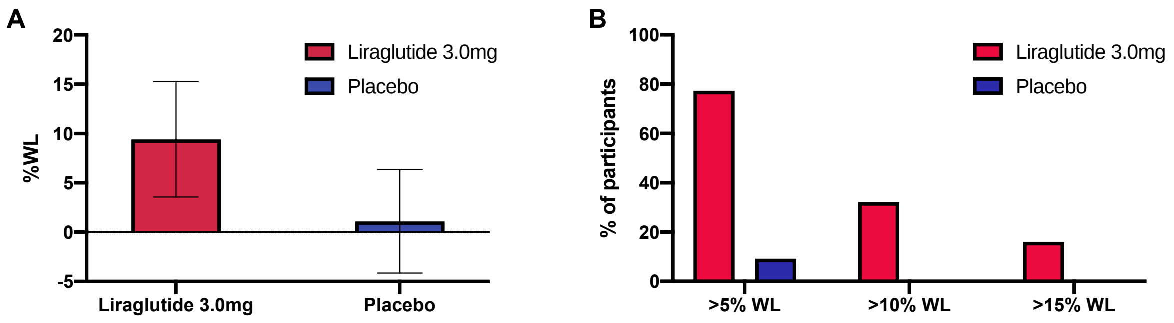

Figure 3: Mean weight loss at 24-weeks in liraglutide 3.0 mg vs placebo in patients with poor weight loss ≥1 year following either RYGB or SG (A) and percentage of participants achieving ≥ 5%, ≥ 10% and ≥ 15% in liraglutide 3.0 mg vs placebo (B)

Semaglutide is a long-acting GLP-1R agonist, which is injected subcutaneously once weekly for the treatment of type 2 diabetes mellitus. At present, semaglutide for weight management has been granted marketing authorisation in the UK as of 24th September 2021 (PL number PLGB 04668/0433). The Semaglutide Treatment Effect in People with obesity (STEP) is a phase 3 clinical trial programme comprised of five different trials, with the aim of evaluating the efficacy and safety of semaglutide 3.0 mg/ml administered subcutaneously once weekly, for weight management in people with obesity or overweight.

In STEP 1, 1,961 adults with obesity or overweight, without T2D, were being randomly assigned in a 2:1 manner to receive semaglutide 3.0 mg/ml or placebo as an adjunct to lifestyle intervention for 68 weeks. Mean body weight change from baseline to week 68 was −14.9% in the semaglutide 3.0 mg/ml group vs. −2.4% with placebo (estimated treatment difference: −12.4% [95% confidence interval: −13.4%; −11.5%]; p<0.0001. Nausea and diarrhoea (typically transient and mild-to-moderate) were the most common adverse events with semaglutide 3.0 mg/ml; these subsided with time. Treatment discontinuation due to gastrointestinal events more frequently with semaglutide 3.0 mg/ml (4.5%) than placebo (0.8%). Treatment discontinuation in STEP 1 was lower compared to the SCALE trial where 9.9% in the liraglutide 3.0 mg group withdrew due to adverse events and 2.9% in the placebo group[20].

The Coronavirus Infectious Disease 2019 (COVID-19) pandemic has highlighted the need to maximise health outcomes for people living with obesity. People living with obesity are at an increased risk of developing severe disease with COVID-19 and obesity is associated with an increased mortality from COVID-19[23]. Obesity leads to a pro-inflammatory state with increased production of inflammatory cytokines, which can result in impaired immune responses in people with obesity. Together with increased prevalence of co-morbidities and the mechanical stress of adiposity on lung function, this impaired immune function and inflammatory state have been proposed as a link between obesity and poor outcomes with COVID-19[24, 25]. In addition to improvement in co-morbidities, bariatric surgery has been shown to improve immune function[26]. Importantly, a study investigating outcomes in people with obesity who had undergone bariatric surgery and lost weight following their operation, showed lower rates of hospital and intensive care admission in patients with obesity who developed COVID-19[24].

Data from the STEP program illustrate that semaglutide 3.0 mg/ml is more efficacious compared to liraglutide 3.0 mg/ml: 75% of people achieved ≥10% weight loss with semaglutide 3.0 mg/ml versus 33% with liraglutide 3.0 mg/ml. More importantly 55% achieved ≥ 15% weight loss with semaglutide 3.0 mg/ml versus 14% with liraglutide 3.0 mg/ml. Considering the promising results from our trial showing liraglutide 3.0 mg leads to approximately 10% WL in patients with poor weight loss following bariatric surgery and the results from the STEP program, we hypothesise that semaglutide 3.0 mg/ml or 3.2 mg/ml will lead to greater weight loss and health improvement compared to placebo in patients with poor weight loss following GB or SG. We plan a 68-week double-blind randomised placebo-controlled trial to assess the efficacy of semaglutide 3.0 mg/ml or 3.2 mg/ml in people with <20% WL ≥ 1 year following GB or SG, in terms of %WL, number of patients achieving ≥ 10% WL, ≥15% WL, >20% WL, metabolic and inflammatory markers and health economics/quality of life parameters.

2.1 Assessment and management of risk

The table below summarises the risks, frequencies and mitigations of the Investigational Medicinal Product (IMP). and Non-Investigational Medicinal Products (NIMP).

**Name of IMP**<br>Semaglutide 3.0 mg/ml or 3.2 mg/ml	**Potential risk**<br>Injection site reactions	**Risk Frequency**<br>Common	**Risk Management**<br>1. Participants will be trained on injection technique and instructed to follow the dose escalation schedule.
	Nausea, Vomiting, Constipation, Diarrhoea, Abdominal Pain, Fatigue, Headache<br>Dizziness, Gastritis, Gastrooesophageal reflux disease, Dyspepsia, Eructation, Flatulence, Abdominal distension, Cholelithiasis, Hair loss	Very Common<br>Common	1. Close monitoring by trial investigators. Antiemetics will provided if required to manage gastrointestinal side effects.<br>2. Participants will be given a 24-hour Contact Card and a drug diary.

The table below summarise the risks and mitigations of all tests and/or procedures above standard care that are being performed:

**Intervention**	**Potential risk**	**Severity Grading**	**Risk Management**
Blood Test/Cannulation	Bruising	Mild	1.Performed by trained healthcare professionals.

	Pain<br>Bleeding<br>Infection	Mild<br>Mild/Moderate<br>Moderate/Severe	2.Follow trust standard operational procedures.<br>3.Provision of 24h contact card

This trial is categorised as: Type B = Somewhat higher than the risk of standard medical care

3 OBJECTIVES AND OUTCOME MEASURES/ENDPOINTS

In patients with poor weight-loss following GB or SG, to determine whether 68 weeks of subcutaneous semaglutide 3.0 mg/ml or 3.2 mg/ml with diet and exercise counselling causes greater %WL and reduction in adiposity than placebo with diet and exercise counselling, and to estimate the extent of any reduction. Investigation will also be carried out with regard to improvement in metabolic and inflammatory indices and HRQoL.

3.1 Primary objective

The primary objective of this trial is to compare the efficacy of 68 weeks of subcutaneous semaglutide 3.0 mg/ml or 3.2 mg/ml at a dose of 2.4 mg per week versus placebo administration, as an adjunct to diet and exercise, on %WL in participants with poor weight-loss following primary GB or SG at the end of the 68 weeks of treatment.

3.2 Secondary objectives

1. To compare the percentage of participants receiving subcutaneous semaglutide 3.0 mg/ml or 3.2 mg/ml at a dose of 2.4 mg per week versus placebo who after 68 weeks achieve:

- A body weight reduction ≥10%
- A body weight reduction ≥15%
- A body weight reduction ≥20%

2. The effect of 68 weeks of subcutaneous semaglutide 3.0 mg/ml or 3.2 mg/ml at a dose of 2.4 mg per week versus placebo administration upon glycaemia, pre-diabetes and T2D by comparing:

- Change in circulating HbA1c levels.
- Change in circulating HbA1c levels in participants with pre-diabetes at baseline.
- Change in circulating HbA1c levels in participants with T2D at baseline.
- The number of pharmacological agents required for the management of T2D in participants with pre-existing T2D at baseline.

3. The effect of 68 weeks of subcutaneous semaglutide 3.0 mg/ml or 3.2 mg/ml at a dose of 2.4 mg per week versus placebo administration upon BP and hypertension by comparing:

- Systolic and diastolic BP.

- Systolic and diastolic BP in participants with pre-existing hypertension.
- The number of pharmacological agents required for the management of hypertension in participants with pre-existing hypertension.

4. To compare the effect of 68 weeks of subcutaneous semaglutide 3.0 mg/ml or 3.2 mg/ml at a dose of 2.4 mg per week versus placebo administration upon:

- Change in circulating lipids and inflammatory markers (HsCRP and inflammatory cytokines).
- Changes in food cravings
- Change in HRQoL measures.

5. To investigate the relationship between fasted and meal-stimulated active GLP-1 levels at baseline and %WL at 68 weeks.

3.3 Outcome measures/endpoints

3.3.1 Primary endpoint/outcome

The primary outcome of this trial is %WL from the baseline visit to 68 weeks of treatment. Percentage weight loss will be calculated using the following formula: %WL = [(weight at the baseline visit–weight at the end of the 68-week treatment period)/ weight at the baseline visit] x 100, measured at the end of treatment.

3.3.2 Secondary endpoints/outcomes

The secondary outcomes of this trial are:

1. The percentage of participants receiving subcutaneous semaglutide 3.0 mg/ml or 3.2 mg/ml at a dose of 2.4 mg per week versus placebo who after 68 weeks achieve:

   - A body weight reduction ≥10%
   - A body weight reduction ≥15%
   - A body weight reduction ≥20%

2. The effect of 68 weeks of subcutaneous semaglutide 3.0 mg/ml or 3.2 mg/ml at a dose of 2.4 mg per week versus placebo administration upon glycaemia, pre-diabetes and T2D by comparing:

- Change in circulating HbA1c levels.
- Change in circulating HbA1c levels in participants with pre-diabetes at baseline.
- Change in circulating HbA1c levels in participants with T2D at baseline.
- The number of pharmacological agents required for the management of T2D in participants with pre-existing T2D at baseline.

3. The effect of 68 weeks of subcutaneous semaglutide 3.0 mg/ml or 3.2 mg/ml at a dose of 2.4 mg per week versus placebo administration upon BP and hypertension by comparing:

- Systolic and diastolic BP.
- Systolic and diastolic BP in participants with pre-existing hypertension.
- The number of pharmacological agents required for the management of hypertension in participants with pre-existing hypertension.

4. The effect of 68 weeks of subcutaneous semaglutide 3.0 mg/ml or 3.2 mg/ml at a dose of 2.4 mg per week versus placebo administration upon:
    - Change in lipids and inflammatory markers (HsCRP and inflammatory cytokines).
    - Change in food cravings.
    - Change in HRQoL measures.

5. The relationship between GLP-1 levels at baseline and %WL at 68 weeks.

3.3.3 Table of endpoints/outcomes

**Objectives**	**Outcome Measures**	**Timepoint(s) of evaluation of this outcome measure (if applicable)**
**Primary Objective**<br>The primary objective of this trial is to compare the efficacy of 68 weeks of subcutaneous semaglutide 3.0 mg/ml or 3.2 mg/ml at a dose of 2.4 mg per week versus placebo administration, as an adjunct to diet and exercise, on %WL in participants with poor weight-loss following primary GB or SG at the end of the 68 weeks of treatment.	Percentage weight loss will be calculated using the following formula: %WL = [(weight at the baseline visit–weight at the end of the 68-week treatment period)/ weight at the baseline visit] x 100, measured at the end of treatment.<br>The mean difference in %WL at 68 weeks between the groups will be analysed using linear regression, adjusting for stratification variables and any baseline variables which are not balanced between the groups. Mean difference in %WL will be reported with 95% confidence interval. The assumptions of the model will be checked, and a suitable transformation/non-parametric method will be used where the assumptions are not met. All available data will be analysed as randomised. Bias due to missing data will be investigated and dealt as appropriate.	At baseline and at each follow-up visit, corresponding to weeks: 1, 2, 4 ,6, 8, 10, 12, 14, 16, 20, 24, 28, 32, 36, 40, 44, 48, 52, 56, 60, 64 and 68.

**Secondary Objectives**<br>1. To compare the percentage of participants receiving subcutaneous semaglutide 3.0 mg/ml or 3.2 mg/ml at a dose of 2.4 mg per week versus placebo who after 68 weeks achieve:<br>•A body weight reduction ≥10%<br>•A body weight reduction ≥15%<br>•A body weight reduction ≥20%<br>2. The effect of 68 weeks of subcutaneous semaglutide 3.0 mg/ml or 3.2 mg/ml at a dose of 2.4 mg per week versus placebo administration upon glycaemia, pre-diabetes and T2D by comparing:<br>•Change in circulating HbA1c levels.<br>•Change in circulating HbA1c levels in participants with pre-diabetes at baseline.<br>•Change in circulating HbA1c levels in participants with T2D at baseline.<br>•The number of pharmacological agents required for the management of T2D in participants with pre-existing T2D at baseline.<br><br>3. The effect of 68 weeks of subcutaneous semaglutide 3.0 mg/ml or 3.2 mg/ml at a dose of 2.4 mg per week versus placebo administration upon BP and hypertension by comparing:<br>•Systolic and diastolic BP.<br>•Systolic and diastolic BP in participants with pre-existing hypertension.<br>•The number of pharmacological agents required for the management of hypertension in	The results of the secondary analysis will be treated as exploratory. Continuous outcomes will be analysed using separate linear regression models, adjusting for stratification variables and any baseline variables which are not balanced between the groups. Mean differences in each outcome will be reported with 95% confidence intervals. The assumptions of each model will be checked, and a suitable transformation/non-parametric method will be used where the assumptions are not met.	Evaluation of secondary outcome measure taken at 68 weeks after randomisation.

participants with pre-existing hypertension.<br><br>4. To compare the effect of 68 weeks of subcutaneous semaglutide 3.0 mg/ml or 3.2 mg/ml at a dose of 2.4 mg per week versus placebo administration upon:<br><br>•Change in circulating lipids and inflammatory markers (HsCRP and inflammatory cytokines).<br><br>•Changes in food cravings<br><br>•Change in HRQoL measures.<br><br>5. To investigate the relationship between fasted and meal-stimulated active GLP-1 levels at baseline and %WL at 68 weeks.		

4 TRIAL DESIGN

This study is a 68-week double-blind, randomised, placebo-controlled, two-arm, parallel group trial (Figure 4). The purpose of this trial is to evaluate the therapeutic effects of semaglutide 3.0 mg/ml or 3.2 mg/ml in patients with ‘poor’ weight loss (<20% WL) following bariatric surgery. Enrolled participants will be randomised 1:1 to receive semaglutide at a dose of 2.4 mg per week or placebo, stratified for sex, procedure type and T2D. The primary outcome will be %WL following 68 weeks of treatment. 35 participants will be randomised to receive semaglutide 2.4mg (n = 35) or placebo (n = 35) for 68 weeks. An identical placebo containing no active ingredients will be used as a comparator to evaluate the real treatment effect. Treatment allocation will be concealed from patients and investigators.

Semaglutide 3.0 mg/ml or 3.2 mg/ml at dose of 2.4 mg will be used and will follow a 16-week dose escalation protocol. Follow-up visits will take place at 1, 2 ,4 ,6, 8, 10, 12, 14, 16, 20, 24, 28, 32, 36, 40, 44, 48, 52, 56, 60, 64 and 68 weeks after randomisation. The majority of these visits will be carried out remotely. Data including weight, adverse events and concomitant medication use will be collected at each visit. The visits at weeks 6, 14, 32, 52 and 68 will be carried out in person, to allow a targeted physical examination to be performed and blood samples to be taken for biochemical monitoring. Participants will remain in the trial for an additional 6-week wash out period with remote monitoring visits at week 70 and 74.

Docusign Envelope ID: F60CEFFC-8A22-436F-AE83-0299BDD1E22D

4.1 Trial Flow Chart

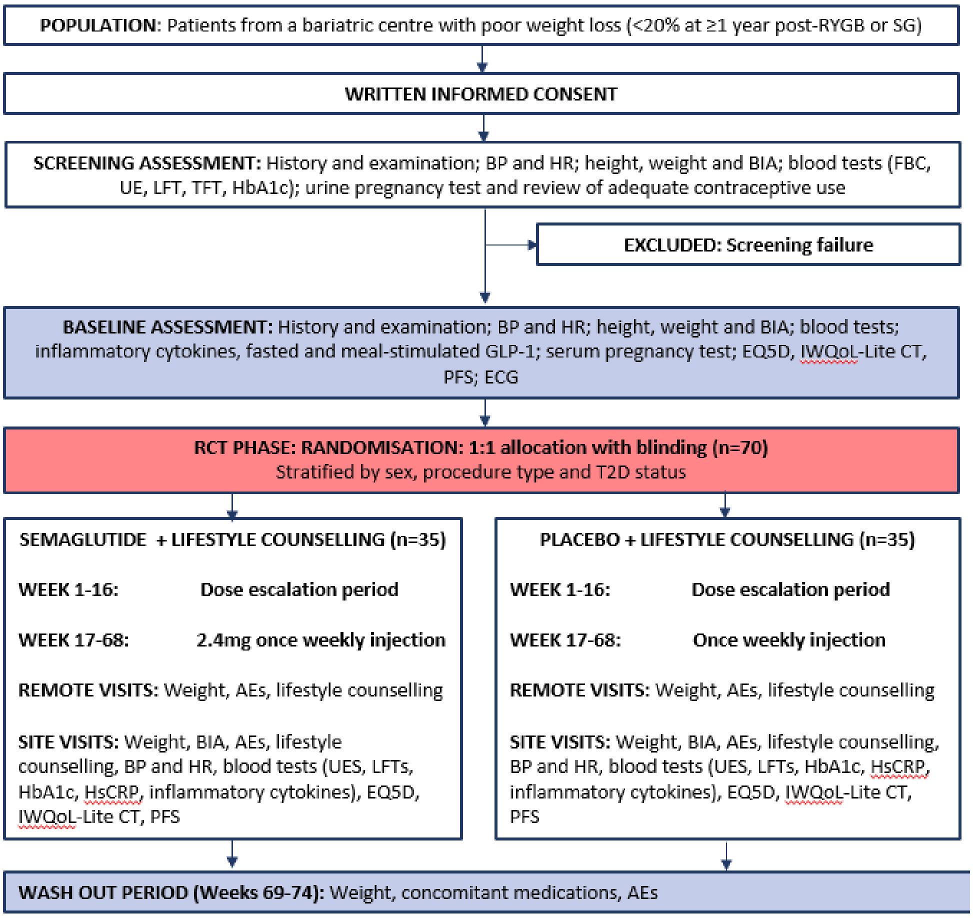

5 TRIAL SETTING

This is a single centre trial and will be conducted at University College London Hospitals (UCLH). At first, suitable patients will be identified by the bariatric team during their post-surgery follow-up visits at face-to-face or virtual clinic appointments, and through database search and asked if they are interested to take part in the study. Participants will be recruited from the Bariatric Clinics at University College London Hospital (UCLH).

6 PARTICIPANT ELIGIBILITY CRITERIA

Study population: patients, 1 year or more following primary GB or primary SG, with poor weight-loss response (<20% WL) will be invited to participate. There will be no waivers to the inclusion and exclusion criteria.

The Sponsor does NOT allow the use of "protocol waivers" or departures from the approved inclusion/exclusion criteria of the protocol. Occurrences of this nature may constitute a serious breach and be reportable to the MHRA.

6.1 Inclusion Criteria

1. Patients, ≥1 year primary GB or primary SG, with poor weight-loss (<20% WL) that is not caused by either a surgical or psychological problem.
2. Adults, 18-65 years inclusive.
3. Females of childbearing potential and female partners of male participants must be willing to use highly effective method of contraception (hormonal or barrier method of birth control; abstinence) (Appendix 2) from the time consent is signed until 2 months after treatment discontinuation.
4. Male participants with partners of childbearing potential must use barrier methods of contraception for the duration of the trial until 2 months after treatment discontinuation.
5. Females of childbearing potential must have a negative serum pregnancy test within 7 days prior to randomisation. NOTE: Subjects are considered not of childbearing potential if they are surgically sterile (i.e. they have undergone a hysterectomy, bilateral salpingectomy or bilateral oophorectomy) or they are postmenopausal.
6. A self-reported ≤5 % variation in body weight over preceding 3 months.
7. Fluent in English and able to understand and complete questionnaires.
8. Participants capable to provide written informed consent and comply with the trial protocol.

6.2 Exclusion criteria

1. Bariatric surgical procedure other than GB and SG, or revision bariatric surgery of any operation type.
2. Personal history of type I diabetes or type II diabetes mellitus currently treated with insulin.
3. Concomitant use of GLP-1R agonist or DPPIV-inhibitors.
4. Female who is pregnant, breast-feeding, or intends to become pregnant.
5. Current participation in other clinical intervention trial.
6. History of suicidal attempt in the previous 5 years or untreated severe depression or mental health condition assessed by direct questioning.
7. Symptomatic gallstone disease
8. Uncontrolled hypertension (systolic blood pressure ≥ 160 mmHg or diastolic blood pressure ≥ 100 mmHg).
9. Renal impairment measured as glomerular infiltration rate (eGFR <15 ml/min 1.73 m^2)
10. Known or suspected hypersensitivity to semaglutide or any of the excipients involved in their formulation.
11. Personal or family history of medullary thyroid carcinoma or multiple endocrine neoplasia syndrome type 2.
12. History of malignant neoplasms within the past 5 years prior to screening. Basal and squamous cell skin cancer and any carcinoma in-situ are allowed.
13. Personal history of acute pancreatitis 180 days before screening or chronic pancreatitis.
14. Uncontrolled thyroid disease.
15. History of stroke, unstable angina, acute coronary syndrome, congestive heart failure New York Heart Association class III-IV within the preceding 12 months.
16. Untreated clinically significant arrhythmias.
17. Diabetic gastroparesis.
18. Concomitant usage of medications that cause weight gain or weight loss.
19. Known or suspected abuse of alcohol or recreational drugs.
20. Severe hepatic impairment diagnosed via liver function blood tests and clinical evaluation

21. Any additional factor, which in the investigator's opinion, might jeopardise the subject's safety or compliance with the trial protocol.

7 TRIAL PROCEDURES

A schedule of all trial assessments and procedures is set-out in Appendix 1.

7.1 Recruitment

Participant recruitment will only commence following written confirmation by the Sponsor, and the trial site has been issued a Trust Confirmation of Capacity and Capability and Open to Recruitment Letter. The clinical bariatric teams at UCLH will identify patients potentially fulfilling the eligibility criteria either when these attend follow-up clinics, or through search of existing databases. Patients will be considered to be enrolled into the trial following: consent, baseline assessments, randomisation, allocation of the participant trial number and intervention.

7.1.1 Participant Identification

Participant recruitment at a site will only commence when the trial has:

1. Been initiated by the Sponsor (or it's delegated representative), and
2. Issued with the 'Open to Recruitment' letter.

7.2 Informed Consent

The person taking consent will be GCP trained, suitably qualified and experienced, and have been delegated this duty by the CI on the Staff Signature and Delegation of Tasks Log. At first, suitable patients will be identified by the bariatric team during their post-surgery follow-up visits at face-to-face or virtual clinic appointments and asked if they are interested to take part in the study. In addition, potentially suitable patients might be identified through database searches by members of the clinical team. In this instance, patients who have expressed their interest in participating in research projects will be approached by the clinical team (via email, phone call) to see whether they might be interested in the trial. Information about interested subjects will be collected by the clinical team onto the study screening log and the contact will be logged in their medical records. Verbal consent or email consent (for those contacted by email who email back) will be sought from patients before they are approached by a research investigator who will explain the screening procedure as well as the aims, methods, anticipated benefits and potential risks of the study and provide a copy of the Participant Information Sheet (PIS). The investigator will also explain that patients are under no obligation to undergo screening and enter the trial and that they can withdraw at any time without having to give a reason. Those patients interested in taking part in the trial will be invited to attend a face-to-face visit in order to undergo screening for this trial. Written informed consent will only be sought after a minimum of 24 hours after being sent the PIS. When attending the screening visit, potential participants will be asked to sign two copies of the study consent form, which will be also countersigned by the research investigator conducting the assessment: one copy will be for the participant to retain and the other will be filed in the study site file (a copy will be also uploaded in the patient's medical records). However, consent will not denote enrolment into the study. Participants will be informed that their suitability to participate in the study will only be confirmed following review of screening data and that they may be excluded based on the study's inclusion and exclusion criteria. The consent process will be documented in the medical notes for a clear audit trail.

If a participant does not meet all the inclusion and exclusion criteria no further action will be taken and the participant will be informed that, for their own safety, it is not appropriate they continue with the study. Screening failure patients might be contacted for other research studies should they be interested (the option of being considered for future research is included in the consent form and they have consented to this). In addition, depending on the reason for screening failure, such patients might be contacted again in the future and asked if willing to be re-screened, should they still wish to take part to the trial. A research investigator will notify participants of screening outcome via phone call or email. The investigator will explain again study aims, methods, anticipated benefits and potential risks to qualifying patients and answer any questions they may have. The investigator will also explain that participants are under no obligation to enter the trial and that they can withdraw at any time during the trial, without having to give a reason. No clinical trial procedures will be conducted prior to the participant giving consent by signing the Consent Form.

The investigator obtaining consent will register the participant by entering all screening data regarding the participant on the database. The system will then assign a unique participant identification number (PIN) to that participant that needs to be recorded on the consent form. No eligibility waivers or deviations will be permitted. If new safety information results in significant changes in the risk/benefit assessment, the PIS and consent form will be reviewed and updated if necessary and subjects will be re-consented as appropriate. Therefore, the version and date of the PIS and ICF in use at the time will be recorded in the medical notes.

7.3 Screening

Interested eligible patients will be invited to attend a screening assessment visit. On the day of the screening visit, the investigator will explain the trial, the procedures involved and will answer any questions the participants may have. Before any procedure is carried out, participants will be asked to sign 2 copies of the trial consent form, which will be countersigned by the research investigator conducting the visit. One copy of the consent form will be given to the participant to keep for their records and the second copy will be filed in the trial site file. All participants who have consented for the trial must meet all the inclusion and exclusion criteria as set out in section 6.1 and 6.2. No eligibility waivers or deviations will be permitted.

The following screening procedures will be carried out:

- Physical examination.
- Vital signs (heart rate (HR) and blood pressure (BP).
- Medical history and co-morbidities.
- Concomitant medications.
- Urine pregnancy test for women of childbearing potential.
- Weight and height.
- Blood tests:
    - Haematology: full blood count.
    - Serum biochemistry: renal, liver and thyroid function, HbA1c.

Following the visit, once all data related to the screening visit has been obtained, the investigator (a qualified healthcare professional) will review the data to ensure that the participant is eligible to take part in the trial. An investigator will call/email the patient to notify of the outcome of screening, answer their questions and explain possible next steps (depending on outcome). Subjects who are deemed not to be eligible will be advised that they will be unable to be re-screened at a later date.

All pre-treatment procedures will be carried out as specified in the schedule of assessments (Appendix 1).

7.4 The randomisation Procedures

Participant registration will be undertaken centrally by the coordinating trial team.
Following participant consent, and confirmation of eligibility (see section 7.3 - Screening) the registration procedure described below will be carried out.

Participants are considered to be enrolled into the trial following: consent, pre-treatment screening assessments (see section 7.3 - Screening), confirmation of eligibility, completion of the registration, and allocation of the participant trial number by the central coordinating team.

7.4.1 Method of implementing the randomisation/allocation sequence

Following participant consent, screening and baseline measure collection, the randomisation procedure will be remotely carried out through an online randomisation service (Sealed Envelope Ltd.), with provision to enable unblinding if required. Subjects will be randomly (stratified block randomisation with varying block sizes) assigned in a 1:1 ratio to receive either semaglutide 2.4 mg or placebo, stratified for sex, type of surgical procedure and T2D status. The investigator will provide participant's initials, date of birth and stratification information before a randomisation code can be generated for each participant. Both participants and investigators will be blinded to study-group assignments. Once randomisation has been performed, no group allocation will be disclosed. A randomisation notification email will be sent to the pharmacist reporting the participant randomisation code; this randomisation code will reveal group allocation when identified in the code list. Only the trial coordinator and the trial pharmacist will have access to the code list. The blinding of the trial will be maintained throughout the trial until all data entry and processing are complete, the database has been locked and data analysis performed. Participants will be given a 24-hour Contact Card for emergency unblinding if required, medical support, or for any enquiries they have throughout the study period. For details on unblinding procedure refer to the trial Randomisation, Unblinding and Code break SOP.

7.5 Blinding

Both participants and all study staff carrying out trial visits/collecting study data will be blinded to study-group assignments. The study drugs, i.e., IMP and NIMP, will be identical in appearance. The only difference will be the lack of the active ingredient (semaglutide) in the placebo formulation. The blinding of the trial will be maintained throughout the trial until all data entry and processing are complete, the database has been locked and primary analysis performed.

7.5.1 Unblinding

During the trial, trial code will only be broken for valid medical or safety reasons e.g., in the case of a serious adverse event where it is necessary for the treating health care professional to know which treatment the participant is receiving before providing appropriate treatment. Subject always to clinical need, where possible, members of the research team will remain blinded. For details on unblinding procedure refer to the trial Randomisation, Unblinding and Code break SOP.

In the event a code is required to be unblinded a formal request to the holder of the code break list, or their delegate, will be made and the unblinded information obtained. The pharmacist and the Trial Co-ordinator will be the solely holders of the code list which will be provided by Sealed Envelope and will unblind via sealedenveleops.com. In the event a code is required to be unblinded, a formal request to the holder of the code break list will be made and the unblinded information obtained.

The CI/PI will document the breaking of the code and the reasons for doing so on the CRF/data collection tool, in the site file and medical notes. It will also be documented at the end of the study in any final study report and/or statistical report. The CI/Investigating team will notify the JRO (acting on behalf of the Sponsor) in writing as soon as possible following the code break detailing the necessity of the code break.

The Sponsor will also notify the relevant authorities as required. The written information will be disseminated to the Data and Safety Monitoring Committee (DSMC) for review in accordance with the DSMC Charter.

7.5.2 Emergency Unblinding

The trial code should only be broken for valid medical or safety reasons e.g., in the case of a serious adverse event where it is necessary for the investigator or treating health care professional to know which treatment the participant is receiving before the participant can be treated. Subject to clinical need, where possible, members of the research team should remain blinded. If a treating physician, who is not the CI/PI/trial investigator, requires the treatment to be unblinded in an emergency situation, they should notify the Investigating team that an emergency unblinding is required for a trial subject. The investigator/research team will provide this information as quickly as possible. On receipt of the treatment allocation details the CI/PI/trial investigator, or treating health care professional, will deal with the participant's medical emergency as appropriate.

In the event a code is required to be unblinded a formal email request, or phone call, to the holder of the code break list will be made and the unblinded information obtained. The pharmacist and the Trial Co-ordinator will be the solely holders of the code list. Outside normal working hours, UCLH Pharmacy will not provide unblinding support, therefore, any request will be dealt by the Trial coordinator. The pharmacist and trial manager will unblind via sealedenvelope.com.

7.6 Baseline Data

Prior to the scheduled assessment, a member of the study team will contact the participants to remind them to fast for 12 hours prior to the study visit and drink only water, as detailed in the PIS. They will be advised to avoid heavy exercise and alcohol consumption the evening prior to the visit. This contact will be noted in the medical records. The following baseline assessments will be carried out:

- Sociodemographic data.
- Physical examination.
- Vital signs (HR and BP).
- Serum pregnancy test for women of childbearing potential.
- Weight.
- BIA.
- ECG
- Blood tests:
    - Haematology: full blood count.
    - Serum biochemistry: renal, liver and thyroid function, HbA1c (If baseline visit >7 days from screening), glucose, lipids
    - HsCRP and inflammatory cytokines
    - Fasted and meal-stimulated GLP-1 levels: Prior to and 30 minutes after a standardised liquid meal. (Patients will be given a choice of cannulation or venepuncture when drawing baseline bloods for GLP-1 level analysis)
- Questionnaires:
    - ✓ Impact of weight on quality of life-lite (IWQOL-Lite CT)
    - ✓ SF-36 physical functioning score
    - ✓ Power of food questionnaire
    - ✓ EuroQol-5D (EQ-5D) [28, 29]
- Counselling on lifestyle modification (500-kcal deficient diet and 150 minute of physical activity/week and resistance exercise).
- Distribution of:
    - ✓ Scale
    - ✓ Drug diary
    - ✓ Semaglutide 3.0 mg/ml or 3.2 mg/ml /placebo pre-filled pens, injection and sharps bins.
- Subcutaneous injection training. Participants will be contacted by the study team to assess their injection technique. They will be offered additional injection training by the research investigator as required.

7.7 Trial Assessments

68-week trial of once weekly subcutaneously injected escalating semaglutide 3.0 mg/ml or 3.2 mg/ml or placebo as an adjunct to diet and exercise. Participants will receive on-going counselling for diet and exercise throughout the trial period by healthcare professionals. Semaglutide 3.0 mg/ml or 3.2 mg/ml and placebo will be supplied as a solution for injection in a pre-filled dial-a-dose pen-injector containing placebo or semaglutide 3.0 mg/ml or 3.2 mg/ml. Study medication will only be administered once all visit 2 assessments have been completed and the relevant safety reports have been obtained. All eligibility criteria have to be fulfilled and no exclusion criteria must be identified.

7.8 Long term follow-up assessments

Follow-up trial visits to monitor participants will be carried out at week 1, 2, 4, 6, 8, 10, 12, 14, 16, 20, 24, 28, 32, 36, 40, 44, 48, 52, 56, 60, 64 and 68 weeks. The majority of these visits will be carried out remotely. Data including weight, adverse events and concomitant medication use will be collected at each visit. The visits at weeks 6, 14, 32, 52 and 68 weeks will be carried out in person for safety purpose, to allow a targeted physical examination to be performed and blood samples to be taken for biochemical monitoring.

Visits 3-5, 7-9, 11-14, 16-19, 21-23: Intervention phase remote visits (Weeks 1, 2, 4, 8, 10, 12, 16, 20, 24, 28, 36, 40, 44, 48, 56, 60, 64) (+/- 3 days, +/- 7 days from visit 16 onwards)

The following will be assessed at each of the trial visits:

- Weight (using the scales provided to the participants).
- Concomitant medications.
- Compliance with trial treatment.
- Nutritional and exercise counselling.
- Adverse events (AE) review.

Visits 6, 10, 15, 20: Intervention phase site visits (Weeks 6, 14, 32, 52) (+/- 3 days; +/- 7 days for visit 20)

The following will be assessed at each of the trial visits:

- Targeted physical examination.
- Vital signs (HR and BP).
- Weight.
- BIA.
- Blood tests:
    - Haematology: full blood count.
    - Serum biochemistry: renal, liver, HbA1c.
    - HsCRP and inflammatory cytokines
- Questionnaires:
    - ✓ Impact of weight on quality of life-lite (IWQOL-Lite CT)
    - ✓ SF-36 physical functioning score
    - ✓ Power of food questionnaire
    - ✓ EuroQol-5D (EQ-5D) [28, 29]
- Concomitant medications.
- Adverse event (AE) review.
- Review of:
    - ✓ Drug diary

Visit 24: End-of-treatment assessment (Week 68 +/- 7 days)

The following assessments will be carried out following the last dose of semaglutide 3.0 mg/ml or 3.2 mg/ml /placebo:

- Physical examination.
- Vital signs (HR and BP).
- Urine pregnancy test for women of childbearing potential.
- Weight.
- BIA.
- ECG.
- Blood tests:
    - Haematology: full blood count.
    - Serum biochemistry: renal, liver and thyroid function, glucose, lipids, HbA1c (If baseline visit >7 days previously).
    - HsCRP and inflammatory cytokines
- Questionnaires:
    - ✓ Impact of weight on quality of life-lite (IWQOL-Lite CT)

- ✓ SF-36 physical functioning score
- ✓ EuroQol-5D (EQ-5D) [28, 29]
- ✓ Power of food questionnaire

- Concomitant medications.
- Adverse event (AE) review.
- Collection of:
  - ✓ Drug diary

Visits 25 and 26: Wash-out period and end of trial assessment (Week 70 and 74 +/- 7 days)

The final trial assessments will be carried out remotely.

Participants who discontinue treatment will be asked to remain enrolled in the trial and contribute their data and will be invited to continue attending trial visits as scheduled. Participants who withdraw before completion of the trial will be asked to attend for their end-of-trial visit (as soon as possible after withdrawal). Withdrawn participants will not be replaced.

The following will be assessed at the end of trial visit:

- Weight (using the scales provided to the participants).
- Concomitant medications.
- Adverse events (AE) review.

A schedule of all trial assessments and procedures is set-out in Appendix 1.

7.9 Discontinuation / Withdrawal Criteria

Reasons for discontinuing treatment may include:

- Intercurrent illness.
- Participant withdrawing consent.
- Persistent non-compliance to protocol requirements.
- Sustained increase in heart rate (≥110 bpm when rechecked later in the same clinic visit)
- Inability to tolerate semaglutide 3.0 mg/ml or 3.2 mg/ml at a dose greater than 1.0 mg, as evidenced by AE log.
- Pancreatitis diagnosed according to the Atlanta classification of two out of three of: severe acute abdominal pain often radiating to the back, serum amylase or lipase >3 ULN, characteristic findings on CT.MRI or US imaging
- Pregnancy, discontinuation of contraception or decision to try to conceive.
- Any alteration in the participant's condition, which justifies the discontinuation of treatment.

The decision to discontinue treatment will be recorded in the TMF, eCRF and medical notes. Sponsor will be notified in writing. With respect to a clinician's decision to discontinue treatment, the reason for this must be recorded. When this occurs, the relevant clinician or nominee will need to assess the participant clinically within a week and arrange appropriate care. Every effort will be made to obtain the primary outcome data (i.e., %WL). Such participant's data should be included in the trial analysis. If the participant will still consent to research data collection at follow-up, this will continue as normal.

As participation in the trial is entirely voluntary, the participant may choose to discontinue trial treatment at any time without penalty or loss of benefits to which they would otherwise be entitled. Although not obliged to give a reason for discontinuing their trial treatment, a reasonable effort should be made to establish this reason, whilst remaining fully respectful of the participant's rights. Efforts will be made to continue to obtain follow-up data, with the permission of the participant. The investigator will ascertain whether consent is withdrawn from further trial treatment only or from both trial treatment and follow-up.

If a participant explicitly states, they do not wish to contribute further data to the trial their decision will be respected and recorded in the eCRF and medical notes. In this case participants remain within the trial for the purposes of follow-up for safety and/ or data analysis. If a participant is lost to follow-up, every effort should be made to contact the participant's GP to obtain information on the participant's status. Subjects withdrawn from trial will not be replaced.

7.10 Stopping Rules

The trial may be stopped before completion for the following reasons:

1. On the recommendation of the Data and Safety Monitoring Committee (DSMC).
2. On recommendation of the Sponsor and CI.

7.11 Storage and analysis of clinical samples

Urine: pregnancy test.

Blood: The following test will be carried out:

	**Test**	**Laboratory (Storage and Analyses)**
Haematology	Full blood count	UCLH
Serum Biochemistry	Pregnancy test (serum Beta hCG), renal, liver and thyroid function, lipids, glucose, HsCRP and HbA1c	UCLH
GLP-1 and inflammatory cytokines	GLP-1 and inflammatory cytokines	Centre for Obesity Research, UCL

Samples will be processed and stored according to our established protocols and lab SOPs. Samples will not be transferred to any party not identified in the protocol and will not be processed and/or transferred other than in accordance with the patient's consent. After ethical approval of the study has expired the samples will be disposed of in accordance with the Human Tissue Act 2004 and any amendments thereto.

It is the responsibility of the trial site to ensure that samples are appropriately labelled in accordance with the trial procedures to comply with the Data Protection Act 2018. Biological samples collected from participants as part of this trial will be transported, stored, accessed and processed in accordance with national legislation relating to the use and storage of human tissue for research purposes and such activities shall at least meet the requirements as set out in the 2004 Human Tissue Act and the 2006 Human Tissue (Scotland) Act.

7.12 End of trial

The expected duration of the trial is 26 months from recruitment of the first participant. The end of trial will be declared after the date of the last visit of the last participant and after all sample analysis has been completed.

The trial may be stopped before completion for the following reasons:

1. On the recommendation of the Data and Safety Monitoring Committee (DSMC).
2. On recommendation of the Sponsor and CI.

8 TRIAL TREATMENTS

8.1 Name and description of investigational medicinal product(s)

8.1.1 Semaglutide

Name:	Semaglutide 3.0 mg/ml or 3.2 mg/ml solution for injection in pre-filled pen
Composition:	Active IMP used from 18 November 2022 to 04 November 2024: Semaglutide 3.0 mg/ml solution for injection in pre-filled pen.<br><br>In order to blind the product, a clinic variant is supplied: The clinical pen is a 3 ml PDS290 pen-injector containing Semaglutide 3.0 mg/ml or placebo solution for subcutaneous use. The clinical pen has a drum scale of 1-80 in increments of 1. The dose steps cannot be provided in mg by the pen and must therefore be calculated in numbers of increments as stated in the table below:<br><br>Semaglutide dose - Value shown in dose counter:<br>0.24 mg - 8<br>0.5 mg - 17<br>1.0 mg - 34<br>1.7 mg - 57<br>2.4 mg - 80<br><br>Active IMP used from 05 November 2024 until end of trial: Semaglutide 3.2 mg/ml solution for injection in pre-filled pen

	(PDS290) for subcutaneous use.<br>There is a change in dose dial (value shown in dose counter) on the semaglutide 3.2 mg/ml solution pen. The dose steps cannot be provided in mg by the pen and must therefore be calculated in numbers of increments as stated in the table below:<br>Semaglutide dose - Value shown in dose counter:<br>0.25 mg - 8<br>0.5 mg - 16<br>1.0 mg - 31<br>1.7 mg - 53<br>2.4 mg - 75<br>Participants enrolled in the trial will administer either semaglutide 3.0 mg/ml or placebo until 04 November 2024. From 05 November 2024, participants who were randomised to semaglutide 3.0 mg/ml will then receive semaglutide 3.2 mg/ml until trial completion. The maximum dose of 2.4 mg will remain the same. Participants randomised to placebo will continue on placebo until trial completion.
Manufacturer:	Novo Nordisk

8.1.2 Placebo

Name:	Placebo
Composition	Disodium hydrogen phosphate dihydrate, Propylene glycol, Phenol, Water for injection, Sodium hydroxide, Hydrochloric acid.
Manufacturer:	Novo Nordisk

8.2 Regulatory status of the drug

The IMP Semaglutide 3.2 mg/mL subcutaneous injection at a dose of 2.4 mg per week has been granted marketing authorisation in the UK as of 24th September 2021 (PL number PLGB 04668/0433). An updated marketing authorisation was granted by the MHRA in the UK on the 10th May 2022 with PL number PLGB 04668/0440.

8.3 Drug storage and supply

The drug aspects of the trial will be delegated by the CI to a delegated Lead Pharmacist. UCLH pharmacy will be responsible for storage and dispensing of semaglutide 3.0 mg/ml or 3.2 mg/ml and placebo according to handling and storage instructions as reported in the Summary Of Drug Arrangements (SODA).

A log of all received, used, partly used and unused trial products will be kept. The trial products will be dispensed to each participant as required according to treatment group. Proper storage conditions (outlined below) will be available and the temperature will be evaluated and recorded at least every working day. No trial product should be dispensed to any person not enrolled in the trial.

Not in use: The semaglutide 3.0 mg/ml or 3.2 mg/ml / placebo will be stored according to manufacturer's instructions as reported on the label.

In use: After first opening the semaglutide 3.0 mg/ml or 3.2 mg/ml / placebo pre-filled pen should be stored according to manufacturer's instructions as reported on the label.

Drug destruction will be conducted, once authorised by the sponsor and in accordance with local practice or returned to Novo Nordisk and this will be documented in the drug destruction log in the hospital pharmacy file.

Detailed instructions are contained in the summary of drug arrangements.

8.4 Labelling of Investigational Medicinal Product

Labelling of the IMPs will be completed in accordance with the relevant GMP guidelines (Eudralex Volume 4 Annex 13).

8.5 Dosage schedules

Dose escalation schedule of semaglutide 3.0 mg/ml or 3.2 mg/ml / placebo is as following:

	**Semaglutide dose - Value shown in dose counter* (3.0 mg/ml pre-filled pen)**	**Semaglutide dose – Value shown in dose counter (3.2 mg/ml pre-filled pen)**	**Weeks**
**Dose escalation 16 weeks**	0.24 mg - 8	0.25 mg - 8	1-4
	0.5 mg - 17	0.5 mg - 16	5-8
	1.0 mg - 34	1.0 mg - 31	9-12
	1.7 mg - 57	1.7 mg - 53	13-16
**Maintenance dose**	2.4 mg – 80	2.4 mg - 75	From week 17 onwards

	*In order to blind the product, a clinic variant is supplied: The clinical pen is a 3 ml PDS290 pen-injector containing Semaglutide 3.0 mg/ml or 3.2 mg/ml or placebo solution for subcutaneous use.<br>The clinical pen has a drum scale of 1-80 in increments of 1. The dose steps cannot be provided in mg by the pen and must therefore be calculated in numbers of increments as stated in the table above.

At the end of the baseline visit, after randomisation, participants will be trained and instructed to inject semaglutide 3.0 mg/ml or 3.2 mg/ml /placebo once weekly at any time of day, without regard to the timing of meals, but ideally establishing a routine for the same time each week. Semaglutide 3.0 mg/ml or 3.2 mg/ml or placebo can be injected subcutaneously in the abdomen, thigh, or upper arm. The injection site and timing can be changed without dose adjustment, but it is important to try and stick to similar timing each week. A Directions for Use (DFU) for the device will be provided by Novo Nordisk A/S and the DFU will be handed out to subject at first dispensing visit and subject will be trained according to this DFU supplied separately from the trial product package. This will be documented in the medical records.

If a single dose of trial product is missed, it should be administered as soon as noticed, provided the time to the next scheduled dose is at least 2 days (48 hours). If a dose is missed and the next scheduled dose is less than 2 days (48 hours) away, the subject should not administer a dose until the next scheduled dose. A missed dose should not affect the scheduled dosing day of the week. This information will be given to the participants in written format in the DFU and also verbally. Participants will record dosage taken and missed in the patient diary.

Participants will be provided with semaglutide 3.0 mg/ml or 3.2 mg/ml or placebo via pharmacy in pre-filled pens. Dispensing visits will coincide with site visits and treatment product will be dispensed at Visit 2, Visit 6, Visit 10, Visit 15 and Visit 20. Participants will be provided with treatment product to last until their following site visits from the clinical trials pharmacy at UCLH.

8.6 Dosage modifications

If participants are unable to tolerate an increased dose during dose escalation, delaying dose escalation for approximately one additional week will be considered. If the full dose drug of 2.4 mg is not tolerated participants will be allowed to continue at a reduced dose of 1.7 mg. Semaglutide 3.0 mg/ml or 3.2 mg/ml / placebo will be discontinued, however, if a participant cannot tolerate a dose of 1.7 mg. Participants who discontinue treatment will be able to restart if clinically indicated and will have to follow the 16-week escalation protocol. Any dosage changes will be logged in a drug diary by participants and this will be reviewed at each trial visits.

8.7 Known drug reactions and interaction with other therapies

Summary of known and potential risks and benefits of the IMP can be found in the ‘SmPC for Semaglutide subcutaneous administration for weight management’.

8.8 Concomitant Medication

Concomitant medications will be recorded in the participant's medical records/CRF/eCRF and reviewed at each trial visit. For participants with T2D taking anti-diabetic medications, such as sulfonylureas, medication review will be throughout the trial period. Glucose monitoring will be commenced one week prior to randomisation and continued throughout the study period and the anti-diabetic medication will be adjusted accordingly. Careful monitoring of symptoms of hypoglycaemia will be carried out throughout the trial. The use of insulin, DPPIV-inhibitors, GLP-R agonist or any medication known to cause weight gain or weight loss will not be permitted in this study. Also, people who are on these medications will not be recruited into the trial.

Semaglutide 3.0 mg/ml or 3.2 mg/ml causes a delay in gastric emptying, and thereby has the potential to impact the absorption of concomitantly administered oral medications. Further details of interaction with other medicinal products and other forms of interaction with the IMP can be found in the 'Summary of Product Characteristics (SmPC)' for Semaglutide subcutaneous administration for weight management'.

8.9 Trial Restrictions

<u>Female participants</u>

Females of childbearing potential are eligible to participate if they agree to use a highly effective contraception method for the duration of the trial and until 2 months after treatment discontinuation.

Women are considered of childbearing potential following menarche and until becoming post-menopausal unless permanently sterile. Women are considered permanently sterile if they have had documented hysterectomy, bilateral salpingectomy or bilateral oophorectomy. Postmenopausal state is defined as no menses for 12 months without any medical cause. A high Follicle Stimulating Hormone (FSH) level in the postmenopausal range may be used to confirm a postmenopausal state in women not using hormonal contraception or Hormonal Replacement Therapy (HRT). However, in the absence of 12 months of amenorrhoea, a single FSH measurement is insufficient.

Highly effective contraceptive methods include:

- Combine (oestrogen and progesterone containing) hormonal contraception associated with inhibition of ovulation:
  - Oral
  - Intravaginal
  - Transdermal
- Progesterone-only hormonal contraception associated with inhibition of ovulation:
  - Oral
  - Injectable
  - Implantable
- Intrauterine device.
- Intrauterine hormone-releasing system.
- Bilateral tubal occlusion.
- Vasectomised partner.
- True sexual abstinence (refraining from sexual intercourse – only acceptable when this is in line with the preferred and usual lifestyle of the subject).

<u>Male participants</u>

The effect of semaglutide on fertility in humans is unknown. Male participants with partners of child bearing potential must use barrier methods of contraception for the duration of the trial until 2 months after treatment discontinuation.

8.10 Assessment of compliance with treatment

Compliance includes both adherences to IMP and Protocol study procedures. Subjects will be provided with a drug diary to record their home-dosing. Any missed dosage will be logged in the drug diary. This will be reviewed at each trial visit. Non-compliance will be documented by the investigator in the medical notes and reported to the sponsor. Persistent non-compliance may lead to subject withdrawal from the study.

8.11 Name and description of each Non-Investigational Medicinal Product (NIMP)

Name:	Placebo
Composition	Provided in cross referral letter from manufacturer for previously submitted trial EudraCT: 2019-004594-44
Manufacturer:	Novo Nordisk

9 PHARMACOVIGILANCE

Collection, recording and reporting of adverse events to the sponsor will be completed according to the sponsor's SOP for the Recording, Management and Reporting of Adverse Events by Investigators (JRO/INV/S05).

9.1 Definitions

Term	Definition
**Adverse Event (AE)**	Any untoward medical occurrence in a subject to whom a medicinal product is administered and which does not necessarily have a causal relationship with this treatment.<br>*Therefore an AE can be any unfavourable or unintended change in the structure (signs), function (symptoms) or chemistry (laboratory data) in a subject to whom an IMP has been administered, including occurrences which are not necessarily caused by or related to that product.*
**Adverse Reaction (AR)**	A response to a medicinal product which is noxious and unintended and which occurs at doses normally used in man for the prophylaxis, diagnosis or therapy of disease or for the restoration, correction or modification of physiological function.<br>*This definition implies a reasonable possibility of a causal relationship between the event and the IMP. This means that there are facts (evidence) or arguments to suggest a causal relationship.*<br>*This definition also covers medication errors and uses outside what is foreseen in the protocol, including misuse and abuse of the product.*

**Serious Adverse Event (SAE)**<br><br>**or**<br><br>**Serious Adverse Reaction (SAR)**	Any adverse event or adverse reaction in a trial subject that:<br>a) requires inpatient hospitalisation or prolongation of existing hospitalisation;<br>*Note: hospitalisation is defined as an inpatient admission, regardless of length of stay, even if the hospitalisation is a precautionary measure for continued observation. Therefore, participants do not need to be hospitalised overnight to meet the hospitalisation criteria. Hospitalisation (including hospitalisation for an elective procedure) for a pre-existing condition (prior to study entry) which has not worsened does not constitute a serious experience*<br>b) results in persistent or significant disability or incapacity;<br>*Note: substantial disruption of one's ability to conduct normal life functions*<br>c) results in a congenital anomaly or birth defect;<br>*Note: in offspring of subjects or their partners taking the IMP regardless of time of diagnosis*<br>d) is life threatening; or<br>*Note: places the subject, in the view of the investigator, at immediate risk of death from the experience as it occurred, this does not include an adverse experience that, had it occurred in a more severe form, might have caused death;*<br>e) results in death<br>Some medical events may jeopardise the subject or may require an intervention to prevent one of the above characteristics/consequences. Such **important medical events** should also be considered as serious.<br>The term "**severe**" is often used to describe the intensity of an event or reaction (e.g. mild, moderate or severe) and should not be confused or interchanged with the term "**serious**".<br>Suspicion of transmission of infectious agents must always be considered an SAE.<br>Serious adverse reaction (SAR) is an Adverse event which fulfils both the criteria for a Serious Adverse event and the criteria for an Adverse Reaction.
**Suspected Unexpected Serious Adverse Reaction (SUSAR)**	A serious adverse reaction, the nature, severity or outcome of which is not consistent with the Reference Safety Information.
**Reference Safety Information (RSI)**	A list of medical events that defines which reactions are expected for the IMP being administered to clinical trial subjects, and so do not require expedited reporting to the Competent Authority. It is contained in a specific section in the Summary of product characteristics (SmPC) or the Investigator Brochure (IB).

9.2 Recording and Reporting Adverse Events

Adverse events will be recorded by the investigator at each visit post-baseline. AEs will be documented in the medical notes in the first instance, and CRF following consent.

When recording an adverse event, clinical symptoms and a simple, brief description of the event, including dates as appropriate, should be reported. Clinically significant abnormalities in the results of objective tests (e.g. laboratory variables) will also be recorded as AEs in the medical notes and the eCRF. Where possible, a diagnosis rather than a list of symptoms should be recorded. If a diagnosis has not been made, then each symptom should be listed individually.

All adverse events will be recorded until 6 weeks after the end of treatment. At the end-of-trial assessment (i.e. six weeks after participant's end-of-treatment visit) a trial investigator will call the participants to collect any adverse event up until that point and this will be recorded in the medical notes and CRF following the same procedure as outlined above.

9.3 Assessing Adverse Events

Each adverse event will be assessed for severity, causality and seriousness as described below.

9.3.1 Severity

The medical assessment of severity will be determined regardless of causality at each assessment. The intensity will be determined by using the following definitions:

Category	Definition
**Mild**	The adverse event does not interfere with the participant's daily routine and does not require intervention; it causes slight discomfort.
**Moderate**	The adverse event interferes with some aspects of the participant's routine, or requires intervention, but is not damaging to health; it causes moderate discomfort.
**Severe**	The adverse event results in alteration, discomfort or disability which is clearly damaging to health.

9.3.2 Causality

The assessment of relationship of adverse events to the administration of IMP must be made by the investigator (or delegated medically qualified person). It is based on clinical judgement using all available information at the time of the completion of the case report form.

The following categories will be used to define the causality of the adverse event:

Category	Definition
**Related**	A causal relationship between an IMP/investigational treatment and an adverse event is at least a **reasonable possibility**, i.e., the relationship cannot be ruled out.

**Not Related**	There is **no reasonable possibility** of a causal relationship between an IMP/investigational treatment and an adverse event.

9.3.3 Seriousness

All events are assessed for seriousness as defined for an SAE in Section 9.1 - Definitions.

9.4 Recording and reporting of Serious Adverse Events

All **Serious Adverse Reactions (SAEs)** occurring from the time of written informed consent until 6 weeks after the participant's EOS visit must be recorded in the medical records, the CRF and SAE Reporting Form and reported to the Sponsor **within 24 hours** of the research staff becoming aware of the event.

The Investigator or designated individual will complete the Sponsor's trial specific SAE Reporting Form and email it to the Sponsor at **SAE@ucl.ac.uk**. The Investigator will respond to any SAE queries raised by the Sponsor as soon as possible.

Completed SAE Reporting Forms must be sent to the Sponsor within 24 hours of becoming aware of the event

Email SAE Forms to: SAE@ucl.ac.uk

Any change of condition or other follow-up information should be emailed to the Sponsor, on an SAE Reporting Form (clearly marked as follow-up) as soon as it is available or at least within 24 hours of the information becoming available.

Events will be followed up until the event has resolved or a final outcome has been reached. SAE follow-up should continue after completion of protocol treatment and/or trial follow-up if necessary. Any SAR will need to be reported to the Sponsor irrespective of how long after IMP administration the reaction has occurred until resolved. The Sponsor should report all SARs and pregnancy reports related to Novo Nordisk (NN) Product to the local NN affiliate safety department. The submission to NN must be within day 15 from the Sponsor's first knowledge about a valid case.

A listing of SAEs from the trial database will be reported to Sponsor at least once or twice per year in liaison with JRO Pharmacovigilance Manager.

9.4.1 Serious Adverse Events which do not require reporting to Sponsor

The following events do not require immediate reporting to the sponsor as SAEs, however they will still be recorded in the participant's medical records.

Hospitalisation for:

- Routine treatment or monitoring of the studied indication not associated with any deterioration in condition.
- Any admission to hospital or other institution for general care where there was no deterioration in condition.

- Treatment on an emergency, outpatient basis for an event not fulfilling any of the definitions of serious as given above and not resulting in hospital admission.

9.4.2 SUSAR Reporting

All SAEs assigned by the PI or delegate as suspected to be related to IMP-treatment (SARs) will be assessed for expectedness against the current approved Reference Safety Information (RSI) for the trial by the Sponsor.

The following categories will be used to define the expectedness of the SAR:

Category	Definition
Expected	An adverse event which is consistent with the information about the IMP listed in the current approved Reference Safety Information (RSI) for the trial.
Unexpected	An adverse event which is not consistent with the information about the IMP listed in the current approved Reference Safety Information (RSI) for the trial.

All SARs assessed as unexpected will be classified as SUSARs and will be subject to expedited reporting to the MHRA and REC.

The RSI to be used to assess expectedness against the IMP is:

Wegovy (semaglutide) Summary of Product Characteristics (SmPC), section 4.8: Undesirable Effects

The sponsor will notify the main REC and MHRA of all SUSARs within the expedited reporting timescales. Any reports sent to the MHRA will also be forward to NN. SUSARs that are fatal or life-threatening must be notified to the MHRA and REC within 7 days after sponsor awareness. Other SUSARs must be reported to the REC and MHRA within 15 days after the sponsor awareness.

9.4.3 Unblinding for the submission of SUSAR reports

A representative of the Sponsor will be authorised to access the code break system for the purposes of unbinding for the submission of a SUSAR. If the participant has received active treatment, the sponsor will submit the SUSAR report to the MHRA and REC.

SUSAR information will be disseminated to Investigators at site(s) and will remain blinded, regardless of whether the participant received active treatment or not. The unblinded information will not be forwarded to the trial team and will be kept in the JRO sponsor file.

Unblinding for the submission of SUSAR reports:

The following procedure will be used to unblind for the submission of a SUSAR report to the regulatory agencies:

- A member of the JRO Sponsor's office will be authorised to access the code break system for the purposes of unblinding for the submission of a SUSAR.
- On receipt of the treatment allocation, the Sponsor will provide the unblinded information on the e-SUSAR website form.

- Unblinded information in the SUSAR reports will not be forwarded to the trial team and kept in the JRO sponsor file.
- SUSAR reports will be disseminated to Investigators at site(s) but will remain blinded.

9.4.4 Flow Chart for Adverse Event Recording

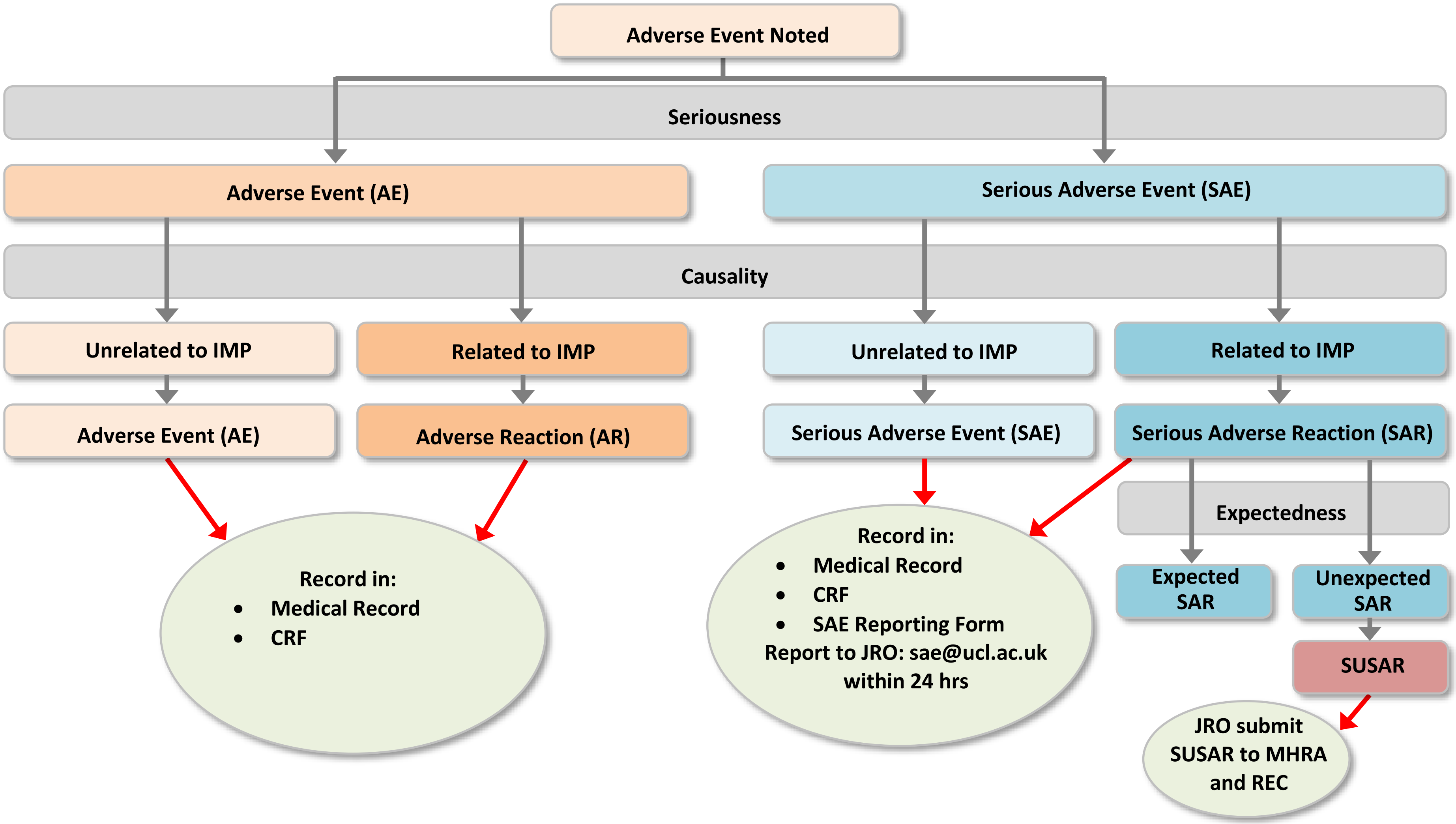

9.5 Pregnancy Reporting

If a female participant or the female partner of a male participant becomes pregnant at any point during the trial, a completed trial specific Pregnancy Reporting Form will be emailed to the Sponsor at **SAE@ucl.ac.uk**, within 24 hours of the Investigator becoming aware of the event in line with the Sponsors SOP (JRO/INV/S05). The Chief or Principal Investigator will respond to any queries raised by the sponsor as soon as possible.

Completed Pregnancy Reporting Forms must be sent to the Sponsor within 24 hours of becoming aware of the event

Email Pregnancy Forms to: SAE@ucl.ac.uk

The Sponsor must be kept informed of any new developments involving the pregnancy through the completion of a follow-up Pregnancy Reporting Form. Any pregnancy that occurs in a female trial subject during a clinical trial should be followed to termination or to term.

Consent to report information regarding the pregnancy must be obtained from the pregnant participant or partner. A trial-specific pregnancy monitoring information sheet and informed consent form for trial participants and the partners of trial participants must be used for this purpose.

With consent additional information regarding the pregnancy will be collected and reported to the Sponsor, the Sponsor will advise on the length of follow up of the pregnancy / child on a case by case basis. Such individual reports shall be sent to Novo Nordisk within the same timeline as if they were reportable to regulatory authorities.

9.6 Overdose

In the event of an accidental or intentional overdose by a trial participant, the investigators will immediately inform the CI and the Sponsor's office. Overdose can be observed from the drug diary or reported by participants. The deviation log will be completed and the medical notes and eCRF will be updated to reflect this information. In the event that the overdose is associated with an S/AE, the two events will be linked. In the event of an AE associated with an overdose, a SAE report form will be completed detailing the AE and the overdose details. The investigators will justify whether patients should remain or withdrawn from the trial. Resultant symptoms will be treated as per routine clinical care.

9.7 Reporting Urgent Safety Measures

If any urgent safety measures are taken the CI/Sponsor shall immediately and in any event no later than 3 days from the date the measures are taken, give written notice to the MHRA and the relevant Research Ethics Committee (REC) and the Sponsor of the measures taken and the circumstances giving rise to those measures. The clinical trials safety reporting will refer to the following website:

http://www.mhra.gov.uk/Howweregulate/Medicines/Licensingofmedicines/Clinicaltrials/Safetyreporting-SUSARSandASRs/index.htm

9.8 Development Safety Update Reports

The Sponsor will provide the MHRA and REC with Development Safety Update Reports (DSUR) which will be written by the Sponsor's office in conjunction with the trial team. The report will be submitted within 60 days of the Developmental International Birth Date (DIBD) of the trial each year until the trial is declared ended.

9.9 Responsibilities

Principal Investigator (PI) / delegate:

Checking for AEs and ARs when participants attend for treatment / follow-up.

1. Using medical judgement in assigning seriousness and causality.
2. Ensuring that all SAEs are recorded and reported to the sponsor within 24 hours of becoming aware of the event and provide further follow-up information as soon as available. Ensuring that SAEs are chased with Sponsor if a record of receipt is not received within 3 working days of initial reporting.
3. Ensuring that AEs and ARs are recorded and reported to the sponsor in line with the requirements of the protocol.

Chief Investigator (CI) / delegate:

All of the above responsibilities of a PI, and in addition:

1. Clinical oversight of the safety of participants participating in the trial, including an ongoing review of the risk / benefit.
2. Using medical judgement in assigning the SAEs seriousness and causality where it has not been possible to obtain local medical assessment.
3. Review of all SUSARs.
4. Review of specific SAEs and SARs in accordance with the trial risk assessment and protocol.
5. Reporting safety information to the independent oversight committees identified for the trial (Data Monitoring Committee (DMC) and Trial Steering Committee (TSC)
6. Reviewing and contributing to the annual Development Safety Update Report (DSUR).

Sponsor:

1. Data collection and verification of SAEs, SARs and SUSARs according to the trial protocol onto a database.
2. Reporting safety information to the CI or delegate for the ongoing assessment of the risk / benefit.
3. Expedited reporting of SUSARs to the Competent Authority (MHRA in UK) and REC within required timelines.
4. Notifying Investigators of SUSARs that occur within the trial.
5. The unblinding of a participant for the purpose of expedited SUSAR reporting
6. Checking for and notifying PIs of updates to the Reference Safety Information for the trial.
7. Preparing the DSUR in collaboration with the CI and ensuring timely submission to the MHRA and REC (within 60 calendar days).

Trial Steering Committee (TSC):

The role of the TSC is to provide overall supervision of the trial. The TSC will review the recommendations of the Data Safety Monitoring Committee (DSMC) and, on consideration of this information, recommend any appropriate amendment/actions for the trial as necessary. The TSC will act on behalf of the funders and Sponsor. The TSC will be chaired by an external academic member with experience in leading clinical trial and will include a lay member.

Data Monitoring Committee (DMC):

In accordance with the Trial Terms of Reference for the DMC, periodically reviewing overall safety data to determine patterns and trends of events, or to identify safety issues, which would not be apparent on an individual case basis.

The role of the DSMC is to provide independent advice on data and safety aspects of the trial. The committee will review data and safety reports blindly. Meetings will be held on regular basis according to the DMSC charter to address any issues. We will aim to include an external layperson, an external bariatric surgical consultant, an external statistician and an external specialist bariatric physician. We do not anticipate any major safety concerns with this study. The DSMC will be independent of the TSC and advisory to the TSC and can recommend any of the following options:

1. continue according to protocol.
2. continue but with recommended modifications.
3. termination of the trial.

10 STATISTICS AND DATA ANALYSIS

10.1 Outcomes

10.1.1 Primary Outcomes

The primary outcome of this trial is %WL from the baseline visit to 68 weeks of treatment. Percentage weight loss will be calculated using the following formula: %WL = [(weight at the baseline visit–weight at the end of the 68-week treatment period)/ weight at the baseline visit] x 100, measured at the end of treatment.

10.1.2 Secondary Outcomes

The secondary outcomes of this trial are:

1. The percentage of participants receiving subcutaneous semaglutide 3.0 mg/ml or 3.2 mg/ml at a dose of 2.4 mg per week versus placebo who after 68 weeks achieve:
   - A body weight reduction ≥10%
   - A body weight reduction ≥15%
   - A body weight reduction ≥20%

2. The effect of 68 weeks of subcutaneous semaglutide 3.0 mg/ml or 3.2 mg/ml at a dose of 2.4 mg per week versus placebo administration upon glycaemia, pre-diabetes and T2D by comparing:

- Change in circulating HbA1c levels.
- Change in circulating HbA1c levels in participants with pre-diabetes at baseline.

- Change in circulating HbA1c levels in participants with T2D at baseline.
- The number of pharmacological agents required for the management of T2D in participants with pre-existing T2D at baseline.

3. The effect of 68 weeks of subcutaneous semaglutide 3.0 mg/ml or 3.2 mg/ml at a dose of 2.4 mg per week versus placebo administration upon BP and hypertension by comparing:

- Systolic and diastolic BP.
- Systolic and diastolic BP in participants with pre-existing hypertension.
- The number of pharmacological agents required for the management of hypertension in participants with pre-existing hypertension.

4. The effect of 68 weeks of subcutaneous semaglutide 3.0 mg/ml or 3.2 mg/ml at a dose of 2.4 mg per week versus placebo administration upon:
    - Change in lipids and inflammatory markers (HsCRP and inflammatory cytokines).
    - Change in food cravings.
    - Change in HRQoL measures.

5. The relationship between GLP-1 levels at baseline and %WL at 68 weeks.

10.2 Sample size justification

A sample size calculation was conducted using the 24-week primary ITT analysis from our BARI-OPTIMISE trial with liraglutide 3.0mg versus placebo in people with poor weight loss following bariatric surgery and the results of the Davies et al. multi-centre trial of Semaglutide 3.0 mg/ml in adults with overweight or obesity, and type 2 diabetes. Assuming a SD for %WL of 4.0, dropout rate of 10%, and 1% critical significance level, 62 patients (31 per group) will provide at least 95% power to detect an estimated difference of 10%WL with a 95% CI no wider than 8.0 to 12.0. The recruitment target was set at 35 participants per group. We anticipate screening between 80 and 100 patients with less than 20% weight loss at one year or more post-surgery. We will consent, recruit and randomise the first 70 patients who fulfil the inclusion and exclusion criteria.

10.3 Planned Recruitment Rate

Participants will be recruited from UCLH. These units have been established for >5 years and undertake >800 primary GB and/or SG per year and follow up their patients indefinitely. At least 20% of patients undergoing GB and SG experience poor weight loss providing approximately 160 potentially eligible patients. We anticipate recruiting at least 15 participants per month and to complete recruitment within a 5-month period.

10.4 Randomisation Methods

Participants will be deemed enrolled in the trial once informed consent has been obtained (by signing the study consent form) and screening data collected. The screening assessments will take part after informed consent has been obtained. Once eligibility is confirmed, participants will be invited to attend for the baseline visit, where baseline data will be collected and randomisation will be performed. The randomisation procedure will be remotely carried out through an online randomisation service, with provision to enable unblinding if required.

Subjects will be randomly assigned in a 1:1 ratio to receive either semaglutide 2.4 mg or placebo, stratified for sex, type of surgical procedure and T2D status (stratified block randomisation with varying block sizes). The investigator will provide participant's initials, date of birth and stratification information before a randomisation code can be generated for each participant. Both participants and investigators will be blinded to study-group assignments. Once randomisation has been performed, no group allocation will be disclosed. A randomisation notification email will be sent to the pharmacist reporting the participant randomisation code; this randomisation code will reveal group allocation when identified in the code list. Only the trial coordinator and the trial pharmacist will have access to the code list. The blinding of the trial will be maintained throughout the trial until all data entry and processing are complete, the database has been locked and data analysis performed. Participants will be given a 24-hour Contact Card for emergency unblinding if required, medical support, or for any enquiries they have throughout the study period. For details on unblinding procedure refer to the trial Randomisation, Unblinding and Code break SOP.

10.5 Statistical Analysis Plan

A detailed analysis plan will be drawn up prior to database lock or seeing any data.

10.5.1 Summary of baseline data and flow of participants

A CONSORT diagram will be presented. Patient characteristics will be described using means (SDs) or medians (interquartile range) for continuous measures and proportions for categorical measures. These values will be presented by randomisation group.

10.5.2 Primary Outcome Analysis

The mean difference in %WL at 68 weeks between the groups will be analysed using linear regression, adjusting for stratification variables and any baseline variables which are not balanced between the groups. Mean difference in %WL will be reported with 95% confidence interval. The assumptions of the model will be checked, and a suitable transformation/non-parametric method will be used where the assumptions are not met. All available data will be analysed as randomised. Bias due to missing data will be investigated and dealt as appropriate.

10.5.3 Secondary Outcome Analysis

The results of the secondary analysis will be treated as exploratory. Continuous outcomes will be analysed using separate linear regression models, adjusting for stratification variables and any baseline variables which are not balanced between the groups. Mean differences in each outcome will be reported with 95% confidence intervals. The corresponding analyses for binary and count outcomes will use binary logistic and zero-rated Poisson regressions respectively instead of linear regressions. The assumptions of each model will be checked, and a suitable transformation/non-parametric method will be used where the assumptions are not met.

To evaluate the economic impact of the trial intervention, we will calculate the cost-effectiveness from an NHS and personal social services (PSS) perspective, relative to usual care. The analysis will be based on per-participant intervention costs, and NHS/PSS resource use and HRQoL assessed retrospectively in the trial between baseline

and end of the 68-week treatment period. We will calculate the incremental cost per quality-adjusted life year (QALY) gained for the within-trial period. We will run deterministic and probabilistic sensitivity analyses.

10.6 Interim analysis and criteria for the premature termination of the trial

No interim analysis is planned.

The trial may be stopped before completion for the following reasons:

1. On the recommendation of the Data and Safety Monitoring Committee (DSMC).
2. On recommendation of the Sponsor and CI.

10.7 Participant Population

Study population: patients, 1 year or more following primary GB or primary SG, with poor weight-loss response (<20% WL) will be invited to participate. There will be no waivers to the inclusion and exclusion criteria.

10.8 Procedure(s) to account for missing or spurious data

Trial staff will offer flexibility to trial participants as allowed within the study protocol to allow participants to attend follow-up visits. In the event of any missed visits or visits undertaken outside the windows outlined in this protocol, the reason will be clearly documented, and this will be recorded as a protocol deviation.

All available data at week 68 will be used in the analysis and missing values at week 68 will be imputed and the endpoints will be derived from the imputed values. The imputation approach to be used will be decided as deemed appropriate by the trial statistician.

10.9 Other Statistical Considerations.

Any deviations from the original statistical plan will be described and justified in the protocol and/or in the final report, as appropriate.

10.10 Economic Evaluation

To evaluate the economic impact of the trial intervention, we will calculate the cost-effectiveness from an NHS and personal social services (PSS) perspective, relative to usual care. The analysis will be based on per-participant intervention costs, and NHS/PSS resource use and HRQoL assessed retrospectively in the trial between baseline and end of the 68-week treatment period. We will calculate the incremental cost per quality-adjusted life year (QALY) gained for the within-trial period. We will run deterministic and probabilistic sensitivity analyses.

11 DATA MANAGEMENT

11.1 Data collection tools and source document identification

Data will be collected using CRF. Source data will be accurately transcribed on to the CRF. Examples of source documents are medical records that included laboratory and other clinical reports. A source document list will

be implemented prior to the start of the trial to identify data to be recorded firstly into source documents, such as medical notes and then transcribed onto the CRF.

The database and CRF will be designed in conjunction so that data captured are complete, accurate, reliable and consistent. The database will be built using Red Pill, Sealed Envelope's online database application for electronic data capture (EDC). The delegation log will identify all those personnel with responsibility for data handling including those who have access to the trial database.

The Investigators are responsible for ensuring the accuracy of all the data entered in the eCRFs. Source data and CRF data will be checked as being accurate, complete, reliable and consistent before it is entered onto the database by individuals delegated the responsibility outlined in the delegation log. This will include

- Screening data verification prior to randomisation to ensure patients fulfil the inclusion/exclusion criteria.
- Ensuring that all AEs are reported and recorded.
- Queries relating to eCRF entries are corrected within a suitable time frame.
- CRF will be checked for errors before being deemed as complete and this process will be documented.

A Data Manager will ensure that appropriate corrections, additions, or deletions are made, dated, explained and initialled by the Investigator or by a member of the Investigator's trial staff who is authorised to initial CRF changes for the Investigator. A Data and Safety Monitoring Plan (DSMP) will outline trial procedures to be undertaken in ensuring data and safety monitoring throughout the lifespan of the Trial.

All CRFs will be completed and signed by staff that are listed on the staff delegation log and authorised by the CI to perform this duty. The CI will be responsible for the accuracy of all data reported in the CRF.

11.2 Data handling and record keeping

All data will be collected from participants in accordance with the participant consent form, PIS and study protocol. The data generated from this study will be appropriately sent to an appointed Data Manager and Trial Statistician for processing and statistical analysis and the Sponsor will act as the data controller of such data for the study. Data will be processed, stored and disposed of in accordance with all applicable legal and regulatory requirements including the UK GDPR, the DPA 2018 and any amendments thereto. CRFs and questionnaires will be stored in locked filing cabinets controlled by the CI. The Sealed Envelope RedPill eCFR database is password protected and only approved study personnel will be given access. The site specific access database will be stored separately within the Data Safe Haven and only approved study personnel will be given access. Information regarding database backup and storage will be documented. The data will not be transferred to any party not identified in this protocol and will not be processed and/or transferred other than in accordance with the participant's consent.

Data analysis will be undertaken by members of the research team and the designated study statistician and health economist. Data transfer between these parties will only occur in an anonymised form through password-protected files and secure data transfer platform within the Data Safe Haven server of UCL.

A trial specific data management SOP will be in place for the trial. This will contain details of the software to be used for the database, the process of database design, database validation, data entry, data quality checks, data queries, data security, database lock and data transfer.

Where data are transferred electronically this will be in accordance with the UK Data Protection Act 2018 as well as UCL Information Security Policy and Trust Information Governance Policy. There will be a documented record of data transfer and measures in place for the recovery of original information after transfer.

11.3 Access to Data

The investigator/ institution will permit trial-related monitoring, audits, REC review, and regulatory inspection(s), providing direct access to the source data/documents. Trial participants are informed of this during the informed consent discussion. Participants will consent to provide access to their medical notes.

11.4 Archiving

Archiving will be authorised by the Sponsor following submission of the end of study report. The CI will be responsible for the secure archiving of essential trial documents and the trial database as per their trust policy. UCL and each participating sites recognise that there is an obligation to archive study-related documents at the end of the study (as such end is defined within this protocol). The CI confirms that he/she will archive the study master file at The Centre for Obesity Research, Rayne Building, UCL for the period stipulated in the protocol and in line with all relevant legal and statutory requirements. The Principal Investigator at each participating site agrees to archive his/her respective site's study documents for 25 years and in line with all relevant legal and statutory requirements. Study participants are informed of this during the informed consent process. The Sponsor will notify the site when study documentation can be archived. When all essential documents are ready to archive, the CI will contact the UCL Records Office by email (records.office@ucl.ac.uk) to arrange ongoing secure storage. Essential documents are those which enable both the conduct of the trial and the quality of the data produced to be evaluated and show whether the trial complied with all applicable regulatory requirements. All archived documents will continue to be available for inspection by appropriate authorities upon request. Destruction of essential documents will require authorisation from the Sponsor.

12 OVERSIGHT COMMITTEES

12.1 Trial Management Group (TMG)

The TMG will include the CI, Trial Coordinator, Data Manager and co-investigators. The TMG will be responsible for maintaining the overseeing the trial. The TMG will meet before commencement of the trial and for scheduled meetings every 3 months throughout the trial, as well as when required during the study. The TMG will approve the final trial protocol, any subsequent amendments and the eCRF. A trial specific monitoring plan will be established for the study. The trial will be monitored with the agreed plan.

12.2 Trial Steering Committee (TSC)

The role of the TSC is to provide overall supervision of the trial. The TSC will review the recommendations of the Data Safety Monitoring Committee (DSMC) and, on consideration of this information, recommend any appropriate amendment/actions for the trial as necessary. The TSC will act on behalf of the funders and Sponsor. The TSC will be chaired by an external academic member with experience in leading clinical trial and will include a lay member.

12.3 Data and Safety Monitoring Committee (DSMC)

The role of the DSMC is to provide independent advice on data and safety aspects of the trial. The committee will review data and safety reports blindly. Meetings will be held on regular basis according to the DMSC charter to address any issues. We will aim to include an external layperson, an external bariatric surgical consultant, an external statistician and an external specialist bariatric physician. We do not anticipate any major safety concerns with this study. The DSMC will be independent of the TSC and advisory to the TSC and can recommend any of the following options:

1. continue according to protocol.
2. continue but with recommended modifications.
3. termination of the trial.

13 MONITORING, AUDIT & INSPECTION

The sponsor will determine the appropriate level and nature of monitoring required for the trial. Risk will be assessed on an ongoing basis and adjustments made accordingly. The degree of monitoring will be proportionate to the objective, purpose, phase, design, size, complexity, blinding, endpoints and risks associated with the trial. A trial specific oversight and monitoring plan will be established for studies. The trial will be monitored in accordance with the agreed plan.

14 ETHICAL AND REGULATORY CONSIDERATIONS

14.1 Research Ethics Committee (REC) / MHRA review & reports

The trial will be conducted in compliance with the principles of the Declaration of Helsinki (1996), the principles of International Conference on Harmonisation Good Clinical Practice (ICH GCP) and in accordance with all applicable regulatory requirements including but not limited to the Research Governance Framework and the Medicines for Human Use (Clinical Trial) Regulations 2004, as amended in 2006 and any subsequent amendments. NHS management permission will be obtained from the UCLH JRO who will also undertake data monitoring and provide Sponsorship for the trial. Ethical approval for this study will be obtained from the Health Research Authority (via the Integrated Research Application System) including review by an NHS Research Ethics Committee (REC) and from the Medicines and Healthcare products Regulatory Agency (MHRA) for Clinical Trial Authorisation. The CI will submit a final report at conclusion of the trial to the REC and the MHRA.

The Sponsor will ensure that trial protocol, participant information sheet, consent form, GP letter and submitted supporting documents have been approved by the appropriate REC, prior to any participant recruitment. The protocol, all supporting documents and agreed documents, will be documented and submitted for ethical and regulatory approval as required. Amendments will not be implemented prior to receipt of the required approval (s).

Before any NHS site may be opened to recruit participants, the (CI) Investigator/Principal Investigator (PI) or designee must receive a Trust confirmation of capacity and capability. It is the responsibility of the CI/ PI or designee at each site to ensure that all subsequent amendments gain the necessary approvals, including NHS Permission (where required) at the site and the HRA approvals. This does not affect the individual clinician's

Docusign Envelope ID: F60CEFFC-8A22-436F-AE83-0299BDD1E22D

responsibility to take immediate action if thought necessary to protect the health and interest of individual participants.

Within 90 days after the end of the trial, the CI/Sponsor will ensure that the main REC is notified that the trial has finished. If the trial is terminated prematurely, those reports will be made within 15 days after the end of the trial. The CI will supply the Sponsor with a summary report of the trial, which will then be submitted to the REC within 1 year after the end of the trial.

14.2 Peer Review

The trial protocol was reviewed by Novo Nordisk as part of their internal approval process. The review process included an initial UK wide review followed by a Global committee review: both processes are documented on files. In addition, as part of the sponsorship review process, the trial protocol was submitted for external. independent peer review by two experts in the field. The experts are external to the CI's host institutiion and not involved in the trial in any way. The reviewers have extensive knowledge in the field and experience of conducting clinical trials, which also allowed them to evaluate the methodological and statistical aspects of the trial.

14.3 Public and Patient Involvement

Patient and Public representatives will be involved in the management of the research with a PPI representative as a member of the TSC as well as in the dissemination of the trial findings.

14.4 Regulatory Compliance

The trial will not commence until a Clinical Trial Authorisation (CTA) is obtained from the MHRA and Favourable REC opinion. The protocol and trial conduct will comply with the Medicines for Human Use (Clinical Trials) Regulations 2004 and any relevant amendments. This trial will be conducted in compliance with the protocol, the UK Regulations, EU GCP and applicable regulatory requirement (s).

14.5 Protocol Compliance

Prospective, planned deviations or waivers to the protocol are not allowed under the UK regulations on Clinical Trials and must not be used e.g., it is not acceptable to enrol a participant if they do not meet the eligibility criteria or restrictions specified in the trial protocol.

Accidental protocol deviations can happen at any time. They must be adequately documented on the relevant forms and reported to the Chief Investigator and Sponsor immediately, as per Sponsor SOP for the Recording & Reporting of Deviations, Violations, Potential Serious breaches, Serious breaches and Urgent Safety Measures (SPON/S15).

Deviations from the protocol which are found to frequently recur are not acceptable, will require immediate action and could potentially be classified as a serious breach.

14.6 Notification of Serious Breaches to GCP and/or the protocol

A "serious breach" is a breach which is likely to effect to a significant degree –

(a) the safety or physical or mental integrity of the participants of the trial; or
(b) the scientific value of the trial

The sponsor will be notified immediately of any case where the above definition applies during the trial conduct phase as per the Sponsor SOP for the Recording & Reporting of Deviations, Violations, Potential Serious breaches, Serious breaches and Urgent Safety Measures (SPON/S15). The sponsor of a clinical trial will notify the licensing authority in writing of any serious breach of

(a) the conditions and principles of GCP in connection with that trial; or
(b) the protocol relating to that trial, as amended from time to time, within 7 days of becoming aware of that breach

14.7 Data Protection and Participant Confidentiality

The Sponsor will act as the custodian for the trial data. All data will be handled in accordance with the UK General Data Protection Regulation (UK GDPR) and the UK Data Protection Act 2018. Each participant will be given a unique trial identification number at the start and used on their records as soon as registered on the study database. Their name and other identifiable information will be kept in a separate database. Data will be held on a purpose-built database and will be password-protected, data from each individual will be listed under their PIN. The master list linking participants' name and the trial identification number will be kept in a password-protected computer. This way, participants' personal identity and data collected in the study cannot be connected by anyone outside the study team. The eCRFs will not bear the participant's name or other personal identifiable data. The subject's initials, date of birth and trial identification number will be used for identification and this will be clearly explained to participants in the PIS. Identifying participant information will be kept separate from research data. Consent forms and other paper records will be stored in locked filing cabinets in swipe-card accessed offices.

All data storage mediums will comply with the NHS Information Governance Toolkit. Confidentiality will be maintained by user agreements prior to access, ensuring all researchers uphold the principles of GCP, the EU GDPR 679/2016 and DPA 2018.

14.8 Financial and other competing interests for the Chief Investigator & PIs at each site

This trial is being funded by the Novo Nordisk. The CI and other investigators do not have any direct personal involvement in this organisation that may give rise to a possible conflict of interest.

14.9 Insurance and Indemnity

University College London holds insurance against claims from participants for injury caused by their participation in the clinical trial. Participants may be able to claim compensation if they can prove that UCL has been negligent. However, as this clinical trial is being carried out in a hospital, the hospital continues to have a duty of care to the participant of the clinical trial. University College London does not accept liability for any breach in the hospital's duty of care, or any negligence on the part of hospital employees. This applies whether the hospital is an NHS Trust or otherwise.

Participants may also be able to claim compensation for injury caused by participation in this clinical trial without the need to prove negligence on the part of University College London or another party. Participants who sustain injury and wish to make a claim for compensation should do so in writing in the first instance to the Chief Investigator, who will pass the claim to the Sponsor's Insurers, via the Sponsor's office.

Hospitals selected to participate in this clinical trial shall provide clinical negligence insurance cover for harm caused by their employees and a copy of the relevant insurance policy or summary shall be provided to University College London, upon request.

14.10 Access to the final trial dataset

The investigator/ institution will permit trial-related monitoring, audits, REC review, and regulatory inspection(s), providing direct access to source data/documents. Trial participants are informed of this during the informed consent discussion. Participants will consent to provide access to their medical notes.

15 DISSEMINATION POLICY

Upon completion of the trial and data analysis, a Final Trial Report will be prepared, and results will be disseminated. UCL, as Sponsor, will have ownership of the data. The PI and trial investigators will have rights to publish data originating from the trial. Trial results will be disseminated via publication in scientific journals and presentation at conferences. Trial participants who request copy of the study results (as expressed on the consent form) will be informed of the results of the trial via direct communication from the trial team and any publications will be made available to participants. Funders will be acknowledged in publications but will not have publishing rights independent of the PI.

15.1 Authorship eligibility guidelines and any intended use of professional writers

All trial staff who participated in the design, running and management of the trial as well as data management, analysis and dissemination will be granted authorship.

Docusign Envelope ID: F60CEFFC-8A22-436F-AE83-0299BDD1E22D

16 REFERENCES

1. Angrisani, L., et al., *Bariatric Surgery Worldwide 2013.* Obes Surg, 2015. **25**(10): p. 1822-32.
2. NICE, *NICE Clinical Guidelines [CG189]: Obesity: identification, assessment and management.* https://www.nice.org.uk/guidance/cg189, 2014.
3. Manning, S., et al., *Early postoperative weight loss predicts maximal weight loss after sleeve gastrectomy and Roux-en-Y gastric bypass.* Surg Endosc, 2015. **29**(6): p. 1484-91.
4. de Hollanda, A., et al., *Patterns of Weight Loss Response Following Gastric Bypass and Sleeve Gastrectomy.* Obes Surg, 2015. **25**(7): p. 1177-83.
5. Dimeglio, C., et al., *Weight Loss Trajectories After Bariatric Surgery for Obesity: Mathematical Model and Proof-of-Concept Study.* JMIR Med Inform, 2020. **8**(3): p. e13672.
6. Seo, D.C., et al., *The longitudinal trajectory of post-surgical % total weight loss among middle-aged women who had undergone bariatric surgery.* Prev Med Rep, 2017. **5**: p. 200-204.
7. Courcoulas, A.P., et al., *Seven-Year Weight Trajectories and Health Outcomes in the Longitudinal Assessment of Bariatric Surgery (LABS) Study.* JAMA Surg, 2018. **153**(5): p. 427-434.
8. Batterham, R.L. and D.E. Cummings, *Mechanisms of Diabetes Improvement Following Bariatric/Metabolic Surgery.* Diabetes Care, 2016. **39**(6): p. 893-901.
9. Jimenez, A., et al., *Long-term effects of sleeve gastrectomy and Roux-en-Y gastric bypass surgery on type 2 diabetes mellitus in morbidly obese subjects.* Ann Surg, 2012. **256**(6): p. 1023-9.
10. Arterburn, D.E., et al., *A multisite study of long-term remission and relapse of type 2 diabetes mellitus following gastric bypass.* Obes Surg, 2013. **23**(1): p. 93-102.
11. Lee, M.H., et al., *Predictors of long-term diabetes remission after metabolic surgery.* J Gastrointest Surg, 2015. **19**(6): p. 1015-21.
12. Pucci, A., et al., *Type 2 diabetes remission 2 years post Roux-en-Y gastric bypass and sleeve gastrectomy: the role of the weight loss and comparison of DiaRem and DiaBetter scores.* Diabet Med, 2018. **35**(3): p. 360-367.
13. Coleman, K.J., et al., *Long-term Microvascular Disease Outcomes in Patients With Type 2 Diabetes After Bariatric Surgery: Evidence for the Legacy Effect of Surgery.* Diabetes Care, 2016. **39**(8): p. 1400-7.
14. Laurino Neto, R.M., et al., *Comorbidities remission after Roux-en-Y Gastric Bypass for morbid obesity is sustained in a long-term follow-up and correlates with weight regain.* Obes Surg, 2012. **22**(10): p. 1580-5.
15. Sundbom, M., et al., *Substantial Decrease in Comorbidity 5 Years After Gastric Bypass: A Population-based Study From the Scandinavian Obesity Surgery Registry.* Ann Surg, 2016.
16. Lassailly, G., et al., *Bariatric Surgery Reduces Features of Nonalcoholic Steatohepatitis in Morbidly Obese Patients.* Gastroenterology, 2015. **149**(2): p. 379-88; quiz e15-6.
17. Caiazzo, R., et al., *Roux-en-Y gastric bypass versus adjustable gastric banding to reduce nonalcoholic fatty liver disease: a 5-year controlled longitudinal study.* Ann Surg, 2014. **260**(5): p. 893-8; discussion 898-9.
18. Mohos, E., et al., *Quality of life, weight loss and improvement of co-morbidities after primary and revisional laparoscopic roux Y gastric bypass procedure-comparative match pair study.* Obes Surg, 2014. **24**(12): p. 2048-54.
19. Raoof, M., et al., *Health-Related Quality-of-Life (HRQoL) on an Average of 12 Years After Gastric Bypass Surgery.* Obes Surg, 2015. **25**(7): p. 1119-27.
20. Pi-Sunyer, X., et al., *A Randomized, Controlled Trial of 3.0 mg of Liraglutide in Weight Management.* New England Journal of Medicine, 2015. **373**(1): p. 11-22.
21. Wharton, S., et al., *Liraglutide 3.0 mg for the management of insufficient weight loss or excessive weight regain post-bariatric surgery.* Clin Obes, 2019. **9**(4): p. e12323.
22. Suliman, M., et al., *Routine clinical use of liraglutide 3 mg for the treatment of obesity: Outcomes in non-surgical and bariatric surgery patients.* Diabetes Obes Metab, 2019. **21**(6): p. 1498-1501.
23. Hussain, A., et al., *Obesity and mortality of COVID-19. Meta-analysis.* Obes Res Clin Pract, 2020. **14**(4): p. 295-300.
24. Aminian, A., et al., *Association of prior metabolic and bariatric surgery with severity of coronavirus disease 2019 (COVID-19) in patients with obesity.* Surg Obes Relat Dis, 2021. **17**(1): p. 208-214.

Docusign Envelope ID: F60CEFFC-8A22-436F-AE83-0299BDD1E22D

25. Korakas, E., et al., *Obesity and COVID-19: immune and metabolic derangement as a possible link to adverse clinical outcomes.* Am J Physiol Endocrinol Metab, 2020. **319**(1): p. E105-e109.
26. Villarreal-Calderón, J.R., et al., *Interplay between the Adaptive Immune System and Insulin Resistance in Weight Loss Induced by Bariatric Surgery.* Oxid Med Cell Longev, 2019. **2019**: p. 3940739.
27. Beecham, J. and M. Knapp, *Costing psychiatric interventions, in G. Thornicroft (ed.) Measuring Mental Health Needs*. 2nf edition ed. 2001: Gaskell.
28. EuroQol, G., *EuroQol--a new facility for the measurement of health-related quality of life.* Health Policy, 1990. **16**(3): p. 199-208.
29. Brooks, R., *EuroQol: the current state of play.* Health Policy, 1996. **37**(1): p. 53-72.

Docusign Envelope ID: F60CEFFC-8A22-436F-AE83-0299BDD1E22D

17 Appendices

Appendix 1 – BARI-STEP Schedule of assessments

Intervention - test or assessment	Screening visit	Baseline visit (Day 1)	Week (Wk) 1	Wk 2	Wk 4	Wk 6	Wk 8	Wk 10	Wk 12
**Visit**	**Visit 1**	**Visit 2**	**Visit 3**	**Visit 4**	**Visit 5**	**Visit 6**	**Visit 7**	**Visit 8**	**Visit 9**
Visit Windows		*Day 0*	*+/- 3 days*	*+/-3 days*	*+/- 3 days*	*+/-3 days*	*+/-3 days*	*+/-3 days*	*+/-3 days*
Visit type	**SITE**	**SITE**	**REMOTE (R)**	**R**	**R**	**SITE**	**R**	**R**	**R**
**Informed Consent**	X								
**Medical history**	X	X				X			
**Physical exam**	x	x				x			
**Body composition (BIA)**		X				X			
**Height**	X								
**Weight**	X	X	X	X	X	X	X	X	X
**Urine pregnancy test (if applicable)***	X	X							
**Blood analysis**	X	X*				X			
**Resting heart rate and blood pressure**	X	X				X			
**ECG**		X							
**30-minute meal test for GLP-1**		X							
**Review of inclusion/exclusion criteria**	X								
**Subject randomisation & enrollment**		X							
**Nutritional and exercise counseling**		X		X	X		X		X
**Adverse events assessment**		X	X	X	X	X	X	X	X
**Concomitant medication review**	X	X	X	X	X	X	X	X	X
**Questionnaires (IWQOL-Lite CT, SF-36, Power of food, EQ-5D)**		X				X			
**Subcutaneous injection training**		X							
**Dispensing of trial treatment**		X				X			
**Assessment of treatment compliance /review of drug diary**			X	X	X	X	X	X	X

Intervention - test or assessment	Wk 14	Wk 16	Wk 20	Wk 24	Wk 28	Wk 32	Wk 36	Wk 40	Wk 44	Wk 48	Wk 52
**Visit**	**Visit 10**	**Visit 11**	**Visit 12**	**Visit 13**	**Visit 14**	**Visit 15**	**Visit 16**	**Visit 17**	**Visit 18**	**Visit 19**	**Visit 20**
Visit Windows	*+/- 3 days*	*+/- 3 days*	*+/- 3 days*	*+/- 3 days*	*+/- 3 days*	*+/- 3 days*	*+/- 3 days*	*+/- 7 days*	*+/- 7 days*	*+/- 7 days*	*+/- 7 days*
Visit type	**SITE**	**R**	**R**	**R**	**R**	**SITE**	**R**	**R**	**R**	**R**	**SITE**
**Physical exam**	X					X					X
**Body composition (BIA)**	X					X					X
**Weight**	X	X	X	X	X	X	X	X	X	X	X
**Urine pregnancy test (if applicable)**											
**Blood analysis**	X					X					X
**Resting heart rate and blood pressure**	X					X					X
**ECG**											
**Nutritional and exercise Counseling**		X	X	X	X	X	X	X	X	X	X
**Adverse events assessment**	X	X	X	X	X	X	X	X	X	X	X
**Concomitant medication review**	X	X	X	X	X	X	X	X	X	X	X
**Questionnaires (IWQOL-Lite CT, SF-36, Power of food, EQ-5D)**	X					X					X
**Dispensing of trial treatment**	X					X					X
**Assessment of treatment compliance /review of drug diary**	X	X	X	X	X	X	X	X	X	X	X

Docusign Envelope ID: F60CEFFC-8A22-436F-AE83-0299BDD1E22D

Intervention - test or assessment	Wk 56	Wk 60	Wk 64	Wk 68	Wk 70	Wk 74
**Visit**	**Visit 21**	**Visit 22**	**Visit 23**	**Visit 24**	**Visit 25**	**Visit 26**
Visit Windows	*+/- 7 days*	*+/- 7 days*	*+/- 7 days*	*+/- 7 days*	*+/- 7 days*	*+/- 7 days*
Visit type	**R**	**R**	**R**	**SITE**	**R**	**R**
**Physical exam**				**X**		
**Body composition (BIA)**				**X**		
**Weight**	**X**	**X**	**X**	**X**	**X**	**X**
**Urine pregnancy test (if applicable)**				**X**		
**Blood analysis**				**X**		
**Resting heart rate and blood pressure**				**X**		
**ECG**				**X**		
**Nutritional and exercise Counseling**	**X**	**X**	**X**	**X**		
**Adverse events assessment**	**X**	**X**	**X**	**X**	**X**	**X**
**Concomitant medication review**	**X**	**X**	**X**	**X**	**X**	**X**
**Questionnaires (IWQOL-Lite CT, SF-36, Power of food, EQ-5D)**				**X**		
**Dispensing of trial treatment**						
**Assessment of treatment compliance /review of drug diary**	**X**	**X**	**X**	**X**		

*pregnancy test is of a serum sample to rule out pregnancy with high accuracy.

Docusign Envelope ID: F60CEFFC-8A22-436F-AE83-0299BDD1E22D

Appendix 2 – Contraception guidance

Female participants

Females of childbearing potential are eligible to participate if they agree to use a highly effective contraception method for the duration of the trial and until 2 months after treatment discontinuation. Female participants must produce a negative pregnancy test of a serum sample at baseline to rule out pregnancy with high accuracy.

Women are considered of childbearing potential following menarche and until becoming post-menopausal unless permanently sterile. Women are considered permanently sterile if they have had documented hysterectomy, bilateral salpingectomy or bilateral oophorectomy. Postmenopausal state is defined as no menses for 12 months without any medical cause. A high Follicle Stimulating Hormone (FSH) level in the postmenopausal range may be used to confirm a postmenopausal state in women not using hormonal contraception or Hormonal Replacement Therapy (HRT). However, in the absence of 12 months of amenorrhoea, a single FSH measurement is insufficient.

Highly effective contraceptive methods include:

- Combine (oestrogen and progesterone containing) hormonal contraception associated with inhibition of ovulation:
    - Oral
    - Intravaginal
    - Transdermal
- Progesterone-only hormonal contraception associated with inhibition of ovulation:
    - Oral
    - Injectable
    - Implantable
- Intrauterine device.
- Intrauterine hormone-releasing system.
- Bilateral tubal occlusion.
- Vasectomised partner.
- True sexual abstinence (refraining from sexual intercourse – only acceptable when this is in line with the preferred and usual lifestyle of the subject).

Male participants

The effect of semaglutide on fertility in humans is unknown. Male participants with partners of childbearing potential must use barrier methods of contraception for the duration of the trial until 2 months after treatment discontinuation.
