# Supplementary material for: Semaglutide versus placebo in individuals with poor weight loss after bariatric surgery: a double-blinded, randomized, placebo-controlled trial
Source: Nat Med. 2026 May 22;32(7):2662–72. doi: 10.1038/s41591-026-04416-4 (PMC13375533; doi:10.1038/s41591-026-04416-4)
Supplement: Supplementary file 2 — Reporting Summary [file 41591_2026_4416_MOESM2_ESM.pdf]

nature portfolio

Corresponding author(s): Dr Janine Makaronidis

Last updated by author(s): Apr 11, 2026

# Reporting Summary

Nature Portfolio wishes to improve the reproducibility of the work that we publish. This form provides structure for consistency and transparency in reporting. For further information on Nature Portfolio policies, see our Editorial Policies and the Editorial Policy Checklist.

## Statistics

For all statistical analyses, confirm that the following items are present in the figure legend, table legend, main text, or Methods section.

| n/a | Confirmed | |
|---|---|---|
| ☐ | ☒ | The exact sample size ($n$) for each experimental group/condition, given as a discrete number and unit of measurement |
| ☐ | ☒ | A statement on whether measurements were taken from distinct samples or whether the same sample was measured repeatedly |
| ☐ | ☒ | The statistical test(s) used AND whether they are one- or two-sided<br>*Only common tests should be described solely by name; describe more complex techniques in the Methods section.* |
| ☐ | ☒ | A description of all covariates tested |
| ☐ | ☒ | A description of any assumptions or corrections, such as tests of normality and adjustment for multiple comparisons |
| ☐ | ☒ | A full description of the statistical parameters including central tendency (e.g. means) or other basic estimates (e.g. regression coefficient) AND variation (e.g. standard deviation) or associated estimates of uncertainty (e.g. confidence intervals) |
| ☐ | ☒ | For null hypothesis testing, the test statistic (e.g. $F$, $t$, $r$) with confidence intervals, effect sizes, degrees of freedom and $P$ value noted<br>*Give P values as exact values whenever suitable.* |
| ☒ | ☐ | For Bayesian analysis, information on the choice of priors and Markov chain Monte Carlo settings |
| ☒ | ☐ | For hierarchical and complex designs, identification of the appropriate level for tests and full reporting of outcomes |
| ☒ | ☐ | Estimates of effect sizes (e.g. Cohen's $d$, Pearson's $r$), indicating how they were calculated |

*Our web collection on statistics for biologists contains articles on many of the points above.*

## Software and code

Policy information about availability of computer code

| | |
|---|---|
| Data collection | Sealed Envelope |
| Data analysis | https://github.com/SamuelJDicken/BARI-STEP. Analyses were conducted in R (V.2024.12.1+563). |

For manuscripts utilizing custom algorithms or software that are central to the research but not yet described in published literature, software must be made available to editors and reviewers. We strongly encourage code deposition in a community repository (e.g. GitHub). See the Nature Portfolio guidelines for submitting code & software for further information.

## Data

Policy information about availability of data

All manuscripts must include a data availability statement. This statement should provide the following information, where applicable:

- Accession codes, unique identifiers, or web links for publicly available datasets
- A description of any restrictions on data availability
- For clinical datasets or third party data, please ensure that the statement adheres to our policy

The Data Availability statement ia according to ICMJE recommendations on clinical trial data sharing and provided the required information.

Will individual participant data be available: Yes

What data in particular will be shared: All of the individual participant data collected during the trial, after deidentification.

What other documents will be available: Study protocol, Statistical Analysis Plan, Patient Information Sheet – Consent Form.

When will data be available and by what mechanism: Study data can be requested following publication through the CI/corresponding author. Depending on the nature of the request, in line with ethics approvals, the study protocol and participant consent form, further approvals may be required.

## Research involving human participants, their data, or biological material

Policy information about studies with human participants or human data. See also policy information about sex, gender (identity/presentation), and sexual orientation and race, ethnicity and racism.

| | |
|---|---|
| Reporting on sex and gender | We report biological sex based on healthcare records. 58 [82.9%] participants were female and the remaining 12 [17.1%] were male. The randomisation procedure was stratified by sex. Our findings apply to both sexes.<br><br>Participants provided fully informed written consent and consented to sharing anonymised trial data only.<br><br>The primary outcome was analysed using a linear regression model, adjusted for sex (and other covariates) following the intention-to-treat (ITT) principle.<br>Secondary outcomes were analysed on an ITT basis using linear regression, adjusted for sex (and other covariates) . |
| Reporting on race, ethnicity, or other socially relevant groupings | Ethnicity was self-reported from participants at randomisation. Randomisation was not stratified by ethnicity. For reporting baseline characteristics and exploratory analyses, participants were categorised into four ethnicity groups 'Asian, Black, White and Mixed/Other' in order to prevent small sample sizes within each subgroup.<br><br>Ethnicity was self- reported as:<br>- English/Welsh/Scottish/Northern Irish/British<br>- Caribbean<br>- African<br>- White & Black Caribbean<br>- White & Asian<br>- Any other Black / African / Caribbean background<br>- White & Black African<br>- Any other ethnic group<br>- Any other White background<br>- Indian<br>- Any other Asian background<br>- Romani or Irish Traveller |
| Population characteristics | 70 adult (age 18-65 inclusive) patients , ≥1 year primary gastric bypass or primary sleeve gastrectomy with a suboptimal clinical response(<20% weight loss since their day of surgery) that is not caused by either a surgical or psychological problem. The mean age 47.3 years [standard deviation, SD ±10.3], 58 [82.9%] participants were female and the remaining 12 [17.1%] were male. |
| Recruitment | BARI-STEP was conducted at University College London Hospitals (UCLH). Participants were recruited from the Bariatric Clinics at UCLH and Homerton University Hospital. Participants were enrolled between 18 November 2022 and 02 November 2023. Patients were referred from bariatric clinics and MDT and assessed for eligibility based on the inclusion/exclusion criteria. The bariatric population in our service has a high proportion of female patients and this is reflected in our study population, which reflects the population seen in the clinical services. |
| Ethics oversight | The trial was approved by the London Surrey Borders Research Ethics Committee (22/LO/0045) and was conducted in accordance with the Declaration of Helsinki, the principles of Good Clinical Practice and all applicable regulatory requirements, including the Research Governance Framework and the Medicines for Human Use (Clinical Trial) Regulations 2004 and any subsequent amendments. The trial was registered at ClinicalTrials.gov (Clinicaltrials.gov identifier: NCT05073835), the UK Medicines and Healthcare products Regulatory Agency (MHRA), and the European Union Drug Regulating Authority Clinical Trials (EudraCT 2021-004568-83). The study followed the Consolidated Standards of Reporting Trials (CONSORT) reporting guideline. |

Note that full information on the approval of the study protocol must also be provided in the manuscript.

# Field-specific reporting

Please select the one below that is the best fit for your research. If you are not sure, read the appropriate sections before making your selection.

☒ Life sciences ☐ Behavioural & social sciences ☐ Ecological, evolutionary & environmental sciences

For a reference copy of the document with all sections, see nature.com/documents/nr-reporting-summary-flat.pdf

# Life sciences study design

All studies must disclose on these points even when the disclosure is negative.

| | |
|---|---|
| Sample size | A sample size calculation was conducted using the 24-week primary ITT analysis from our BARI-OPTIMISE trial 14and the results of the Davies et al. 27 multi-centre trial of Semaglutide 3.0 mg/ml in adults with overweight or obesity, and T2D. Assuming a SD for %WL of 4.0, dropout rate of 10%, and 1% critical significance level, 62 patients (31 per group) would provide at least 95% power to detect an estimated difference of 10%WL with a 95% CI no wider than 8.0 to 12.0. The recruitment target was set at 35 participants per group. |
| Data exclusions | The ITT population (n=63) excluded those who withdrew or lost to follow up. Two participants (both in the placebo arm) were lost to follow up. Five participants in total withdrew from the trial. This included four in the placebo arm (two participants did not provide a reason for withdrawal, one participant did not have a weight loss response, and the remaining participant withdrew due to adverse event [AE] reasons). One participant in the Semaglutide 2.4mg arm was withdrawn due to an investigator decision as a result of a Suspected Unexpected Serious Adverse Reaction (SUSAR).<br><br>The participants in the per-protocol (PP) analysis had full adherence to the study protocol, which was defined as attending all study visits with no significant interruptions in treatment as monitored from the drug dose diary. The remaining six participants included in the ITT but excluded from the PP analysis did not satisfy the definition of full adherence, and therefore did not meet the conditions to be included in PP analysis. Reasons included a prolonged period of time off drug treatment (n=5) and an apronectomy (n=1) during the trial. |
| Replication | Data analyses were carried out by the research team and our independent statistician achieved the same results. This is a single clinical trial of human participants and therefore no replicates measurements were performed. Biological samples analysed in the laboratory for GLP-1 levels were run in duplicate. |
| Randomization | Participants were randomly assigned in a 1:1 ratio to receive either Semaglutide 2.4 mg or placebo. Randomisation was carried out by a computer-generated randomisation sequence (Sealed Envelope) stratified by surgical procedure type (GB vs SG), sex and T2D status. The primary outcome was %WL following 68 weeks of treatment. Clinical study personnel were blinded to the randomisation procedure until after the trial had ended. |
| Blinding | Clinical study personnel  and participants were blinded to the randomisation procedure until after the trial had ended. |

# Reporting for specific materials, systems and methods

We require information from authors about some types of materials, experimental systems and methods used in many studies. Here, indicate whether each material, system or method listed is relevant to your study. If you are not sure if a list item applies to your research, read the appropriate section before selecting a response.

## Materials & experimental systems

| n/a | Involved in the study | |
|---|---|---|
| ☒ | ☐ | Antibodies |
| ☒ | ☐ | Eukaryotic cell lines |
| ☒ | ☐ | Palaeontology and archaeology |
| ☒ | ☐ | Animals and other organisms |
| ☐ | ☒ | Clinical data |
| ☒ | ☐ | Dual use research of concern |
| ☒ | ☐ | Plants |

## Methods

| n/a | Involved in the study | |
|---|---|---|
| ☒ | ☐ | ChIP-seq |
| ☒ | ☐ | Flow cytometry |
| ☒ | ☐ | MRI-based neuroimaging |

## Clinical data

Policy information about clinical studies

All manuscripts should comply with the ICMJE guidelines for publication of clinical research and a completed CONSORT checklist must be included with all submissions.

| | |
|---|---|
| Clinical trial registration | Clinicaltrials.gov identifier: NCT05073835 |
| Study protocol | The study protocol can be accessed within the Appendix. |
| Data collection | BARI-STEP was conducted at University College London Hospitals (UCLH). Participants were recruited from the Bariatric Clinics at UCLH and Homerton University Hospital. Participants were enrolled between 18 November 2022 and 02 November 2023. |
| Outcomes | The primary objective was to compare the efficacy of 68 weeks of subcutaneous Semaglutide 2.4 mg weekly versus placebo administration, as an adjunct to diet and exercise, on %WL in participants with <20% weight-loss following primary GB or SG at the end of the 68 weeks of treatment. The pre-specified primary outcome was the difference in mean percentage body weight change between patients randomized to Semaglutide versus placebo at 68 weeks.<br>Pre-specified secondary outcomes included change in body weight (kg) from baseline and the proportion of participants who after 68 weeks achieved a body weight reduction ≥10%, ≥15%, and ≥20%. Non-pre-specified outcomes included fat mass, lean soft tissue mass, lean soft tissue mass (%), BMI and HR. Metabolic secondary outcomes included HbA1c,, blood pressure (systolic and diastolic), |

heart rate, total cholesterol, triglycerides, hsCRP, HbA1c in participants with pre-existing pre-diabetes and T2D, BP in participants with pre-existing hypertension and changes in T2D and hypertension medication used in participants with T2D and hypertension, respectively. Health-related quality of life was also assessed at the in-person visits using the Impact of Weight on Quality of Life-Lite questionnaire 42. Health economic analyses, inflammatory markers and other questionnaire data will be reported separately.

Weight and body composition measures were assessed at six time points (baseline and week 6, 14, 32, 52 and 68) using Bioelectrical Impedance Analysis (BIA, Tanita DC-430MAS) to measure weight, lean soft tissue mass and fat mass. Participants were given consistent instructions for hydration status at visits to enhance BIA accuracy, and all participants were advised consistently to avoid intense exercise, alcohol and caffeine before their study visit. At the six in person visits, metabolic secondary outcomes were measured including HbA1c,, blood pressure (systolic and diastolic), heart rate, total cholesterol, triglycerides, hsCRP, HbA1c in participants with pre-existing pre-diabetes and T2D, BP in participants with pre-existing hypertension and changes in T2D and hypertension medication used in participants with T2D and hypertension, respectively. Health-related quality of life was also assessed at the in-person visits using the Impact of Weight on Quality of Life-Lite questionnaire 42.

## Plants

| | |
|---|---|
| Seed stocks | N/A |
| Novel plant genotypes | N/A |
| Authentication | N/A |
